# A novel terpene synthase controls differences in anti-aphrodisiac pheromone production between closely related *Heliconius* butterflies

**DOI:** 10.1371/journal.pbio.3001022

**Published:** 2021-01-19

**Authors:** Kathy Darragh, Anna Orteu, Daniella Black, Kelsey J. R. P. Byers, Daiane Szczerbowski, Ian A. Warren, Pasi Rastas, Ana Pinharanda, John W. Davey, Sylvia Fernanda Garza, Diana Abondano Almeida, Richard M. Merrill, W. Owen McMillan, Stefan Schulz, Chris D. Jiggins

**Affiliations:** 1 Department of Zoology, University of Cambridge, Cambridge, United Kingdom; 2 Smithsonian Tropical Research Institute, Panamá, Panamá; 3 School of Biology, University of St Andrews, St Andrews, United Kingdom; 4 Institute of Organic Chemistry, Department of Life Sciences, Technische Universität Braunschweig, Braunschweig, Germany; 5 Institute of Biotechnology, University of Helsinki, Helsinki, Finland; 6 Division of Evolutionary Biology, Ludwig-Maximilians-Universität München, Munich, Germany; University of Lausanne, SWITZERLAND

## Abstract

Plants and insects often use the same compounds for chemical communication, but not much is known about the genetics of convergent evolution of chemical signals. The terpene (*E*)-β-ocimene is a common component of floral scent and is also used by the butterfly *Heliconius melpomene* as an anti-aphrodisiac pheromone. While the biosynthesis of terpenes has been described in plants and microorganisms, few terpene synthases (TPSs) have been identified in insects. Here, we study the recent divergence of 2 species, *H*. *melpomene* and *Heliconius cydno*, which differ in the presence of (*E*)-β-ocimene; combining linkage mapping, gene expression, and functional analyses, we identify 2 novel TPSs. Furthermore, we demonstrate that one, HmelOS, is able to synthesise (*E*)-β-ocimene in vitro. We find no evidence for TPS activity in HcydOS (HmelOS ortholog of *H*. *cydno*), suggesting that the loss of (*E*)-β-ocimene in this species is the result of coding, not regulatory, differences. The TPS enzymes we discovered are unrelated to previously described plant and insect TPSs, demonstrating that chemical convergence has independent evolutionary origins.

## Abstract 2

Plants and insects often use the same compounds for chemical communication, but little is known about the convergent evolution of such chemical signals. This study identifies a novel terpene synthase involved in production of an anti-aphrodisiac pheromone by the butterfly Heliconius melpomene. This enzyme is unrelated to other insect terpene synthases, providing evidence that the ability to synthesise terpenes has arisen multiple times independently within the insects.

## Introduction

Plants and insects often use the same compounds for communication [1,2]. In many cases, this convergent evolution may be an adaptation to exploit preexisting sensory traits in the intended receiver. For example, sexually deceptive orchids mimic the scent of female insects to attract males for pollination [3]. Similarly, insects may use plant-like volatiles as sex pheromones to exploit sensory systems which have evolved for plant finding [2,4,5]. Phenotypic convergence such as this may involve different molecular mechanisms, including independent evolution at different loci or the exchange of genes through horizontal gene transfer [6]. However, although the genetic basis of convergent evolution has been studied across a range of organisms [6–10], we know little about the genetic basis of convergence in chemical signals, perhaps the most commonly used sensory modality [11].

One example of chemical convergence between plants and insects is the use of β-ocimene, a very common plant volatile, suggested to be important in pollinator attraction due to its abundance and ubiquity in floral scents [12]. This compound is also found in the genitals of male *Heliconius* butterflies [13–15]. In *Heliconius melpomene*, (*E*)-β-ocimene acts as an anti-aphrodisiac pheromone, transferred from males to females during mating to repel further courtship from subsequent males [13]. β-Ocimene is also found in large amounts in the flowers on which adult *H*. *melpomene* feed and elicits a strong antennal response in both males and females [16,17]. This compound, therefore, appears to be carrying out 2 context-dependent functions, attraction to plants and repulsion from mated females.

Anti-aphrodisiac pheromones vary both qualitatively and quantitatively between *Heliconius* species [18]. Some compounds are only found in particular clades or species, while others, such as (*E*)-β-ocimene, are found in distantly related *Heliconius* species. This phylogenetic pattern suggests that these pheromones evolve rapidly, with gains and losses common throughout the evolutionary history of *Heliconius* [14]. *Heliconius cydno*, a species closely related to *H*. *melpomene*, does not produce (*E*)-β-ocimene [14,18], most likely representing a loss of (*E*)-β-ocimene production as this compound is present in other *Heliconius* species. This provides us with the opportunity to study the genetic basis of this rapidly evolving trait between species.

**Fig 1:**
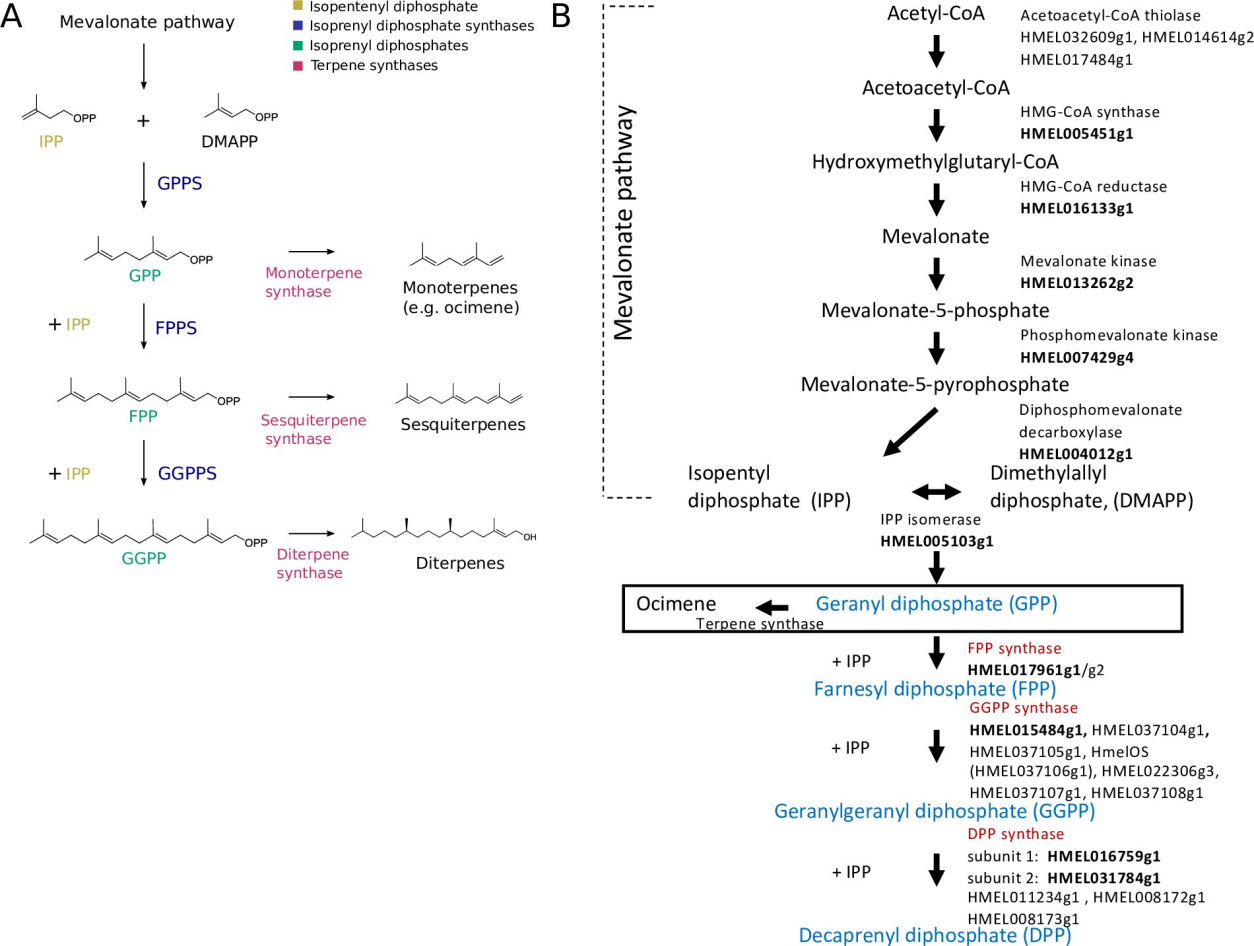
Pathway of terpene biosynthesis. (A) IPP and DMAPP are first formed from the mevalonate pathway. IPP and DMAPP are the substrates for isoprenyl disphosphate synthases (GPPS, FPPS, and GGPPS). IDSs produce isoprenyl diphosphates of varying lengths, depending on the number of IPP units added. Isoprenyl diphosphates (GPP, FPP, and GGPP) are themselves the substrates used by TPSs to make terpenes of various sizes. For example, monoterpene synthases produce monoterpenes, such as ocimene, from GPP. For illustration, (*E*,*E*)-α-farnesene is used as a representative sesquiterpene, and phytol as a diterpene. (B) Proposed biosynthetic pathway in *H*. *melpomene*. Reciprocal best BLAST hits are highlighted in bold. IDSs are in red and their products, isoprenyl diphosphates, in blue. BLAST, basic local alignment search tool; DMAPP, dimethylallyl diphosphate; FPP, farnesyl diphosphate; FPPS, farnesyl diphosphate synthase; GGPP, geranylgeranyl diphosphate; GGPPS, geranylgeranyl diphosphate synthase; GPP, geranyl diphosphate; GPPS, geranyl diphosphate synthase; IDS, isoprenyl diphosphate synthase; IPP, isopentenyl diphosphate; TPS, terpene synthase.

Although β-ocimene synthases have been described in plants, none have been found in animals [12]. It has previously been shown that *H*. *melpomene* is able to synthesise (*E*)-β-ocimene de novo [13]. β-Ocimene is a monoterpene, a member of the largest and most structurally diverse class of natural products, the terpenes [19]. Terpenes are formed from 2 precursors, the 5-carbon molecules isopentenyl diphosphate (IPP) and dimethylallyl diphosphate (DMAPP). Varying numbers of IPP units are added to DMAPP to form isoprenyl diphosphates of different chain lengths by isoprenyl diphosphate synthases (IDSs) [20,21] (Fig 1A). These isoprenyl diphosphates are the precursors for the production of terpenes by terpene synthases (TPSs), with the length of the isoprenyl diphosphate determining the type of terpene that is made [22,23] (Fig 1A). TPSs had only been described in plants and fungi in the eukaryotic domain [24] until recently, when insect TPS genes were discovered in Hemiptera and Coleoptera [1,25–29]. These TPS genes are not homologous to plant TPSs, and instead have evolved from IDS-like genes, most closely related to farnesyl diphosphate synthases (FPPSs) [1,27]. It is unclear whether the evolution of TPS activity occurred only once in insects, as the most recent phylogenetic evidence suggests or has occurred independently in different lineages [1,29].

Here, we identify the genes involved in the biosynthesis of (*E*)-β-ocimene in the butterfly *H*. *melpomene* and analyse the evolution of terpene synthesis in *Heliconius* and other insects. To determine candidate TPS genes, we identified pathway orthologs in *H*. *melpomene* and carried out a genetic mapping study between *H*. *melpomene* and *H*. *cydno*. We identified a genomic region associated with the production of (*E*)-β-ocimene and searched for candidates within this region. We then identified genes with up-regulated expression in the genitals of male *H*. *melpomene*, where (*E*)-β-ocimene is produced. We tested the TPS function of our candidate genes, as well as an ortholog in *H*. *cydno*, by expression in *Escherichia coli* followed by enzymatic assays. We carried out phylogenetic analyses, selection models, and ancestral state reconstruction, to place our discoveries relative to previously identified plant and insect TPS genes.

## Results

### Expansion of IDSs in genome of *H*. *melpomene*

We identified candidates potentially involved in terpene synthesis by searching in the genome of *H*. *melpomene* for enzymes in the mevalonate pathway and IDSs using well-annotated *Drosophila melanogaster* orthologs (S1 Table) [30–32]. We identified reciprocal best basic local alignment search tool (BLAST) hits for all enzymes, except for acetoacetyl-CoA thiolase, which showed the closest similarity to 3 *Heliconius* genes with no reciprocal best BLAST hit (Fig 1B). There was a clear one-to-one relationship for all enzymes, except for the IDSs which showed evidence for gene duplication. Of these, *Heliconius* contains 2 putative FPPSs, 4 putative copies of decaprenyl diphosphate synthase (DPPS) subunit 2, and 7 putative geranylgeranyl diphosphate synthases (GGPPSs) (Fig 1A and 1B).

The biggest expansion found was that of the GGPPSs, which are IDSs that catalyse the addition of IPP to farnesyl diphosphate (FPP) to form geranylgeranyl diphosphate (GGPP). One of these, *HMEL015484g1*, shows 83% amino acid sequence similarity to the GGPPS of the moth *Choristoneura fumiferana*, which has previously been characterised in vitro to catalyse the production of GGPP from FPP and IPP [33]. *HMEL015484g1* is also the best reciprocal BLAST hit with the GGPPS of *D*. *melanogaster* (Fig 1A and 1B). The other 6 annotated GGPPSs show less than 50% similarity to the moth GGPPS, such that their function is less clear.

### QTL for (*E*)-β-ocimene production on chromosome 6

**Fig 2:**
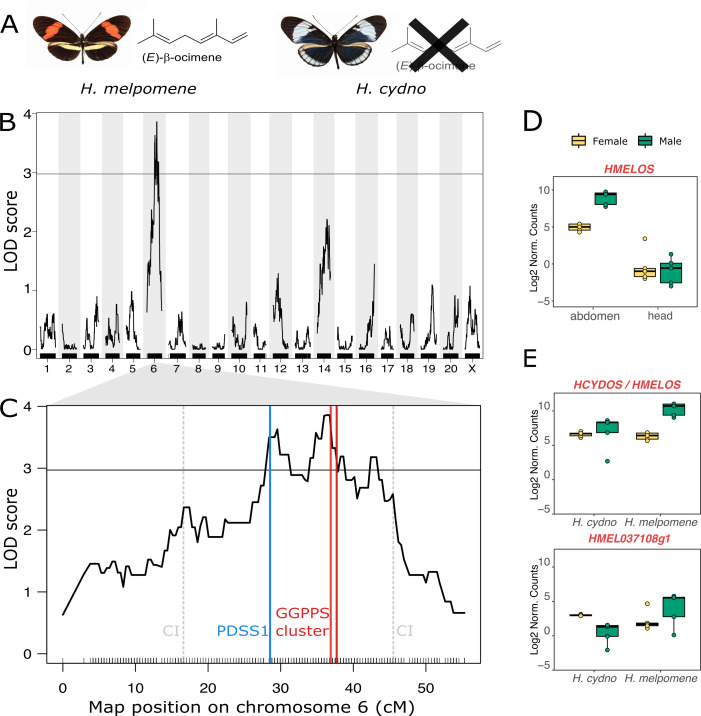
QTL and gene expression analyses to identify candidate genes for (*E*)-β-ocimene production. (A) The 2 species used in the crosses, *H*. *melpomene* which produces (*E*)-β-ocimene and *H*. *cydno* which does not. (B) Genome-wide scan for QTL underlying (*E*)-β-ocimene production. (C) QTL on chromosome 6 for (*E*)-β-ocimene production. CIs as well as the positions of candidate genes (subunit 1 of DPPS (PDSS1) and the GGPPS cluster) in the region are marked. Black lines above x-axis represent genetic markers, and horizontal line shows genome-wide significance threshold (alpha = 0.05, LOD = 2.97). (D) *HMELOS* in *H*. *melpomene* shows male abdomen-biased expression (for expression of other genes, see S3 Fig). (E) *HMELOS* and *HMEL037108g1* both show greater male-biased expression in *H*. *melpomene* than *H*. *cydno* (for expression of other genes, see S4 Fig). Full model statistics in S2 and S3 Tables. *N* = 5 for each boxplot. Gene expression is given in log2 of normalised counts per million (using the TMM transformation). Sequencing data used to make linkage maps are available from the ENA study PRJEB34160. RNA-seq data of *H*. *cydno* and *H*. *melpomene* heads and abdomens was obtained from GenBank BioProject PRJNA283415. Processed data and scripts are available from OSF (https://osf.io/3z9tg/). CI, confidence interval; ENA, European Nucleotide Archive; LOD, log odds ratio; QTL, quantitative trait locus; RNA-seq, RNA sequencing; TMM, trimmed mean of M values.

In order to determine which of the genes identified above could be important for (*E*)-β-ocimene production in *H*. *melpomene*, we generated genetic mapping families composed of crosses between 2 closely related species that differ in presence/absence of (*E*)-β-ocimene, *H*. *melpomene*, and *H*. *cydno* (Fig 2A). These 2 species can hybridise and, although the F1 females are sterile, F1 males can be used to generate backcross hybrids. We bred interspecific F1 hybrid males and backcrossed these with virgin females of both species to generate a set of backcross mapping families. The (*E*)-β-ocimene phenotype segregated in families backcrossed to *H*. *cydno*, and so we focused on these families (S1 Fig). Using quantitative trait locus (QTL) mapping with 114 individuals, we detected a single significant peak on chromosome 6 associated with (*E*)-β-ocimene quantity (Fig 2B). The QTL peak was at 36.4 cM, and the associated confidence interval spans 16.7 to 45.5 cM, corresponding to a 6.89-Mb region containing hundreds of genes. The percentage of phenotypic variance explained by the peak marker is 16.4%, suggesting that additional loci and/or environmental factors also contribute to the phenotype (S2 Fig).

### Patterns of gene expression identify *HMELOS* and *HMEL037108g1* as candidates

To identify candidate genes for (*E*)-β-ocimene production, we searched within the confidence interval of the QTL peak. We found that subunit 1 of DPPS as well as all 7 GGPPSs were found in this region (Fig 2C). We then compared the expression levels of the 8 genes found within the QTL using published RNA sequencing (RNA-seq) data [34]. We first analysed data from *H*. *melpomene* male and female abdomens and heads, mapped to the *H*. *melpomene* reference genome (v2.5). Since (*E*)-β-ocimene is found in male abdomens in *H*. *melpomene*, we hypothesised that its synthase would be highly expressed in this sex and tissue. Only 1 gene showed male abdomen-biased expression: *HMELOS (*previously *HMEL037106g1* from the *H*. *melpomene* annotation) (tissue * sex, t = −4.18, adjp = 0.0029; Fig 2D and S2 Table). All other genes did not show a significant bias in this direction (S3 Fig and S2 Table).

We next compared gene expression between *H*. *cydno* and *H*. *melpomene* abdomens. If HmelOS is synthesising (*E*)-β-ocimene, we might expect *HMELOS* expression to be higher in *H*. *melpomene* male abdomens than in *H*. *cydno*, given that *H*. *cydno* does not produce the compound. We generated a reference-guided assembly of *H*. *cydno* by aligning an existing *H*. *cydno* Illumina trio assembly [35] to the *H*. *melpomene* reference, followed by automated gene annotation. We then manually identified *H*. *cydno* orthologs for our 7 candidate genes and checked for differential expression between species and sexes. *HMELOS* and *HMEL037108g1* were the only genes showing greater male-biased expression in *H*. *melpomene* abdomens than in *H*. *cydno* abdomens (*HMELOS*, species * sex, t = 3.15, adjp = 0.0445; *HMEL037108g1*, species * sex, t = 3.44, adjp = 0.0259; Fig 2E and S3 Table). No other genes showed a significant bias in this direction (S4 Fig and S3 Table). In summary, *HMELOS* and to a lesser extent *HMEL037108g1* are primary candidate genes from within the QTL region.

### Functional characterisation demonstrates the TPS activity of HmelOS and HMEL037108g1

**Fig 3:**
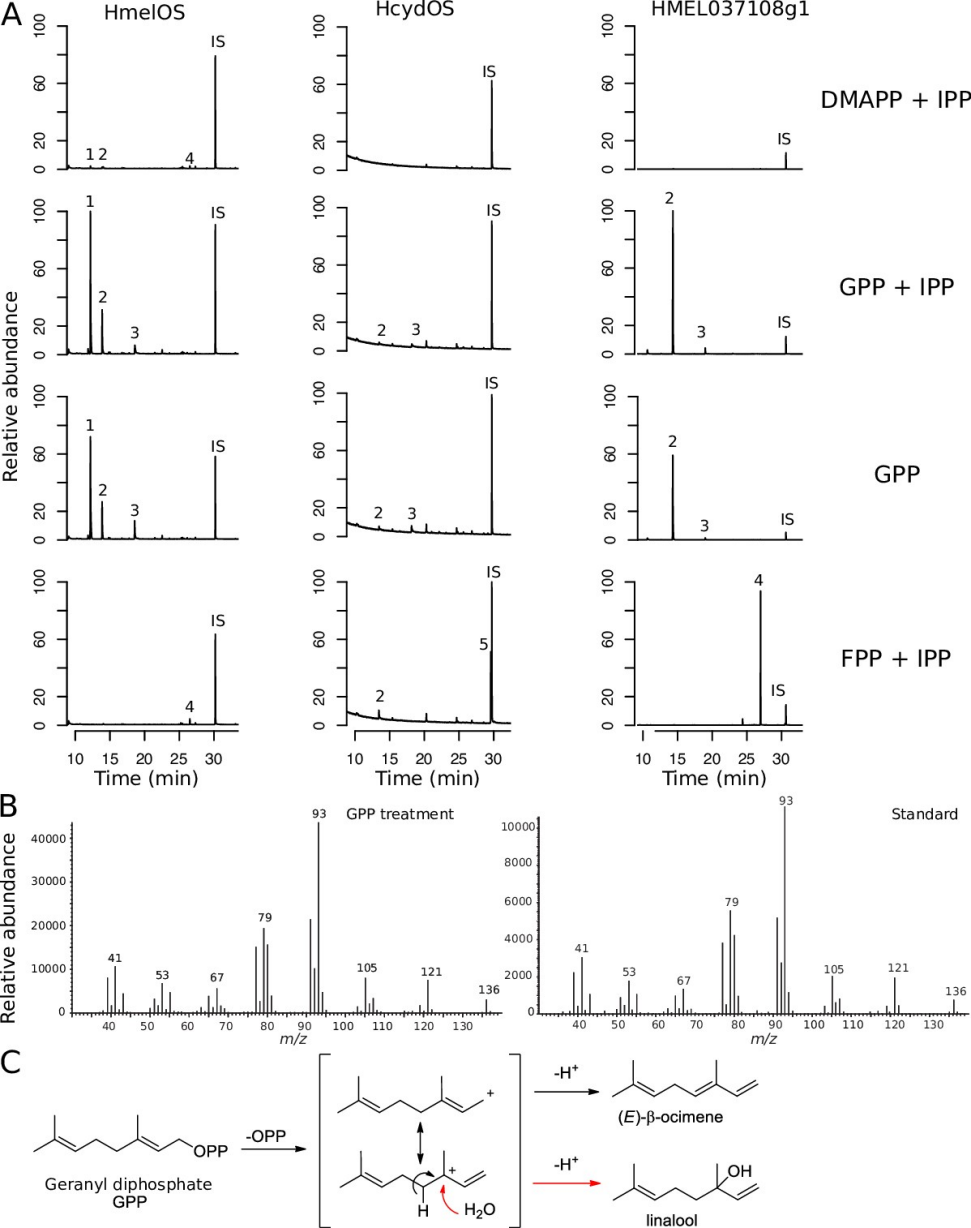
Functional characterisation of TPS activity of HmelOS and HMEL037108g1 from *H*. *melpomene* and HcydOS from *H*. *cydno*. (A) Total ion chromatograms of enzyme products in the presence of different precursor compounds. HmelOS produces high amounts of (*E*)-β-ocimene in the presence of GPP, with trace amounts found in the treatment with DMAPP + IPP and none with FPP. HMEL037108g1 produces large amounts of linalool with GPP and nerolidol with FPP. HcydOS does not exhibit TPS activity, showing no difference from control treatments (see S6 Fig). 1, (*E*)-β-Ocimene; 2, Linalool; 3, Geraniol; 4, Nerolidol; 5, Farnesol; IS, internal standard. Abundance is scaled to the highest peak of all treatments per enzyme. For quantification of peaks, see S4–S6 Tables. (B) Confirmation of identity of (*E*)-β-ocimene by comparison of mass spectra of (*E*)-β-ocimene produced in experiments and a standard. Chromatograms and mass spectra of all standards can be found in S12 Fig. (C) Pathway of how (*E*)-β-ocimene and linalool are formed from GPP. Raw data are available from OSF (https://osf.io/3z9tg/). DMAPP, dimethylallyl diphosphate; FPP, farnesyl diphosphate; GPP, geranyl diphosphate; IPP, isopentenyl diphosphate; TPS, terpene synthase.

We cloned *HMELOS* and *HMEL037108g1* from *H*. *melpomene*, as well as the *H*. *cydno* ortholog of *HMELOS*, *HCYDOS*, into plasmids and were able to generate heterologous expression of both proteins in *E*. *coli* (S5 Fig). We then conducted enzymatic assays with the expressed proteins using precursors from different points in the pathway to characterise their enzymatic function (Figs 1 and 3).

First, we carried out assays with DMAPP and IPP, the 2 building blocks at the beginning of the terpene synthesis pathway to test for both IDS and TPS activity, as was seen in *Ips pini* (Fig 1). HmelOS produced trace amounts of (*E*)-β-ocimene, linalool (another monoterpene), and nerolidol (a sesquiterpene), in this assay. This presumably occurs via the production of geranyl diphosphate (GPP) and FPP; therefore, HmelOS exhibits residual GPPS and FPPS activity, as well as monoterpene synthase and sesquiterpene synthase activity to convert the GPP and FPP to (*E*)-β-ocimene, linalool, and nerolidol (Fig 3A, S6 Fig and S4 Table). HMEL037108g1 produced trace amounts of linalool (Fig 3A and S6 Fig) and nerolidol from DMAPP and IPP. Again, this demonstrates residual GPS and FPPS activity to form the GPP and FPP and then both monoterpene and sesquiterpene synthase activity to convert these to linalool and nerolidol (Fig 3A and S5 Table). We found no evidence for activity of HcydOS (Fig 3A, S6 Fig and S6 Table).

We then carried out assays with GPP and IPP, as well as GPP alone to test for monoterpene synthase activity (Fig 1). Assaying with GPP and IPP together also allows us to test for potential FPPS and sesquiterpene synthase activity which could occur via production of FPP. HmelOS showed monoterpene synthase activity, producing (*E*)-β-ocimene when provided with either GPP and IPP, or GPP alone (Fig 3A, S6 Fig and S4 Table). Small amounts of (*Z*)-β-ocimene were also produced in treatments where (*E*)-β-ocimene was produced in large quantities (S4 Table). In contrast to HmelOS, HMEL037108g1 only produced (*E*)-β-ocimene in very small amounts from GPP (S5 Table). Instead, linalool was produced in large amounts from GPP, suggesting that this enzyme is also acting as a monoterpene synthase but is responsible for production of linalool rather than (*E*)-β-ocimene (Fig 3A, S6 Fig and S5 Table). HmelOS also produced linalool, albeit in much smaller quantities (Fig 3A, S6 Fig and S4 Table). We found no evidence for monoterpene activity of HcydOS (Fig 3A, S6 Fig and S6 Table).

Finally, we carried out assays with FPP and IPP to test for sesquiterpene synthase activity (Fig 1). Although HmelOS exhibited small amounts of sesquiterpene synthase activity through the trace production of nerolidol when provided with DMAPP and IPP (S4 Table), when provided with FPP and IPP, sesquiterpene synthase activity was not demonstrated, suggesting that it is not the primary enzyme function (Fig 3A, S6 Fig, and S4 Table). In contrast, HMEL037108g1 did exhibit sesquiterpene synthase activity, producing large amounts of nerolidol when FPP was provided as a precursor (Fig 3A, S6 Fig and S5 Table). We found no evidence for sesquiterpene synthase activity of HcydOS (Fig 3A, S6 Fig and S6 Table).

Due to the linalool detected in treatments where (*E*)-β-ocimene was produced by HmelOS, we tested whether linalool could be a metabolic intermediate between GPP and (*E*)-β-ocimene. However, HmelOS did not produce (*E*)-β-ocimene from linalool (S7 Fig and S7 Table). The 2 stereoisomers of linalool, (*S*)-linalool and (*R*)-linalool, have different olfactory properties. We confirmed the stereochemistry of linalool produced by both enzymes and found that while HmelOS produced mainly (*S*)-linalool, HMEL037108g1 produced a racemic mixture (S8 Fig).

In summary, HmelOS is a monoterpene synthase, catalysing the conversion of GPP to (*E*)-β-ocimene in *H*. *melpomene* (Fig 3B and 3C and S8 Table). HMEL037108g1 is a bifunctional monoterpene and sesquiterpene synthase catalysing the conversion of GPP to linalool as well as FPP to nerolidol (Fig 3C and S8 Table). We found no evidence for TPS activity of HcydOS (Fig 3A, S6 Fig and S8 Table).

### Functional characterisation demonstrates the residual IDS activity of HmelOS, HcydOS, and HMEL037108g1

In order to better understand the origin of TPS activity in these enzymes, we further investigated their residual IDS activity. While the production of terpenes can be tested by direct gas chromatography/mass spectrometry (GC/MS) analysis of the products of each experiment, this method will not detect isoprenyl diphosphates, potentially missing IDS activity if it is present. In order to test for IDS activity, we repeated the above experiments with DMAPP and IPP, GPP and IPP, and FPP and IPP, followed by treatment with alkaline phosphatase to hydrolyse the isoprenyl diphosphate products to their respective alcohols. These alcohols can then be detected by GC/MS analysis.

No further IDS activity was detected in any enzyme, apart from the residual IDS activity of the *H*. *melpomene* enzymes already determined above due to the trace amounts of terpenes produced from DMAPP and IPP. When either enzyme is provided with GPP, geraniol is produced, and when provided with FPP, large amounts of farnesol is produced, as expected from the dephosphorylation of the provided precursors, and this is seen in control conditions, as well all experiments with HcydOS (S9–S11 Figs and S9–S11 Tables). As expected from the previous experiments, (*E*)-β-ocimene is also produced when HmelOS is provided with GPP, and linalool and nerolidol are produced when HMEL037108g1 is provided with GPP and FPP, respectively. Geranylgeraniol is not produced in any treatments, demonstrating that neither HmelOS, HMEL037108g1 nor HcydOS is a GGPPS, as suggested by their annotation (S9–S11 Figs and S9–S11 Tables). In summary, both HmelOS and HMEL037108g1 only exhibit residual IDS activity, while we did not detect any IDS activity for HcydOS.

### Evolutionary history of gene family containing *Heliconius* TPSs

Lineage-specific expansions of gene families are often correlated with functional diversification and the origin of novel biological functions [36]. We therefore carried out a phylogenetic analysis of GGPPS in Lepidoptera to investigate whether gene duplication could have played a role in the evolution of the TPSs HmelOS and HMEL037108g1. Orthologs of the *H*. *melpomene* GGPPSs were identified in *H*. *cydno*, *Heliconius erato*, *Bicyclus anynana*, *Danaus plexippus*, *Papilio polytes*, *Pieris napi*, *Manduca sexta*, *Bombyx mori*, and *Plutella xylostella* [37]. Expansions of the GGPPS group of enzymes can be seen in *Heliconius* and in *Bicyclus*, both groups in which terpenes form part of the pheromone blend [38] (S13 Fig). Expansion of gene families is often followed by an acceleration of the nonsynonymous mutation rate facilitated by a relaxation or loss of selectional constraints [39]. To further investigate whether gene duplication played a role in the evolution of TPSs, we examined patterns of synonymous to non-synonymous evolution in the *Heliconius* GGPPS/TPS family. *Heliconius* and *Bicyclus* clades show higher relative non-synonymous evolution consistent with relaxed selection constraints (ω = 0.22 and 0.19, respectively) compared to the strong purifying selection on the non-expanded lepidopteran GGPPS genes (ω = 0.027, χ2 = 338.2, *p* < 0.00001).

To focus on the *Heliconius*-specific duplications, we made a phylogeny using the DNA sequence of transcripts from *H*. *melpomene*, *H*. *cydno*, and *H*. *erato*. *H*. *melpomene* and *H*. *cydno* belong to the same clade within *Heliconius*, with an estimated divergence time around 1.5 million years ago [40]. *H*. *erato* is more distantly related, belonging to a different *Heliconius* clade which diverged from the *H*. *melpomene*/*H*. *cydno* group around 10 million years ago [41]. While (*E*)-β-ocimene is not found in the genitals of *H*. *cydno*, it is found in the genitals of *H*. *erato*, at around one-tenth the amount of *H*. *melpomene* [18]. We hypothesised that duplications between the *H*. *melpomene* and *H*. *erato* clades may have resulted in subfunctionalisation and a more efficient *H*. *melpomene* enzyme facilitating increased (*E*)-β-ocimene production. We found that both losses and gene duplications have occurred between the *H*. *melpomene* and *H*. *erato* clades, while gene copy number is conserved between closely related *H*. *melpomene* and *H*. *cydno* (S14 Fig). The exact orthology between the *H*. *erato* and *H*. *melpomene*/*H*. *cydno* genes is unclear, but *H*. *melpomene*/*H*. *cydno* have more genes in this family than *H*. *erato* (S14 Fig), and both clades have more genes than the ancestral lepidopteran state of 1 copy.

We also found evidence for the formation of pseudogenes following gene duplication. The amino acid sequences from translations of 1 gene in *H*. *cydno*, the ortholog of *HMEL037104g1*, does not contain a complete functional protein domain. This is also seen for *Herato0606*.*241* in *H*. *erato*. Furthermore, more recent pseudogene formation could be seen in the *H*. *cydno* ortholog of *HMEL22306g3*, which contained multiple stop codons, despite being transcribed (S4 Fig).

We have demonstrated that HmelOS of *H*. *melpomene* acts as an ocimene synthase, with residual IDS activity, while we find no evidence of these functions in its ortholog in *H*. *cydno*, HcydOS. To identify likely functional changes between *H*. *melpomene* and *H*. *cydno* we first analysed the patterns of synonymous and nonsynonymous mutations between these species. The branch leading up to *HMELOS* and *HCYDOS* is under positive selection (ω = 1.33); this ratio is greater than the background ratio of the *Heliconius* GGPPS genes (ω = 0.19) (χ2 = 6.9, *p* = 0.012). However, we find no difference between ω ratio for the *HMELOS* branch and *HCYDOS* branch (χ2 = 0.36, *p* = 0.54).

We then predicted the sequence of the last common ancestor (LCA) of *H*. *melpomene* HmelOS and *H*. *cydno* HcydOS using ancestral sequence reconstruction with a posterior probability of 0.998 (S15 Fig). Six amino acid changes differentiate HmelOS from the LCA, and 3 amino acid changes differentiate HcydO*S* from the LCA. To identify which of these residues are conserved within species, we aligned amino acid sequences from a set of 20 sequences from 10 individuals of each species (S12 Table). Of the sites which differentiate *H*. *cydno* and *H*. *melpomene* from the predicted sequence of the LCA, the most intraspecifically conserved sites occur between amino acid residues 100 and 125, suggesting that this region is likely to be important for the function of the enzyme (S13 Table). All 3 mutations in the *H*. *cydno* lineage (M109T, V119T, and A123T) and 1 of the mutations in the *H*. *melpomene* lineage (R122K) occur in this region. Of the amino acid changes in this region, the mutations in the *H*. *cydno* lineage involve a larger change in amino acid chemistry (from either methionine or small hydrophobic residues to a polar residue) than the mutation between 2 positively charged amino acids in the *H*. *melpomene* lineage.

**Fig 4:**
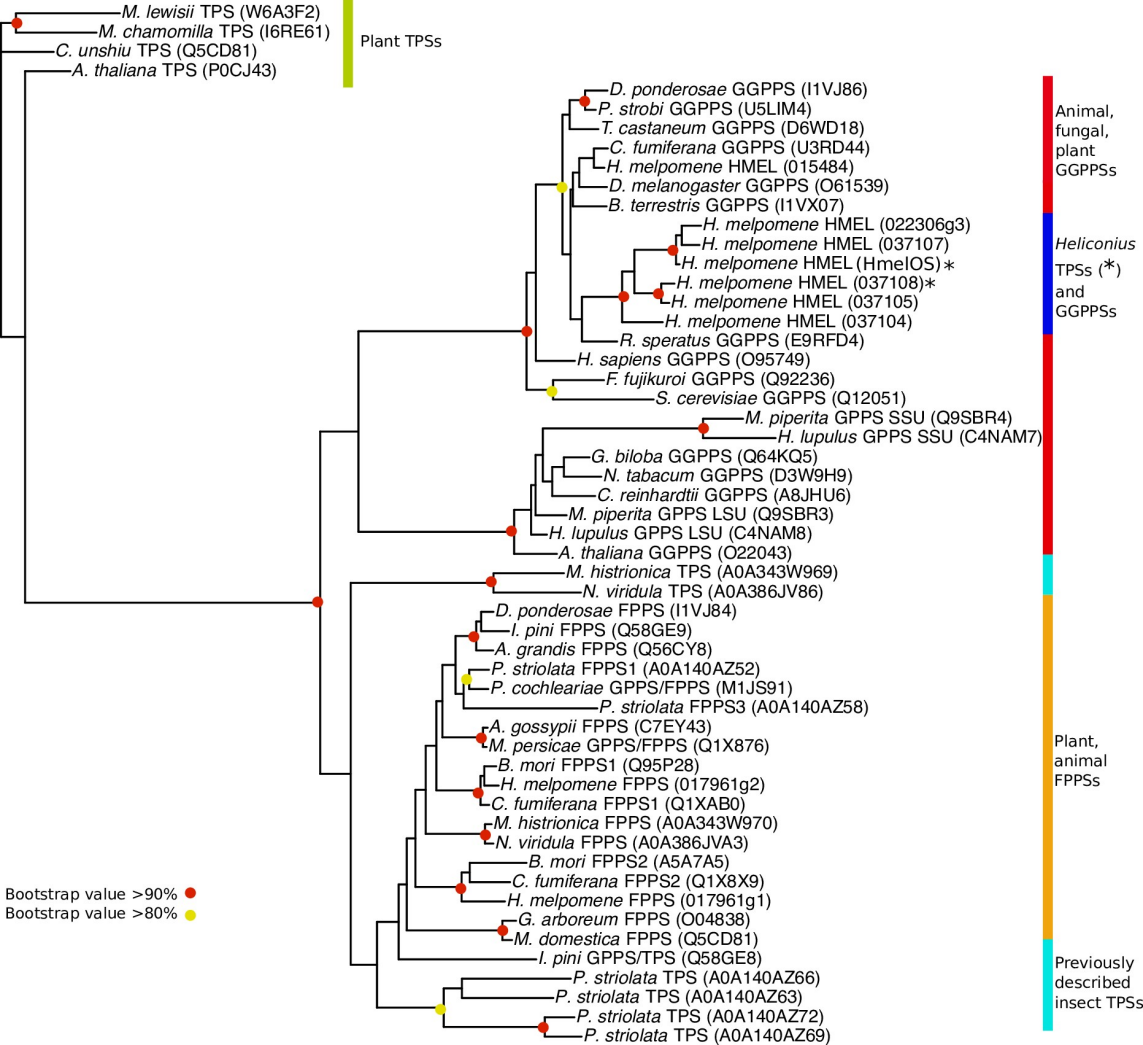
Phylogram of GGPPS, FPPS, and TPS proteins of animals, fungi, and plants. The phylogeny was constructed by PhyML using LG model of amino acid evolution. Bootstrap (*n* = 1000) values are illustrated. The tree was rooted with the ocimene synthase of *Citrus unshiu*. Full species names in S14 Table. Script is available from OSF (https://osf.io/3z9tg/). FPPS, farnesyl diphosphate synthase; GGPPS, geranylgeranyl diphosphate synthase; TPS, terpene synthase.

In order to determine the number of evolutionary origins of insect and plant TPSs, we carried out a broader phylogenetic analysis, including other known insect and plant IDS and TPS proteins. Similar to the other insect TPSs described, *Heliconius* TPSs are not found within the same clade as plant ocimene synthases, representing an independent origin of ocimene synthesis in *Heliconius* and plants. Furthermore, the *Heliconiu*s TPSs do not group with known insect TPS enzymes in Hemiptera and Coleoptera (Fig 4). Instead, the *Heliconius* TPS enzymes group with GPP and GGPP synthases, rather than FPP synthases. The TPS enzymes of *Heliconius* are therefore of an independent evolutionary origin as compared to other insect TPSs.

Comparison of the amino acid alignment of known insect TPSs with the *H*. *melpomene* enzymes (S16 Fig) demonstrated that residues previously identified as conserved in insect TPSs [28] were not found in the *H*. *melpomene* TPSs. No residues were shared between all insect TPSs (including *H*. *melpomene* TPS) which were not also shared with the *H*. *melpomene* GGPPS. This further indicates independent convergent evolution of TPS function in *H*. *melpomene*.

## Discussion

Both plants and animals use terpenes as chemical signals; however, the TPSs that make them have been identified in only a few insect species. Ocimene is a common monoterpene, and we have identified, to our knowledge, the first ocimene synthase in animals. This ocimene synthase is therefore implicated as the locus of divergence controlling differences in ocimene production between closely related species. Within the QTL, we identify 8 genes that have evolved from the repeated duplication of a GGPPS ancestor. We confirm ocimene synthase activity for 1 of these enzymes (HmelOS) and TPS activity in another closely related enzyme (HMEL037108g1). Neither of these *H*. *melpomene* genes are homologous to known plant TPSs. Furthermore, they are also very different from previously described insect TPSs. While the TPS enzymes of Hemiptera and Coleoptera are more closely related to FPPSs [1,27,28], the *H*. *melpomene* TPSs are more closely related to GGPPSs, implying that TPS activity arisen independently in Lepidoptera. At a deeper evolutionary scale, the origin of the (*E*)-β-ocimene synthase activity in *H*. *melpomene* therefore represents an example of chemical convergence via the independent evolution of new gene function. On a more recent timescale, this gene is also responsible for pheromone divergence between the closely related species, *H*. *melpomene* and *H*. *cydno*, perhaps driven by an evolutionary arms race between males and females.

The novel TPS family we identify in *Heliconius* has undergone repeated gene duplication underscoring the importance of duplication in evolution of new functions. In this case, the duplications generated at least 2 loci that possess TPS activity, as well as a number of pseudogenes. This pattern of expansion followed by the evolution of new function, a process called neofunctionalisation [42], often results in large gene families with related but different functions. These families typically follow a birth-and-death model of evolution, expanding and contracting through gene duplication, formation of pseudogenes, and gene deletion [43,44]. Indeed, plant TPSs follow these dynamics, making up a large family formed of 7 subfamilies, with lineage-specific expansions [45,46]. Similarly, a family of TPS genes has also been discovered in flea beetle, *Phyllotreta striolata*, where gene duplication is thought to have enabled functional diversification [27].

Another way in which gene duplication could have facilitated TPS evolution in *Heliconius* is enzyme specialisation through subfunctionalisation. In this case, an ancestrally multifunctional enzyme duplicates, resulting in 2 daughter copies which split the ancestral functions, and can result in optimisation of these 2 functions [47,48]. We propose that subfunctionalisation might explain why neither *Heliconius* TPS shows significant IDS activity. In contrast to the multifunctional TPS/IDS enzyme from *I*. *pini* [25,26], other insects have separate enzymes with IDS and TPS activity [27–29]. Our hypothesis is that an IDS enzyme initially gained TPS activity followed by gene duplication and subfunctionalisation with enzymes specialised for different enzymatic steps. To investigate where the loss of IDS activity occurred, it would be necessary to test more orthologs across the *Heliconius* phylogeny.

Although we describe, to our knowledge, the first ocimene synthase in animals, ocimene synthases are likely to be found in other groups. Both *Bombus terrestris* and *Apis mellifera* use ocimene as a recruitment and larval pheromone, respectively [49,50]. While the biosynthetic pathway is not known in these groups, a similar pathway to that proposed here has been suggested in *A*. *mellifera* [51]. However, the existing data suggest that the loci responsible for ocimene synthesis are also likely to be independently evolved. Unlike *H*. *melpomene*, only 1 GGPPS is found in the *Apis* genome, while there are 6 FPPS genes, the result of lineage-specific duplications [52]. Although this needs to be confirmed by functional studies, based on the genomic patterns, it seems likely that convergence between Lepidoptera and Hymenoptera will also prove to be an independent evolutionary origin of ocimene synthesis.

On a more recent timescale, the locus identified here also control difference in pheromone production between closely related species, offering insights into the genetic basis of trait evolution [53]. Previous studies have identified both coding and regulatory changes as playing a role in the evolution of lepidopteran pheromones [54,55]; here, we combine functional and sequence analyses to show that the loss of (*E*)-β-ocimene production is likely caused by a loss-of-function change in coding sequence. The compound (*E*)-β-ocimene is found in the genitals of multiple *Heliconius* species [14,18] and at least in *H*. *melpomene* is known to act as an anti-aphrodisiac, reducing further mating attempts from other males. The presence of (*E*)-β-ocimene across the lineage [14,18] suggests that its production is ancestral in *Heliconius*. Nonetheless, anti-aphrodiasiac compounds are known to evolve rapidly and not all *Heliconius* species use (*E*)-β-ocimene. In particular, *H*. *melpomene*’s closely related sister species, *H*. *cydno*, does not produce (*E*)-β-ocimene. There are several non-synonymous mutations in the coding region of *HMELOS/HCYDOS* that are likely candidates for causing the loss of (*E*)-β-ocimene production between *H*. *melpomene* and *H*. *cydno*. These differences involve large changes in amino acid chemistry, likely to affect the function of the enzyme. Our functional experiments also support the hypothesis that this enzyme is not capable of producing (*E*)-β-ocimene. Across the genus, *Heliconius* species which produce (*E*)-β-ocimene do not form a monophyletic group, suggesting that this loss of function or change in function may have occurred multiple times in *Heliconius* [14]. Nonetheless, there were also significant differences in abdomen expression levels of *HMELOS/HCYDOS* between *H*. *melpomene* and *H*. *cydno* males, so we cannot rule out an additional role for regulatory change in controlling (*E*)-β-ocimene production. It will be interesting to look more broadly across the genus to see how often the loss of (*E*)-β-ocimene production is the result of functional protein change, expression change, or both.

Although the functional role of HmelOS in the production of (*E*)-β-ocimene is robust, the role of the second TPS enzyme, HMEL037108g1, is less clear. The protein acts as a multifunctional linalool/nerolidol synthase, which have previously been described in plants [56,57]. However, neither linalool nor nerolidol are found in high amounts in the male abdomen [58]. This apparent discrepancy may be due to the location or timing of expression in vivo [27]. Another hypothesis is that in vivo GPP reacts with another substrate in the active site of HMEL037108g1 or that once linalool is produced, it is metabolically channelled to another enzyme for further modification in vivo [59].This could also explain the lack of stereoselectivity in linalool formation. Further experiments will be required to determine if the other enzymes of this family also exhibit TPS activity also.

In summary, we have identified a novel family of TPSs in *Heliconius* butterflies which is unrelated both to plant TPSs and to the few examples of previously described insect TPSs. We confirm that terpene synthesis has multiple independent origins in insects, which are themselves independent from the evolution of terpene synthesis in plants. Furthermore, we suggest that multiple losses of (*E*)-β-ocimene production have occurred throughout the evolution of *Heliconius* butterflies. These examples of convergence at multiple phylogenetic levels provide us with a system to study the predictability of genetic changes underlying phenotypic variation, as well as more specifically how structure relates to function in TPSs. Despite their independent evolution, insect TPSs show significant structural similarities, having evolved from IDS-like proteins. To understand how this diversity has arisen, we need to identify the functional amino acid changes, a nascent area of research for this group of enzymes [60].

## Methods

### Ethics statement

Butterflies were collected under permits SE/AP-23-15 and SE/AP-11-17 issued by the Ministerio de Ambiente de Panamá.

### Analysis of biosynthetic pathway in *H*. *melpomene*

To identify genes involved in terpene biosynthesis, we searched the *H*. *melpomene* genome (v2.5) on LepBase [37,61] for genes in the mevalonate pathway and IDSs. *D*. *melanogaster* protein sequences were obtained from FlyBase and used in BLAST searches (blastp) against all annotated proteins in the *H*. *melpomene* genome (S1 Table) [52,62]. We used the BLAST interface on LepBase with default parameters (-evalue 1.0e-10 -num_alignments 25) [37,63]. We then searched these candidate orthologs against the annotated proteins of *D*. *melanogaster* using the BLAST interface on FlyBase to identify reciprocal best blast hits. We included in our results other hits with an e-value smaller than 1e-^80^.

### Butterfly stocks

Outbred stocks of *H*. *melpomene rosina* and *H*. *cydno chioneus* were established from wild individuals collected in Gamboa (9°7.4′ N, 79°42.2′ W, elevation 60 m) in the nearby Soberania National Park, San Lorenzo National Park (9°17′ N, 79°58′ W; elevation 130 m), and in Altos de Campana National Park (8°69′ N, 79°92′ W; elevation 900 m). Stocks were maintained under ambient conditions in insectaries at the Smithsonian Tropical Research Institute (STRI) facilities in Gamboa, Panama. Individuals for this study were reared between January 2016 and January 2018. Larvae were reared on *Passiflora platyloba*. Adult male butterflies were kept in cages with other males and provided with approximately 20% sucrose solution with access to at least 1 of *Psychotria poeppigiana*, *Gurania eriantha*, *Psiguiria triphylla*, and *Psiguria warscewiczii* as pollen sources.

### Crossing for quantitative trait linkage mapping

To map the genetic basis of ocimene production, we crossed *H*. *melpomene*, which produces (*E*)-β-ocimene, to *H*. *cydno*, a closely related species which does not. We crossed these 2 species to produce F1 offspring and backcross hybrids in both directions. Female F1s are sterile, and so we mated male F1s to *H*. *cydno* and *H*. *melpomene* virgin stock females to create backcross families. Families created by backcrossing to *H*. *melpomene* had a phenotype similar to pure *H*. *melpomene*, suggesting that the *H*. *melpomene* phenotype is dominant. While we used 265 individuals to create the linkage map, we focused on backcross families in the direction of *H*. *cydno*, where the (*E*)-β-ocimene phenotype segregates for the QTL mapping (S1 Fig). We phenotyped and genotyped 114 individuals from 15 backcross families in the direction of *H*. *cydno*. Bodies were stored in dimethyl sulfoxide (DMSO) and stored at −20°C for later DNA extraction.

### Genotyping and linkage map construction

DNA extraction was carried out using Qiagen DNeasy kits (Qiagen, Hilden, Germany). As previously described, individuals were genotyped either by Restriction site Associated DNA sequencing (RAD-seq) [64–66] or low-coverage whole genome sequencing using Nextera-based libraries [66,67]. A secondary purification using magnetic SpeedBeads (Sigma, Gallen, Switzerland) was performed prior to Nextera-based library preparation. Libraries were prepared following a method based on Nextera DNA Library Prep with purified Tn5 transposase [67]. PCR extension with an i7-index primer (N701–N783) and the N501 i5-index primer was performed to barcode the samples. Library purification and size selection were done using the same beads as above. Pooled libraries were sequenced by BGI (China) using HiSeq X Ten (Illumina). Parents were sequenced at an average depth of 30.23 (SD 12.67), grandparents at 12.58 (SD 11.47), and offspring at 9.77 (SD 4.98).

Linkage mapping was conducted following Byers and colleagues [66], using a standard Lep-MAP3 (LM3) pipeline [68]. Briefly, fastq files were mapped to the *H*. *melpomene* reference genome using BWA MEM [69]. Sorted bams were then created using SAMtools, and genotype likelihoods were constructed [70]. The pedigree of individuals was checked and corrected using IBD (identity by descent), and the sex was checked using coverage on the Z chromosomes by SAMtools depth. A random subset of 25% of markers were used for subsequent steps. Linkage groups and marker orders were constructed based on the *H*. *melpomene* genome and checked with grandparental data.

The map constructed contained 447,820 markers. We reduced markers by a factor of 5 evenly across the genome resulting in 89,564 markers with no missing data to facilitate computation. We log-transformed amounts of ocimene produced to conform more closely to normality. Statistical analysis was carried out using R/qtl [101]. We carried out standard interval mapping using the *scanone* function with a nonparametric model, an extension of the Kruskal–Wallis test statistic. The analysis method for this model is similar to Haley–Knott regression [102]. We used permutation testing with 1000 permutations to determine the genome-wide LOD significance threshold. To obtain confidence intervals for QTL peaks, we used the function *bayesint*. Phenotype data, pedigree, linkage map, and R script are available from OSF (https://osf.io/3z9tg/). Sequencing data used for the linkage maps are available from the European Nucleotide Archive (ENA) project PRJEB34160 [66].

### Phenotyping of (*E*)-β-ocimene production

Chemical extractions were carried out on genital tissue of mature (7 to 14 days post-eclosion) male individuals of *H*. *melpomene*, *H*. *cydno*, and hybrids. Genitals were removed using forceps and soaked, immediately after dissection, in 200 μl of dichloromethane containing 200 ng of 2-tetradecyl acetate (internal standard) in 2-ml glass vials with polytetrafluoroethylene-coated caps (Agilent, Santa Clara, California, United States of America). After 1 h, the solvent was transferred to a new vial and stored at −20°C until analysis by GC/MS.

### GC/MS analysis

DMAPP (90%), GPP (95%), and IPP (95%) were purchased from Sigma. FPP (98%) was purchased from VWR (Darmstadt, Germany). (*R*)-Linalool (95%) was purchased from Merck (Darmstadt, Germany). (*S*)-Linalool was isolated from coriander oil (Sigma) and purified through column chromatography.

Extracts from adult butterflies and samples from in vitro experiments were analysed by GC/MS using an Agilent (model 5977/5975) mass-selective detector connected to an Agilent GC (model 7890B/7890A) with electron impact ionisation (70eV). This instrument was equipped with an Agilent ALS 7693 autosampler and an HP-5MS fused silica capillary column (Agilent) (length 30 m, inner diameter 0.25 mm, film thickness 0.25 μm). Injection was performed in splitless mode (injector temperature 250°C) with helium as the carrier gas (constant flow of 1.2 ml/min). The temperature programme started at 50°C for 5 min and then rose at a rate of 5°C/min to 320°C, before being held at 320°C for 5 min. The compounds were identified through comparison with retention time and mass spectra of standard samples.

Chiral analysis of linalool was performed in an Agilent 7820A gas chromatograph equipped with flame ionisation detector (FID) using a chiral column Beta DEX 225 (length 30 m, inner diameter 0.25 mm, film thickness 0.25 μm, Supelco, Bellefonte, Pennsylvania, USA). The oven program started at 50°C for 1 min, followed by increasing the temperature at a rate of 3°C/min until 210°C, keeping this temperature for 5 min. A total of 1 μl of each sample was injected in splitless mode at a flow of 1.65 mL/min. The peak areas were used to calculate the percentage of each stereoisomer.

### *Heliconius cydno* guided assembly and annotation transfer

*H*. *cydno* and *H*. *melpomene* had their most recent common ancestor 1.5 million years ago, and their absolute divergence is roughly 3% (dxy approximately 0.03) [41,71]. Due to this high degree of similarity, it is possible to map *H*. *cydno* RNA-seq reads to the *H*. *melpomene* genome. However, we wanted to accurately quantify gene expression in existing *H*. *cydno* samples (GenBank BioProject PRJNA283415 [34]) by reducing potential biases associated with RNA-seq reads carrying H. *cydno* specific alleles. RNA-seq reads from *H*. *cydno* with such variants have a lower probability to map correctly to the existing *H*. *melpomene* reference. This biases quantification and increases false-positive rates, documented extensively, specifically in the context of allele-specific expression studies [72].

A *H*. *cydno* trio (mother, father, and progeny) was previously Illumina sequenced (ENA study ERP009507) and assembled into maternal and paternal genomes with trio-sga [35]. The paternal genome had 34,566 scaffolds, a total size of 270,339,622 bp and a scaffold N50 of 25,716 bp, with 551 kb of gaps (paternal trio fasta file is available from OSF (https://osf.io/3z9tg/)). To improve gene contiguity, we used the progressiveCactus algorithm (v3) to align the *H*. *cydno* paternal haplotypic assembly to the chromosomal version of the *H*. *melpomene* genome (v2.5) [73–75]. The HAL database created by progressiveCactus was loaded to Ragout (v1.2) [76] to produce the final *H*. *cydno* reference-guided assembly (*H*. *cydno* reference fasta file; ordering information and unplaced scaffolds are available from OSF (https://osf.io/3z9tg/). The *H*. *cydno* guided assembly has 58 scaffolds, a total size of 261,056,210 bp, a scaffold N50 of 13,724,118 bp, and 8.3 Mb of gaps.

We then transferred the *H*. *melpomene* annotation (v2.5) to the *H*. *cydno* assembly. We used EMBOSS Seqret (v6.6.0.0) to convert the *H*. *melpomene* annotation file to the embl format [77], and we used RATT to transfer the *H*. *melpomene* annotation (reference) to the guided *H*. *cydno* genome (query). RATT is part of PAGIT, a post-assembly genome-improvement toolkit (v1.0) [78]. We searched for synteny between the reference and the query using MUMmer (v4.0) and detected possible errors such as start and stop codons or frameshift mutations [79]. After correcting such errors with the RATT pipeline, the annotation transfer to *H*. *cydno* was complete [80].

### Annotation improvements

To ensure that our genes of interest from *H*. *melpomene* (those identified in S1 Table) were correctly annotated, we manually curated these genes in the *H*. *cydno* annotation. To find orthologs in *H*. *cydno*, we used the BLAT function in Apollo to search for *H*. *melpomene* exons [81,82]. We checked the gene models for splice sites and start and stop codons. The curated gene models were then exported from Apollo and manually included in the *H*. *cydno* annotation. We then subset the annotation to include only exons, because CDS sequences had not been properly annotated (Updatedannotation.gff). We then converted it to gtf file format using the gffread function of Cufflinks (Hcyd1.0_annotV2.gtf) [83] and filtered out exons longer than 30,000 bp (Hcyd1.0_annotV2.gtf; gtf_modify_Hcyd_annotV2.R). We finally used the gtf_modify_Hcyd_annotV3.R script to include unique *H*. *cydno* gene-ids (Hcyd1.0_annotV3.gtf).

To check that our genes of interest were correctly annotated in all 3 *Heliconius* species (*H*. *melpomene*, *H*. *cydno*, and *H*. *erato*), we used already published RNA-seq data of abdomens and heads to improve the annotations from GenBank BioProject PRJNA283415 [34]. We performed quality control and low-quality base and adapter trimming on the RNA-seq data using TrimGalore! [84]. We then mapped the reads to the *H*.*melpomene* genome v2.5 [75], our newly assembled *H*.*cydno* genome, and the *H*. *erato* genome [85] using STAR. In all cases, we ran 2pass and set a maximum intron length to 80,350 bp, which is the size of longest intron in the *H*. *melpomene* genome [86]. We used StringTie [87] to assemble transcripts genome wide and compared the new assembly of out candidate genes with the original annotation. *H*. *melpomene* genes *HMEL022305g1 and HMEL037104g1* were merged into 1 new gene (now called *HMEL037104g1* in Hmel2.5_edited.gtf) based on this evidence. Furthermore, genes *HMEL007429g2* and *HMEL007429g3* were also merged (now called *HMEL007429g4* in Hmel2.5_edited.gtf), as the first 2 exons of *HMEL007429g2* and the last exon of *HMEL007429g3* are expressed as a single transcript. We also edited the *H*. *erato* annotation to split *Herato0606*.*239* into *Herato0606*.*239_a* and *Herato0606*.*239_b* (Herato_edited.gff). We made no further changes to the annotation of *H*. *cydno*.

### RNA-sequencing analysis

Gene expression analyses were performed using already published RNA-seq data from heads and abdomens of *H*. *melpomene* and *H*. *cydno* from GenBank BioProject PRJNA283415 [34], the same data used to improve the annotations. We used the reads after the trimming step done with TrimGalore! [84]. We then remapped the reads to the *H*.*melpomene* genome v2.5 [75] and our newly assembled *H*.*cydno* genome using STAR, this time including our manually edited annotations (Hmel2.5_edited.gtf and Hcyd1.0_annotV3.gtf). As before, we ran 2pass and set a maximum intron length to 80,350 bp. *featureCounts* [88] was used to produce read counts that were normalised by library size with trimmed mean of M values (TMM) normalisation [89] using the edgeR package in R [90]. To test for differences in expression of our candidate genes, we used the *voom* function from the limma package in R [91], which fits a linear model for each gene by modelling the mean–variance relationship with precision weights.

To test for male abdomen-biased expression within *H*. *melpomene*, we included 2 fixed effects, sex, and tissue, as well as including individual as a random effect (expression ~ sex + tissue + sex*tissue + (1|individual)). We were looking for genes with a significant interaction between sex and tissue, showing higher expression in male abdomens. To test for differences in expression between *H*. *melpomene* and *H*. *cydno* abdomens, we included 2 fixed effects, sex, and species, as well as an interaction term (expression ~ sex + species + species*sex). We were interested in finding differences in the extent of sex bias between species, again detected by a significant interaction term with higher expression in *H*. *melpomene* male abdomens.

*p*-Values were corrected for multiple testing using the Benjamini–Hochberg procedure for all genes in the genome-wide count matrix (17,902 for *H*. *melpomene*). For the interspecific comparison, we identified genome-wide orthologs from the annotation and produced a gene count matrix including both species. The ortholog list was limited to genes that had only 1 ortholog in each species (11,571 genes). Scripts are available from OSF (https://osf.io/3z9tg/).

### *In vitro* expression and enzymatic assays

RNA extraction from male abdominal tissue of *H*. *melpomene* was carried out following a standard TRIzol protocol (Invitrogen, Glasgow, United Kingdom) and cDNA synthesised using 5x iScript Reaction Mix (Bio-Rad, Basel, Switzerland). DNA for *HCYDOS* was synthesised by Eurofins Genomics. For *HCYDOS*, we used a sequence obtained from whole genome resequencing data as the *H*. *cydno* reference genome had a gap in this region (sequence CJ565_B, S12 Table, Resequenced_mel_cyd.fa). Following the protocol from Champion pET101 Directional TOPO™ Expression Kit (Invitrogen), we amplified the full-length transcript of genes of interest from the cDNA by PCR using Q5 High-Fidelity 2x Master Mix (Biolabs, Ipswich, Massachusetts, USA), with gene-specific primers (S15 Table). The primers were designed for full-length transcript amplification for *H*. *melpomene*. For *H*. *cydno*, the primers amplified all but a 23-bp section at the 3′ end of the gene, the opposite end to the predicted active site. The plasmid contains an in-frame stop codon 26 bp from the end of our inserted *H*. *cydno* sequence. Therefore, transcription is terminated 23 bp from the end of our amplified sequence resulting in a protein which is expected to be of the same length as the true protein, the only difference being that the last 8 amino acids are different. We do not expect this change to affect protein folding.

The PCR products were purified using a MiniElute PCR purification kit (Qiagen) and then sequenced to confirm identity (S15 Table). Following sequencing, the PCR products were ligated into the expression vector pET101/D-TOPO and transformed into One Shot TOP10 Chemically Competent *E*. *coli* cells. Plasmids were extracted from cultures of successful colonies using the QIAprep Spin Miniprep Kit (Qiagen) and sequenced again to confirm correct ligation in the vector using the T7 and T7-reverse primers (S15 Table).

Plasmids containing the genes of interest in the correct orientation were transformed into *E*. *coli* strain BL21 Star(DE3) for expression. Cell cultures were grown to an OD_600_ of 0.5 and induced with 1 mM IPTG. After induction, the cells were cultivated for a further 2 h at 37°C and 250 revolutions per minute (rpm), before collection by centrifugation for 15 min at 6,000xg at 4°C. Expression of protein was verified using sodium dodecyl sulfate polyacrylamide gel electrophoresis (SDS/PAGE) (S5 Fig). Pellets were resuspended in chilled extraction buffer (25 mM 4-(2-hydroxyethyl)-1-piperazineethanesulfonic acid pH 7.5, 1 mM MnCl2, 100 mM KCl, 3 mM dithiothreitol, 10% glycerol, protease inhibitor cocktail (Sigma)) and disrupted by sonication. Cell lysates were then centrifuged for 10 min at 9,000xg at 4°C and the supernatant (containing the soluble part of the cell lysate) retained.

TPS and IDS activity was assayed using the soluble fraction of the cell lysate. Protein concentration was estimated using a Qubit Protein Assay Kit (Invitrogen). A total of 80 to 100 ng of protein was added to each reaction in a total volume of 300 μl. We added different precursors from different steps in the pathway (Fig 1) to characterise enzymatic activity. Experiments were incubated at 30°C for 2 h at 200 rpm.

First, we added DMAPP and IPP (100μM each), the 2 building blocks at the beginning of the pathway. To form a terpene from these compounds, they first need to be combined to form GPP, which can then be converted to a terpene by TPS activity. If the enzyme is a multifunctional GPPS/TPS, as in *I*. *pini*, monoterpenes should be formed from DMAPP and IPP, via the production of GPP. Furthermore, if FPPS or GGPPS activity is present, FPP or GGPP could be formed from DMAPP and IPP, as well as sesquiterpenes or diterpenes if sesquiterpene or diterpene synthase activity is exhibited. We then carried out assays with GPP (100μM) and IPP (50μM). If the enzyme solely exhibits monoterpene synthase activity, the monoterpene could only be formed from GPP directly and not from DMAPP and IPP. Furthermore, the enzyme could be an FPPS or GGPPS and could therefore produce FPP or GGPP from GPP and IPP. FPP and GGPP could be converted to sesquiterpenes or diterpenes if sesquiterpene or diterpene synthase activity is exhibited. We also tested with GPP alone (100 μM) to test for monoterpene synthase activity directly. Finally, we carried out assays with FPP (100 μM) and IPP (50 μM). If the enzyme is a GGPPS as annotated, it should form GGPP from FPP and IPP, as well as potentially converting GGPP to diterpenes. This is also a test for sesquiterpene synthase activity, as sesquiterpenes should be formed from FPP if the enzyme is a sesquiterpene synthase. We also tested for enzymatic activity with (*R*)-linalool and (*S*)-linalool (100 μM).

To test for IDS activity, we repeated the above experiments with DMAPP and IPP, GPP, and IPP, and FPP and IPP, followed by treatment with alkaline phosphatase to hydrolyse the isoprenyl diphosphate products to their respective alcohols. These alcohols can then be detected by GC/MS analysis.

Dephosphorylation of GPP produces the monoterpene alcohol geraniol, while dephosphorylation of FPP produces the sesquiterpene alcohol farnesol. Our expectation for controls, without IDS or TPS enzymatic activity, is to find geraniol when GPP is provided and farnesol when FPP is provided. Linalool is a monoterpene alcohol which is an isomer of geraniol, and nerolidol is a sesquiterpene alcohol which is an isomer of farnesol. Furthermore, geranylgeraniol is a diterpene alcohol derived from the dephosphorylation of GGPP. If an enzyme is exhibiting IDS activity, we expect it to be able to catalyse the condensation of IPP and the other precursor provided, DMAPP, GPP, or FPP, to form larger molecules. Therefore, when provided with DMAPP and IPP, we expect to find either monoterpene or sesquiterpene alcohols, derived from GPP or FPP. When provided with GPP and IPP, we expect sesquiterpene alcohols derived from FPP. When provided with FPP and IPP, we expect larger diterpene alcohols, such as geranylgeraniol, to form via the formation of GGPP.

For TPS activity assays, reactions were stopped on ice and overlaid with 250-μl hexane and left at 25°C overnight. The hexane layer was then transferred to a new vial and stored at −80°C. For IDS activity assays, following incubation with the precursors, 20 units of alkaline phosphatase (Sigma) in alkaline phosphatase buffer was added to each reaction mixture and incubated at 30°C for 4 h at 200 rpm. Following this, 250-μl hexane was added and left at 25°C overnight. The hexane layer was then transferred to a new vial and stored at −80°C. Prior to analysis by GC/MS, 20 μL of a solution of 2-acetoxytetradecane in hexane (10 μg/mL) was added as an internal standard, and samples were concentrated to a volume of approximately 30 μL. Products were compared to control experiments where protein expression was not induced. GC/MS data are available from OSF (https://osf.io/3z9tg/).

### Phylogenetic and selection analyses

To identify orthologs of the GGPPS in other Lepidoptera, we searched protein sequences from *H*. *melpomene* version 2.5 [64,75] against the genomes of *H*. *erato demophoon* (v1), *B*. *anynana* (v1x2), *D*. *plexippus* (v3), *P*. *polytes* (ppol1), *P*. *napi* (pnv1x1), *M*. *sexta* (msex1), *B*. *mori* (asm15162v1), and *P*. *xylostella* (pacbiov1) using the BLAST interface (tblastn) on LepBase [37,63]. We also included the previously identified orthologs from the *H*. *cydno* genome (S1 Data). To check that the predicted orthologs contained functional protein domains, we used the National Center for Biotechnology Information (NCBI) conserved domain search with default parameters [92]. We deleted any proteins found without complete functional domains, including a gene from *H*. *erato*, *Herato0606*.*241*, and the *H*. *cydno* ortholog of *HMEL037104g1*. We also did not include the *H*. *cydno* ortholog of *HMEL22306g3*, as despite showing transcription (S4 Fig), there were multiple stop codons within the coding region. For *HCYDOS*, we used a sequence obtained from whole genome resequencing data as the *H*. *cydno* reference genome had a gap in this region (sequence CJ565_B, S12 Table, Resequenced_mel_cyd.fa). We used this sequence for all functional assays, phylogenetic and selection analyses, and ancestral state reconstruction.

To focus on the *Heliconius*-specific duplications, we downloaded the transcript sequences for the *H*. *melpomene* and *H*. *erato* proteins from LepBase and exported transcripts for predicted genes in Apollo for *H*. *cydno*. (S1 Data). We used gene *Herato0606*.*245* (GGPPS, shows high similarity to the GGPPS of the moth *C*. *fumiferana*) to root the tree.

To investigate the evolutionary relationship of the *Heliconius* GGPPS, we carried out a broader phylogenetic analysis with other known insect and plant IDS and TPS proteins. Protein sequences for these additional enzymes were downloaded from UniProt [93]. *Heliconius* protein sequences were obtained as described above. We used an ocimene synthase enzyme from *Citrus unshiu* to root the tree.

We aligned amino acid sequences using Clustal Omega on the EMBL-EBI interface [94]. Both sets of nucleotide sequences were aligned in MASCE using default parameters (gap creation: -7, gap extension: -1, frameshift cost: -30, stop codon not at the end of sequence: -100) [95]. Alignments were visualised using BoxShade (https://embnet.vital-it.ch/software/BOX_form.html). Phylogenetic trees based were constructed in PhyML using the model LG for amino acid sequences and GTR + G + I for nucleotide sequences [96]. These phylogenies were plotted using the package *ape* and *evobiR* in R version 3.5.2. [97–99]. Phylogenies and R code are available from OSF (https://osf.io/3z9tg/).

We performed selection analysis on the set of 34 Lepidoptera GGPPS sequences and the set of 17 *Heliconius* GGPPS sequences. To compare the selection pressure on *Heliconius* and *Bicyclus* with other lepidopteran GGPPS gene lineages which have not undergone extensive expansion, we used codon substitution models implemented in Phylogenetic Analysis by Maximum Likelihood (PAML) [100]. We constructed models under 2 assumptions: The first assumed 1 ω ratio (the ratio of nonsynonymous substitutions (dN) to synonymous substitutions (dS)) for the whole tree, and the second assumed 2 ω ratios, 1 for *Heliconius* and *Bicyclus* and the second for the rest of the tree. The models were compared using a likelihood ratio test (LRT).

Similarly, to analyse the selection pressures on *HMELOS* and *HCYDOS*, we implemented codon substitution models in PAML using the *Heliconius* gene tree. We constructed models under 5 assumptions: The first assumed 1 ratio for the whole tree, the remaining models assumed 2 ω ratios, the first ω ratio for either the *HMELOS* branch, the *HCYDOS* branch, the branch leading up to *HMELOS* and *HCYDOS* or the *HCYDOS* and *HMELOS* branch varying together under a single ratio and the second ω ratio for the rest of the tree. To test whether the *HMELOS*, *HCYDOS*, or the branch leading up to these genes differs from the background ratio, each 2-ratio model was compared to the 1-ratio model using LRTs. To test whether the ω ratio differs between the *HCYDOS* and *HMELOS* branches, the 2-ratio models where the respective branches were allowed to vary were compared using LRT, and both models were compared to a third model where both the *HCYDOS* and *HMELOS* were allowed to vary under the same ratio.

### Ancestral sequence reconstruction

The amino acid sequence of the LCA of HcydOS and HmelOS was calculated using the aaml program in PAML. The set of 17 *Heliconius* sequences used in the phylogenetic analysis was manually refined to minimise gaps in the multiple sequence alignment (MSA) (Heliconius_ASR_edited_aligned). This is because there are no models for insertions and deletions in PAML programs. The manually refined *Heliconius* sequences were aligned using Clustal Omega on the EMBL-EBI interface with default parameters [94]. A phylogenetic tree for these *Heliconius* sequences was generated in PhyML with 1,000 bootstraps; the best model was JTT + G + I + F. Marginal ancestral sequence reconstruction was performed using the aaml program in PAML using an empirical model and wag.DAT substitution rate matrix. To investigate intraspecific variation in the presence of these amino acid differences, we aligned sequences from whole genome sequencing of 10 individuals of *H*. *melpomene* and 10 *H*. *cydno* from Panama (S12 Table, Resequenced_mel_cyd.fa). Two sequences were generated per individual, not representing true haplotypes. We aligned amino acid sequences using Clustal Omega on the EMBL-EBI interface [94]. Alignments were visualised using BoxShade (https://embnet.vital-it.ch/software/BOX_form.html). Manually curated sequences and gene sequences from resequenced individuals are available from OSF (https://osf.io/3z9tg/).

## Supporting information

S1 Fig Amount of (*E*)-β-ocimene (ng) in both pure parental species, F1 hybrids, and backcrosses in both directions. The phenotype segregates in backcrosses to *H*. *cydno* and, therefore, we focused on this cross direction. Raw data and scripts are available from OSF (https://osf.io/3z9tg/).
(PDF)

S2 Fig Effect plot for QTL peak on chromosome 6. Log amount of (*E*)-β-ocimene produced by each genotype at the marker with the highest LOD score. Individuals homozygous for *H*. *cydno* alleles produce less (*E*)-β-ocimene than heterozygotes with a *H*. *melpomene* allele. Sequencing data used to make linkage maps are available from ENA study PRJEB34160. Raw data and scripts are available from OSF (https://osf.io/3z9tg/). ENA, European Nucleotide Archive; LOD, log odds ratio; QTL, quantitative trait locus.
(PDF)

S3 Fig Log2-expression of the candidate genes *H*. *melpomene* heads and abdomens of males and females. *HMELOS* (highlighted) shows male abdomen-biased expression. Full model statistics in S2 Table. *N* = 5 for each boxplot. Gene expression is given in log2 of normalised counts per million (using the TMM). RNA-seq data of *H*. *cydno* and *H*. *melpomene* heads and abdomens were obtained from GenBank BioProject PRJNA283415. Processed data and scripts are available from OSF (https://osf.io/3z9tg/). RNA-seq, RNA sequencing; TMM, trimmed mean of M values.
(PNG)

S4 Fig Log2-expression of the candidate genes in *H*. *melpomene* and *H*. *cydno* abdomens in males and females. Both *HMELOS* and *HMEL037108g1* (highlighted in bold) show greater male-biased expression in *H*. *melpomene* than *H*. *cydno*. Full model statistics in S3 Table. *N* = 5 for each boxplot. Gene expression is given in the log2 of the normalised counts per million using TMM normalisation. RNA-seq data of *H*. *cydno* and *H*. *melpomene* heads and abdomens was obtained from GenBank BioProject PRJNA283415. Processed data and scripts are available from OSF (https://osf.io/3z9tg/). RNA-seq, RNA sequencing; TMM, trimmed mean of M values.
(PNG)

S5 Fig SDS/PAGE gels showing expression of HmelOS and HcydOS. Gels show expression of (A) HmelOS from *H*. *melpomene* and HcydOS from *H*. *cydno* and (B) HcydOS from *H*. *cydno* and an empty vector control. For each, protein expression was tested under different conditions. Firstly, at a 0-h time point (0 h), secondly, at a 2-h time point but with no induction of protein expression (U), and thirdly, at a 2-h time point with protein expression induced (I). The band of interest (indicated by the arrow) is only present under the induced conditions at a 2-h time point and is present in both species but not the empty vector. Ladder is kilodaltons. Raw gel images are available from OSF (https://osf.io/3z9tg/). SDS/PAGE, sodium dodecyl sulfate polyacrylamide gel electrophoresis.
(PDF)

S6 Fig Control experiments for TPS activity acitivites. Control experiments (protein expression uninduced) for the functional characterisation of TPS activity of (A) HmelOS, (B) HcydOS, and (C) HMEL037108g1 from *H*. *melpomene*. Total ion chromatograms of products in the presence of different precursor compounds. (*E*)-β-Ocimene is not produced in any treatments. Linalool and geraniol are produced in small amounts in both, likely due to endogenous bacterial activity. 1, Linalool; 2, Geraniol; 3, Farnesol; *, contaminant from medium; IS, internal standard. Abundance is scaled to the highest peak of all panels per enzyme. Quantification of peaks in S4–S6 Tables. Raw GC/MS data are available from OSF (https://osf.io/3z9tg/). GC/MS, gas chromatography/mass spectrometry; TPS, terpene synthase.
(PDF)

S7 Fig Experiments demonstrating that linalool is not metabolised into ocimene by HmelOS. (A) Total ion chromatograms of enzymatic products in the presence of different linalool stereoisomers. No enzymatic activity is detected. (B) Total ion chromatograms of control experiments (protein expression not induced) in the presence of different Linalool stereoisomers. Again, as expected, no enzymatic activity is detected. 1, Linalool; *, contaminants from medium; IS, internal standard. Abundance is scaled to the highest peak of all panels. Quantification of peaks in S7 Table. Raw GC/MS data are available from OSF (https://osf.io/3z9tg/). GC/MS, gas chromatography/mass spectrometry.
(PDF)

S8 Fig Chiral analysis of linalool produced by HmelOS and HMEL037108g1. (A) Linalool produced in experiments with HmelOS is mainly (S)-linalool (ratio 97:3, S:R), (B) linalool produced in experiments with HMEL037108g1 is a racemic mixture (ratio 54:56, S:R), (C) (R)-linalool, (D) (S)-linalool, (E) Racemic linalool mixture. Raw GC/MS data are available from OSF (https://osf.io/3z9tg/). GC/MS, gas chromatography/mass spectrometry.
(PDF)

S9 Fig Functional characterisation of IDS activity of HmelOS. (A) Total ion chromatograms of enzymatic products in the presence of different precursor compounds, following treatment by alkaline phosphatase. GPP is dephosphorylated to produce geraniol, and FPP to produce farnesol, demonstrating that the main function of HmelOS is not as an IDS. (B) Total ion chromatograms of control experiments (protein expression not induced) in the presence of different precursor compounds, following treatment by alkaline phosphatase. As expected, GPP is dephosphorylated to geraniol, and FPP to farnesol. 1, (*E*)-β-Ocimene; 2, Linalool; 3, Geraniol; 4, Farnesol; *, contaminant from medium; IS, internal standard. Abundance is scaled to the highest peak of all panels. Quantification of peaks in S9 Table. Raw GC/MS data are available from OSF (https://osf.io/3z9tg/). FPP, farnesyl diphosphate; GC/MS, gas chromatography/mass spectrometry; GPP, geranyl diphosphate; IDS, isoprenyl diphosphate synthase.
(PDF)

S10 Fig Functional characterisation of IDS activity of HMEL037108g1 from *H*. *melpomene*. (A) Total ion chromatograms of enzymatic products in the presence of different precursor compounds, following treatment by alkaline phosphatase. As in Fig 3, GPP is converted to linalool and FPP to nerolidol, with remaining GPP dephosphorylated to geraniol, and FPP to farnesol. HMEL037108g1 is acting as a mono- and sesquiterpene synthase, not an IDS. (B) Total ion chromatograms of control experiments (protein expression not induced) in the presence of different precursor compounds, following treatment by alkaline phosphatase. GPP is dephosphorylated to geraniol and FPP to farnesol. 1, Linalool; 2, Geraniol; 3, Nerolidol; 4, Farnesol; *, contaminant from medium; IS, internal standard. Abundance is scaled to the highest peak of all panels. Quantification of peaks in S10 Table. Raw GC/MS data are available from OSF (https://osf.io/3z9tg/). FPP, farnesyl diphosphate; GC/MS, gas chromatography/mass spectrometry; GPP, geranyl diphosphate; IDS, isoprenyl diphosphate synthase.
(PDF)

S11 Fig Functional characterisation of IDS activity of HcydOS. (A) Total ion chromatograms in the presence of different precursor compounds, following treatment by alkaline phosphatase. GPP is dephosphorylated to produce geraniol, and FPP to produce farnesol, demonstrating that the HcydOS is not an IDS. (B) Total ion chromatograms of control experiments (protein expression not induced) in the presence of different precursor compounds, following treatment by alkaline phosphatase. As expected, GPP is dephosphorylated to geraniol, and FPP to farnesol. 1, Geraniol; 2, Farnesol; *, contaminant from medium; IS, internal standard. Abundance is scaled to the highest peak of all panels. Quantification of peaks in S11 Table. Raw GC/MS data are available from OSF (https://osf.io/3z9tg/). FPP, farnesyl diphosphate; GC/MS, gas chromatography/mass spectrometry; GPP, geranyl diphosphate; IDS, isoprenyl diphosphate synthase.
(PDF)

S12 Fig Chemical standards used in experiments. Chromatograms (A) and mass spectra (B) of standards. Nerolidol is a mix of stereoisomers. Raw GC/MS data are available from OSF (https://osf.io/3z9tg/). GC/MS, gas chromatography/mass spectrometry.
(PDF)

S13 Fig Unrooted phylogenetic tree showing the relationship between lepidopteran GGPPS nucleotide sequences. The clades which have undergone lineage-specific expansion of the GGPPS families, *Heliconius* and *Bicyclus*, are shaded red and blue, respectively. The phylogeny was constructed in PhyML using the model GTR + G + I. Bootstrap values (*n* = 1,000) are illustrated. Script is available from OSF (https://osf.io/3z9tg/). GGPPS, geranylgeranyl diphosphate synthase.
(PDF)

S14 Fig Phylogram of genes annotated as GGPPSs in *Heliconius melpomene*, *H*. *cydno*, *and H*. *erato*. These include *HMELOS* and *HMEL037108g1* (*) which encode TPSs. The phylogeny was constructed in PhyML using the model GTR + G + I. Bootstrap values (*n* = 1,000) are illustrated. The *H*. *erato* gene *Herato0606.245* (GGPPS, shows high similarity to the GGPPS of the moth *Choristoneura fumiferana*) was used to root the tree. Script is available from OSF (https://osf.io/3z9tg/). GGPPS, geranylgeranyl diphosphate synthase; TPS, terpene synthase.
(PDF)

S15 Fig Amino acid alignment of the *H*. *melpomene* TPS, HmelOS, HcydOS, and a reconstructed sequence of this protein in the *H*. *melpomene*/*H*. *cydno* LCA. All amino acid substitutions occurred at ancestral protein sites constructed with high posterior probability (>0.9), except 1 substitution between the ancestral sequence and HcydOS (M109T) which occurred at a site with a posterior probability >0.8. Amino acid sites shared in all 3 sequences are shaded black. Polymorphic sites are shaded white or grey, with grey shading indicating a substitution to an amino acid with similar chemical properties as calculated in the BOXSHADE software (https://embnet.vital-it.ch/software/BOX_form.html). LCA, last common ancestor; TPS, terpene synthase.
(PNG)

S16 Fig Amino acid alignment between *H*. *melpomene* GGPPS and TPSs and other insect TPSs. The 2 aspartate-rich motifs are labelled FARM and SARM. Stars show residues identified as conserved between insect TPSs [28]. Hmel, *H*. *melpomene*; Mhistr, *Murgantia histrionica*; Ip, *Ips pini*; Pstri, *Phyllotreta striolata*; Hmel_TPS_1, HmelOS; Hmel_TPS_2, HMEL037108g1. Raw data and results files are available from OSF (https://osf.io/3z9tg/). GGPPS, geranylgeranyl diphosphate synthase; TPS, terpene synthase.
(PDF)

S1 Table *Drosophila melanogaster* query protein sequences. Sequences were downloaded from FlyBase and searched (blastp) against all annotated proteins in the genome of *Heliconius melpomene* (v2.5) on LepBase to identify homologs of enzymes involved in the mevalonate and putative terpene synthesis pathways. The candidate orthologs identified in *H*. *melpomene* were then searched (blastp) against annotated proteins in the *D*. *melanogaster* genome on FlyBase. Reciprocal best blasts are highlighted in bold. We included other hits with an e-value smaller than 1e^-80^.
(DOCX)

S2 Table Linear model statistics for differential gene expression analysis in *H*. *melpomene* heads and abdomens of both sexes. The model includes 2 fixed terms, tissue and sex, their interaction, and a random term, individual (expression ~ sex + tissue + sex*tissue + (1|individual). The Log FC column gives the log2 Fold Change between the groups being compared, while the Ave. Expr. column gives the mean log2-exprtession across all samples. Column t is the moderated t-statistic, and B is the B-statistic; the log odds that the gene is differentially expressed. The Adj. *p*-value column gives *p*-values (bold are significant) corrected for multiple testing using the Benjamini and Hochberg’s method to control the false discovery rate across all tested genes (17,902). RNA-seq data of *H*. *cydno* and *H*. *melpomene* heads and abdomens was obtained from GenBank BioProject PRJNA283415. Processed data and scripts are available from OSF (https://osf.io/3z9tg/). RNA-seq, RNA sequencing.
(DOCX)

S3 Table Linear model statistics for differential gene expression analysis in *H*. *melpomene* and *H*. *cydno* abdomens of both sexes. The model includes 2 fixed terms, species and sex, and their interaction (expression ~ sex + species + species*tissue). The Log FC column gives the log2 Fold Change between the groups being compared, while the Ave. Expr. column gives the mean log2-exprtession across all samples. Column t is the moderated t-statistic, and B is the B-statistic; the log odds that the gene is differentially expressed. The Adj. *p*-value column gives *p*-values (bold are significant) corrected for multiple testing using the Benjamini and Hochberg’s method to control the false discovery rate across all tested genes (11,571). RNA-seq data of *H*. *cydno* and *H*. *melpomene* heads and abdomens was obtained from GenBank BioProject PRJNA283415. Processed data and scripts are available from OSF (https://osf.io/3z9tg/). RNA-seq, RNA sequencing.
(DOCX)

S4 Table Quantification of experiments (Fig 3A and S6 Fig) characterising TPS activity of HmelOS. HmelOS is a monoterpene synthase, catalysing the formation of (*E*)-β-ocimene from GPP. Residual IDS activity is shown by the production of (*E*)-β-ocimene, linalool, and nerolidol from DMAPP and IPP. Mean amounts (ng) ± standard deviation for each compound across 3 replicates are shown. (control) indicates experiments where protein expression was not induced. *N* = 3 for each treatment. Raw GC/MS data and quantification of each sample are available from OSF (https://osf.io/3z9tg/). DMAPP, dimethylallyl diphosphate; GC/MS, gas chromatography/mass spectrometry; GPP, geranyl diphosphate; IDS, isoprenyl diphosphate synthase; IPP, isopentenyl diphosphate; TPS, terpene synthase.
(DOCX)

S5 Table Quantification of experiments (Fig 3A and S6 Fig) characterising TPS activity of HMEL037108g1. HMEL037108g1 acts as a mono- and sesquiterpene synthase, producing linalool from GPP and nerolidol from FPP. Small amounts of linalool and nerolidol detected in DMAPP and IPP treatment, and of nerolidol in the GPP treatment, demonstrate residual IDS activity. Mean amounts (ng) ± standard deviation for each compound across 3 replicates are shown. *N* = 3 for each treatment. Raw GC/MS data and quantification of each sample are available from OSF (https://osf.io/3z9tg/). DMAPP, dimethylallyl diphosphate; FPP, farnesyl diphosphate; GC/MS, gas chromatography/mass spectrometry; GPP, geranyl diphosphate; IPP, isopentenyl diphosphate; TPS, terpene synthase.
(DOCX)

S6 Table Quantification of experiments (Fig 3A and S6 Fig) characterising TPS activity of HcydOS. We find no evidence for TPS activity. Mean amounts (ng) ± standard deviation for each compound across 3 replicates are shown. *N* = 3 for each treatment. Raw GC/MS data and quantification of each sample are available from OSF (https://osf.io/3z9tg/). GC/MS, gas chromatography/mass spectrometry; TPS, terpene synthase.
(DOCX)

S7 Table Quantification of experiments (S7 Fig) testing for HmelOS enzymatic activity with linalool. HmelOS does not show enzymatic activity with linalool, demonstrating it is not an intermediate in the synthesis of (*E*)-β-ocimene. Mean amounts (ng) ± standard deviation for each compound across 3 replicates are shown. *N* = 3 for each treatment. Raw GC/MS data and quantification of each sample available from OSF (https://osf.io/3z9tg/). GC/MS, gas chromatography/mass spectrometry.
(DOCX)

S8 Table Summary of products from enzymatic assays. Assays use precursors from different steps in the pathway (Fig 1) with HmelOS and HMEL037108g1 as well as HcydOS (Fig 3A and S4–S6 Tables). “None” stated if no compounds were detected in experimental treatments that were not also found in control treatments.
(DOCX)

S9 Table Quantification of experiments (S9 Fig) characterising IDS activity of HmelOS. Only residual IDS activity is detected, with small amounts of (*E*)-β-ocimene, linalool, and nerolidol produced from DMAPP and IPP. No other IDS activity is detected. High amounts of geraniol and farnesol in both experimental and control treatments is due to dephosphorylation of GPP and FPP, respectively. The main function of HmelOS is the production of (*E*)-β-ocimene from GPP. Mean amounts (ng) ± standard deviation for each compound across 3 replicates are shown. *N* = 3 for each treatment. Raw GC/MS data and quantification of each sample are available from OSF (https://osf.io/3z9tg/). DMAPP, dimethylallyl diphosphate; FPP, farnesyl diphosphate; GC/MS, gas chromatography/mass spectrometry; GPP, geranyl diphosphate; IDS, isoprenyl diphosphate synthase; IPP, isopentenyl diphosphate.
(DOCX)

S10 Table Quantification of experiments (S10 Fig) characterising IDS activity of HMEL037108g1. TPS activity is again demonstrated by the production of linalool from FPP, and nerolidol from FPP. Only residual IDS activity is detected, by the presence of linalool and nerolidol in treatments with DMAPP and IPP, and nerolidol in the GPP treatment. Geraniol and farnesol are present due to dephosphorylation of remaining GPP and FPP in treatments. Mean amounts (ng) ± standard deviation for each compound across 3 replicates are shown. *N* = 3 for each treatment. Raw GC/MS data and quantification of each sample are available from OSF (https://osf.io/3z9tg/). DMAPP, dimethylallyl diphosphate; FPP, farnesyl diphosphate; GC/MS, gas chromatography/mass spectrometry; IDS, isoprenyl diphosphate synthase; IPP, isopentenyl diphosphate; TPS, terpene synthase.
(DOCX)

S11 Table Quantification of experiments (S11 Fig) characterising IDS activity of HcydOS. We find no evidence for IDS activity. Geraniol and farnesol are present due to dephosphorylation of remaining GPP and FPP in treatments. Mean amounts (ng) ± standard deviation for each compound across 3 replicates are shown. *N* = 3 for each treatment, apart from the FPP + IPP (control) which has *N* = 2. Raw GC/MS data and quantification of each sample are available from OSF (https://osf.io/3z9tg/). FPP, farnesyl diphosphate; GC/MS, gas chromatography/mass spectrometry; GPP, geranyl diphosphate; IDS, isoprenyl diphosphate synthase; IPP, isopentenyl diphosphate.
(DOCX)

S12 Table Genome sequencing sample information. Whole genome sequencing samples of *H*. *melpomene* (MEL) and *H*. *cydno* (CYD) from which gene sequences were used for amino acid alignment (Resequenced_mel_cyd.fa). Sequences are available from OSF (https://osf.io/3z9tg/).
(DOCX)

S13 Table Comparison of amino acid differences. The predicted LCA sequence, as well as the HmelOS and HcydOS sequences used for ASR are shown for comparison. The amino acids at these sites are shown for a further 20 sequences from 10 individuals of each species (S12 Table, Resequenced_mel_cyd.fa). All totals do not add up to 20 due to incomplete sequencing reads. Sequences are available from OSF (https://osf.io/3z9tg/). ASR, ancestral state reconstruction; LCA, last common ancestor.
(DOCX)

S14 Table Full names of species from Fig 4 in the main text. (DOCX)

S15 Table Primer sequences. The DNA sequence “CACC” was added to the 5′ end of the forward primer so that it was compatible with the plasmid vector.
(DOCX)

S1 Data Sequences of the orthologs identified in *H*. *cydno*. (XLSX)

## Abbreviations

BLAST: basic local alignment search tool
DMAPP: dimethylallyl diphosphate
DMSO: dimethyl sulfoxide
DPPS: decaprenyl diphosphate synthase
ENA: European Nucleotide Archive
FID: flame ionisation detector
FPP: farnesyl diphosphate
FPPS: farnesyl diphosphate synthase
GC/MS: gas chromatography/mass spectrometry
GGPPS: geranylgeranyl diphosphate synthase
GPP: geranyl diphosphate
IDS: isoprenyl diphosphate synthase
IPP: isopentenyl diphosphate
LCA: last common ancestor
LM3: Lep-MAP3
LRT: likelihood ratio test
MSA: multiple sequence alignment
NCBI: National Center for Biotechnology Information
PAML: Phylogenetic Analysis by Maximum Likelihood
QTL: quantitative trait locus
RAD-seq: Restriction site Associated DNA sequencing
RNA-seq: RNA sequencing
rpm: revolutions per minute
SDS/PAGE: sodium dodecyl sulfate polyacrylamide gel electrophoresis
STRI: Smithsonian Tropical Research Institute
TMM: trimmed mean of M values
TPS: terpene synthase
