# Supplementary figures and images for: A novel terpene synthase controls differences in anti-aphrodisiac pheromone production between closely related Heliconius butterflies
Source: PLoS Biol. 2021 Jan 19;19(1):e3001022. doi: 10.1371/journal.pbio.3001022 (PMC7815096; doi:10.1371/journal.pbio.3001022)

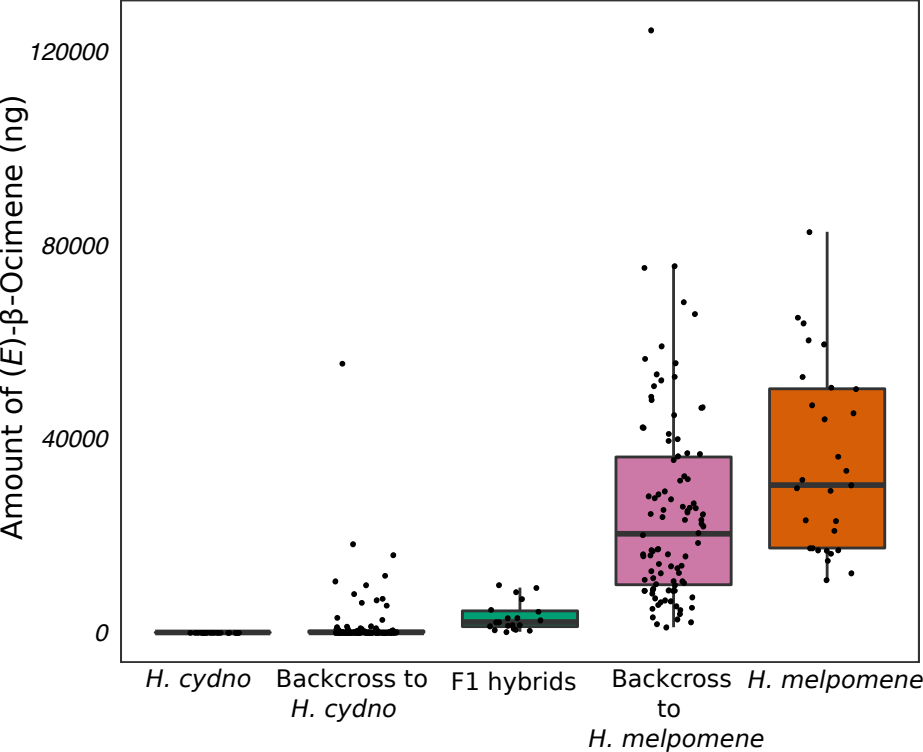

Supplement: S1 Fig — The phenotype segregates in backcrosses to H. cydno and, therefore, we focused on this cross direction. Raw data and scripts are available from OSF (https://osf.io/3z9tg/). (PDF) [file pbio.3001022.s001.pdf]

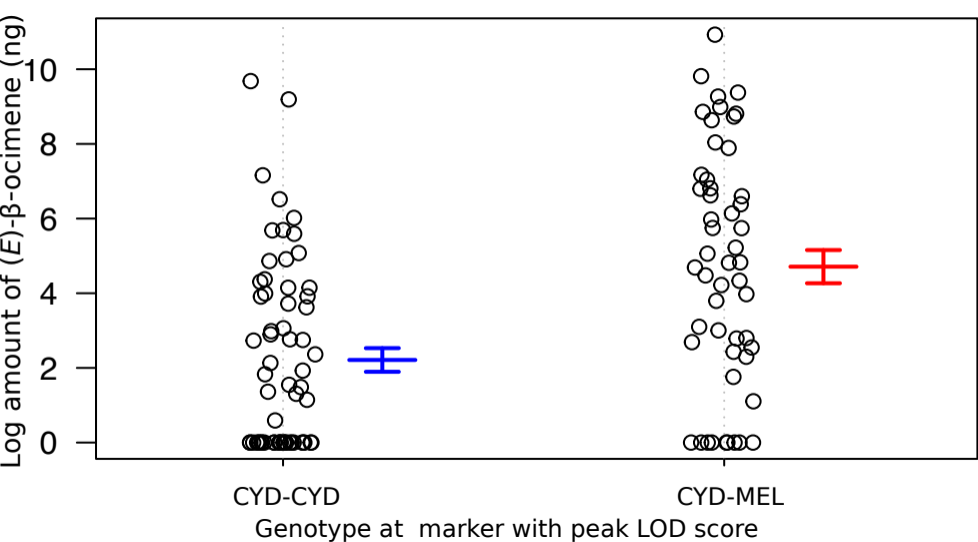

Supplement: S2 Fig — Log amount of (E)-β-ocimene produced by each genotype at the marker with the highest LOD score. Individuals homozygous for H. cydno alleles produce less (E)-β-ocimene than heterozygotes with a H. melpomene allele. Sequencing data used to make linkage maps are available from ENA study PRJEB34160. Raw data and scripts are available from OSF (https://osf.io/3z9tg/). ENA, European Nucleotide Archive; LOD, log odds ratio; QTL, quantitative trait locus. (PDF) [file pbio.3001022.s002.pdf]

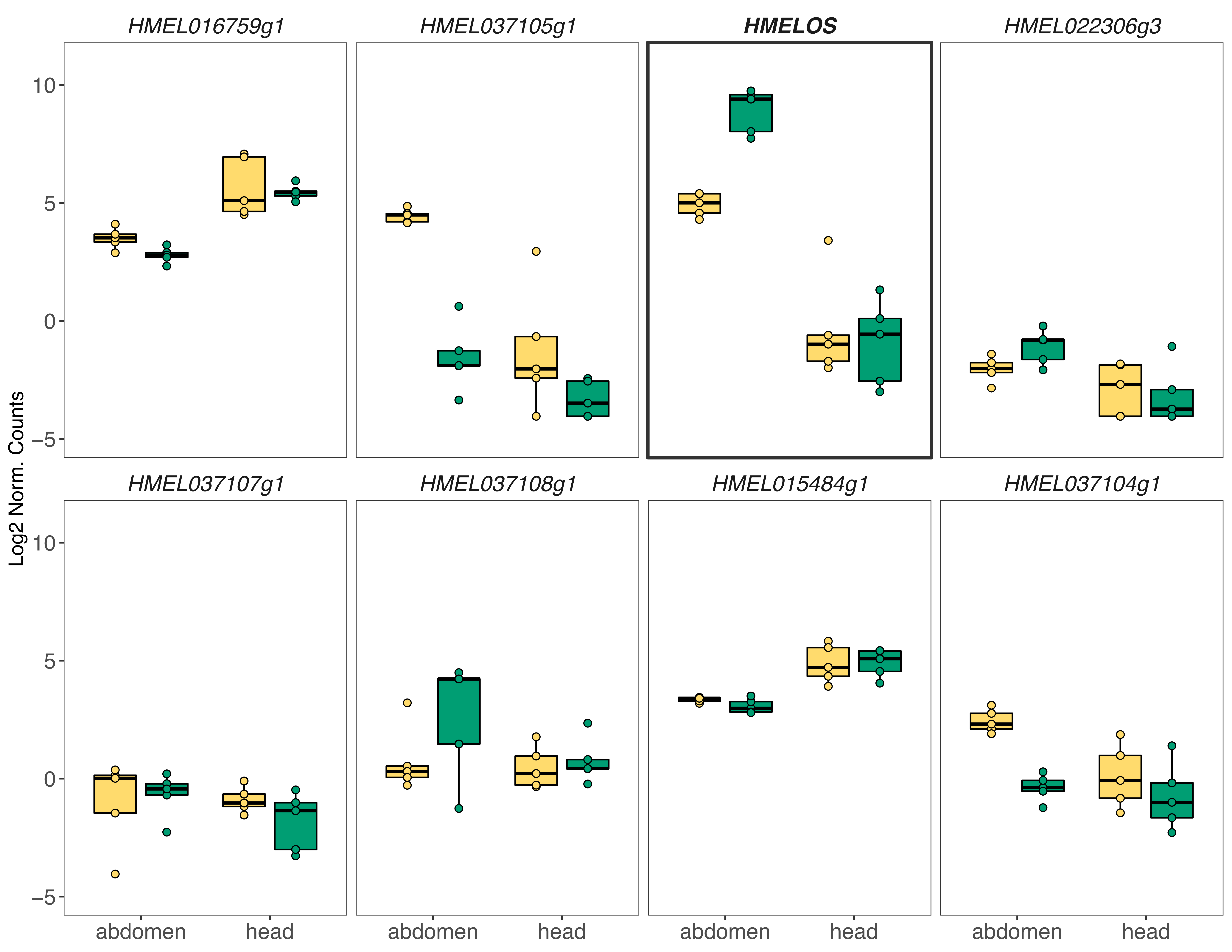

Supplement: S3 Fig — HMELOS (highlighted) shows male abdomen-biased expression. Full model statistics in S2 Table. N = 5 for each boxplot. Gene expression is given in log2 of normalised counts per million (using the TMM). RNA-seq data of H. cydno and H. melpomene heads and abdomens were obtained from GenBank BioProject PRJNA283415. Processed data and scripts are available from OSF (https://osf.io/3z9tg/). RNA-seq, RNA sequencing; TMM, trimmed mean of M values. (PNG) [file pbio.3001022.s003.png]

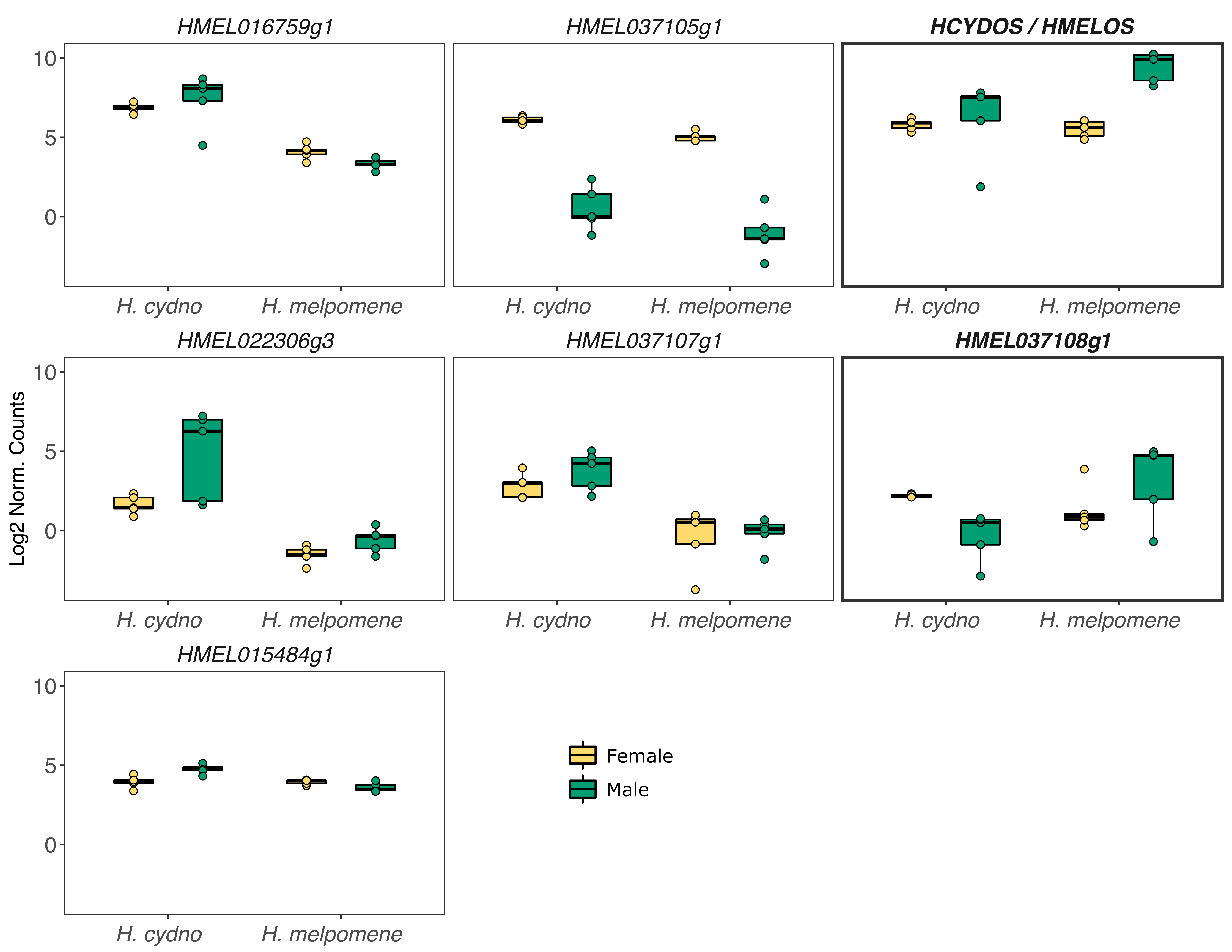

Supplement: S4 Fig — Both HMELOS and HMEL037108g1 (highlighted in bold) show greater male-biased expression in H. melpomene than H. cydno. Full model statistics in S3 Table. N = 5 for each boxplot. Gene expression is given in the log2 of the normalised counts per million using TMM normalisation. RNA-seq data of H. cydno and H. melpomene heads and abdomens was obtained from GenBank BioProject PRJNA283415. Processed data and scripts are available from OSF (https://osf.io/3z9tg/). RNA-seq, RNA sequencing; TMM, trimmed mean of M values. (PNG) [file pbio.3001022.s004.png]

**A**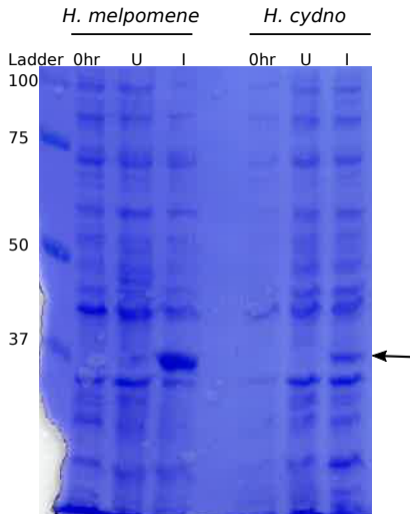**B**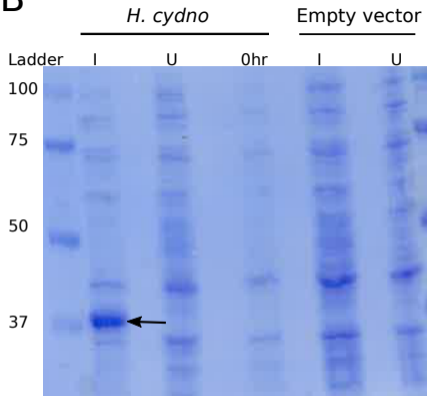

Supplement: S5 Fig — Gels show expression of (A) HmelOS from H. melpomene and HcydOS from H. cydno and (B) HcydOS from H. cydno and an empty vector control. For each, protein expression was tested under different conditions. Firstly, at a 0-h time point (0 h), secondly, at a 2-h time point but with no induction of protein expression (U), and thirdly, at a 2-h time point with protein expression induced (I). The band of interest (indicated by the arrow) is only present under the induced conditions at a 2-h time point and is present in both species but not the empty vector. Ladder is kilodaltons. Raw gel images are available from OSF (https://osf.io/3z9tg/). SDS/PAGE, sodium dodecyl sulfate polyacrylamide gel electrophoresis. (PDF) [file pbio.3001022.s005.pdf]

**A**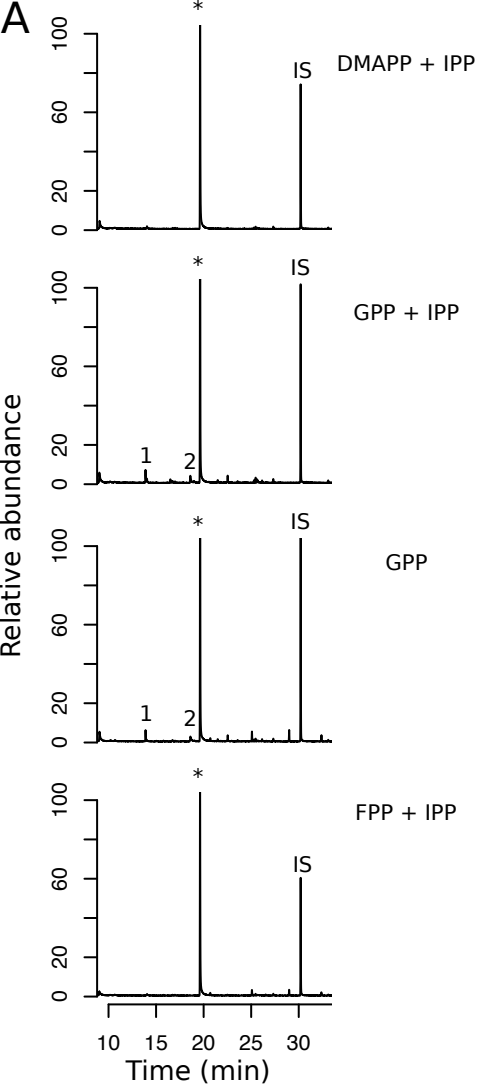**B**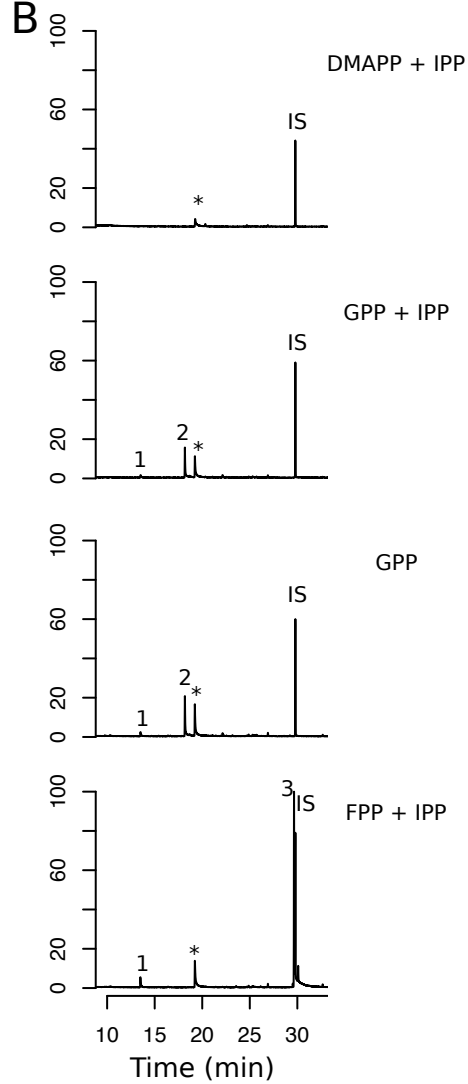**C**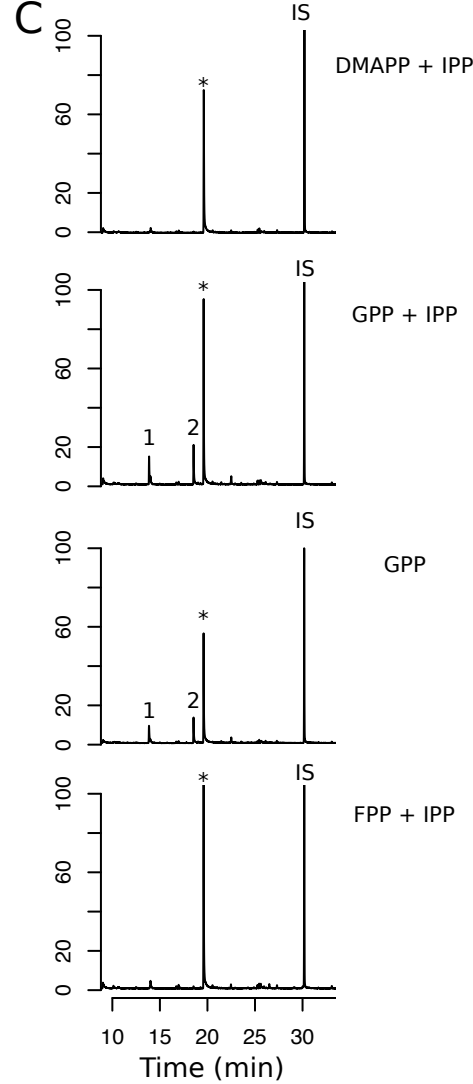

Supplement: S6 Fig — Control experiments (protein expression uninduced) for the functional characterisation of TPS activity of (A) HmelOS, (B) HcydOS, and (C) HMEL037108g1 from H. melpomene. Total ion chromatograms of products in the presence of different precursor compounds. (E)-β-Ocimene is not produced in any treatments. Linalool and geraniol are produced in small amounts in both, likely due to endogenous bacterial activity. 1, Linalool; 2, Geraniol; 3, Farnesol; *, contaminant from medium; IS, internal standard. Abundance is scaled to the highest peak of all panels per enzyme. Quantification of peaks in S4–S6 Tables. Raw GC/MS data are available from OSF (https://osf.io/3z9tg/). GC/MS, gas chromatography/mass spectrometry; TPS, terpene synthase. (PDF) [file pbio.3001022.s006.pdf]

**A**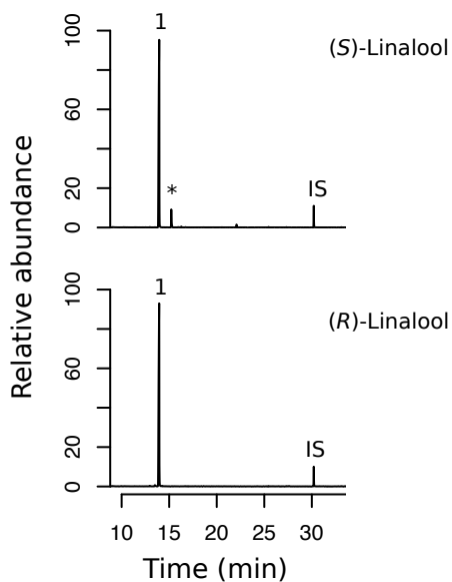**B**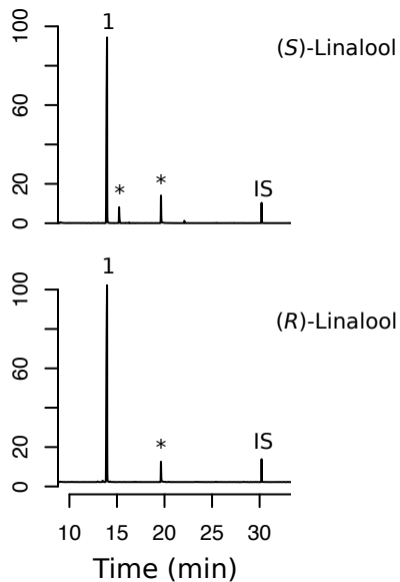

Supplement: S7 Fig — (A) Total ion chromatograms of enzymatic products in the presence of different linalool stereoisomers. No enzymatic activity is detected. (B) Total ion chromatograms of control experiments (protein expression not induced) in the presence of different Linalool stereoisomers. Again, as expected, no enzymatic activity is detected. 1, Linalool; *, contaminants from medium; IS, internal standard. Abundance is scaled to the highest peak of all panels. Quantification of peaks in S7 Table. Raw GC/MS data are available from OSF (https://osf.io/3z9tg/). GC/MS, gas chromatography/mass spectrometry. (PDF) [file pbio.3001022.s007.pdf]

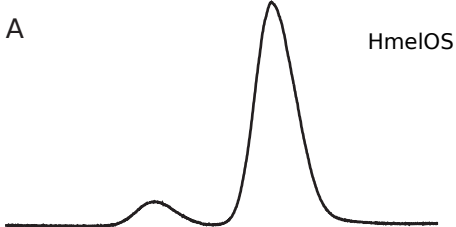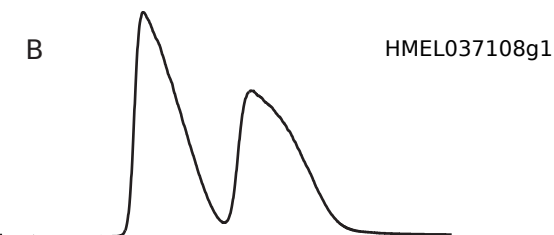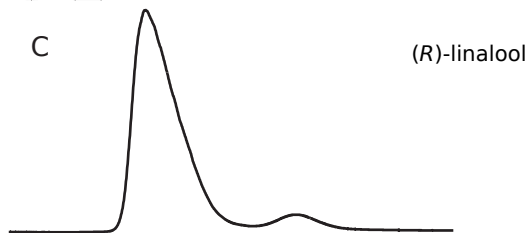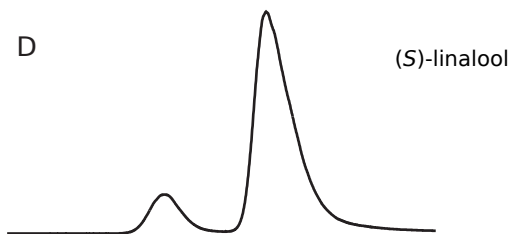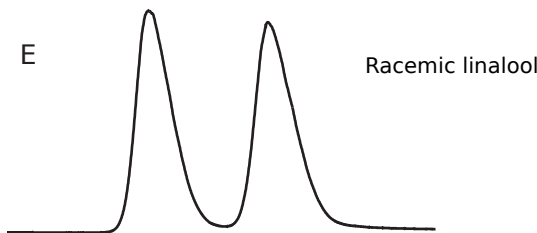

10.4 10.5 10.6 10.7 10.8 10.9

Supplement: S8 Fig — (A) Linalool produced in experiments with HmelOS is mainly (S)-linalool (ratio 97:3, S:R), (B) linalool produced in experiments with HMEL037108g1 is a racemic mixture (ratio 54:56, S:R), (C) (R)-linalool, (D) (S)-linalool, (E) Racemic linalool mixture. Raw GC/MS data are available from OSF (https://osf.io/3z9tg/). GC/MS, gas chromatography/mass spectrometry. (PDF) [file pbio.3001022.s008.pdf]

**A**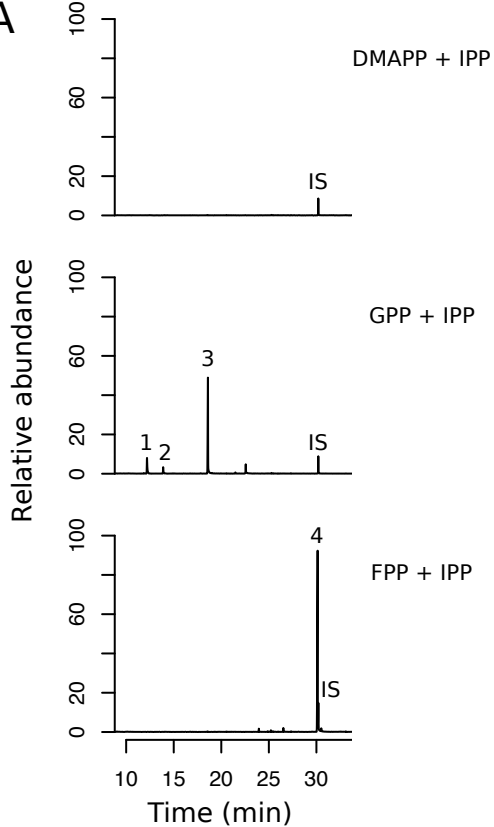**B**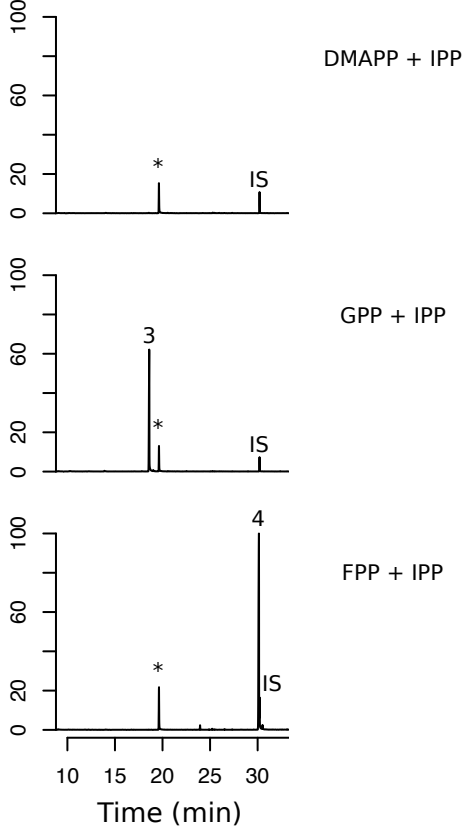

Supplement: S9 Fig — (A) Total ion chromatograms of enzymatic products in the presence of different precursor compounds, following treatment by alkaline phosphatase. GPP is dephosphorylated to produce geraniol, and FPP to produce farnesol, demonstrating that the main function of HmelOS is not as an IDS. (B) Total ion chromatograms of control experiments (protein expression not induced) in the presence of different precursor compounds, following treatment by alkaline phosphatase. As expected, GPP is dephosphorylated to geraniol, and FPP to farnesol. 1, (E)-β-Ocimene; 2, Linalool; 3, Geraniol; 4, Farnesol; *, contaminant from medium; IS, internal standard. Abundance is scaled to the highest peak of all panels. Quantification of peaks in S9 Table. Raw GC/MS data are available from OSF (https://osf.io/3z9tg/). FPP, farnesyl diphosphate; GC/MS, gas chromatography/mass spectrometry; GPP, geranyl diphosphate; IDS, isoprenyl diphosphate synthase. (PDF) [file pbio.3001022.s009.pdf]

**A**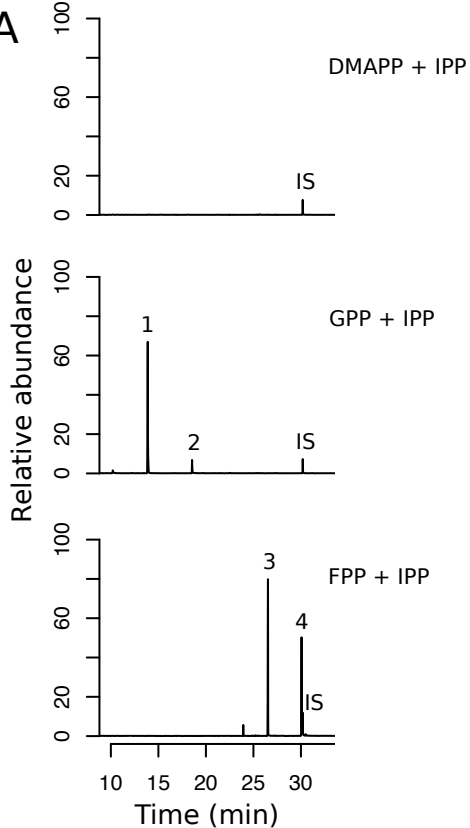**B**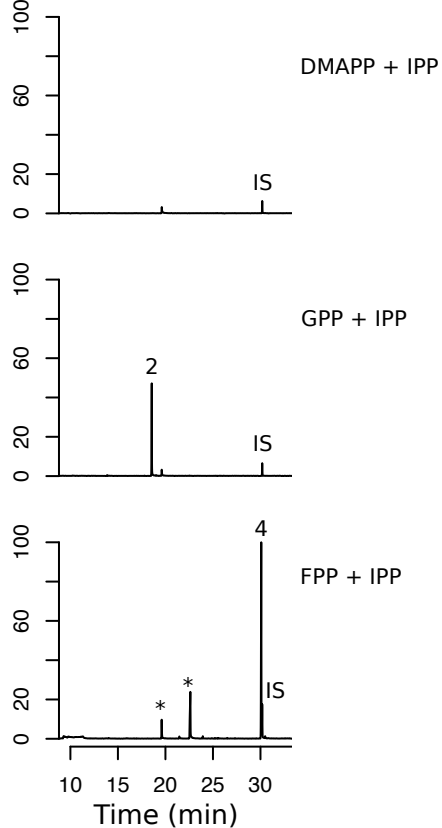

Supplement: S10 Fig — (A) Total ion chromatograms of enzymatic products in the presence of different precursor compounds, following treatment by alkaline phosphatase. As in Fig 3, GPP is converted to linalool and FPP to nerolidol, with remaining GPP dephosphorylated to geraniol, and FPP to farnesol. HMEL037108g1 is acting as a mono- and sesquiterpene synthase, not an IDS. (B) Total ion chromatograms of control experiments (protein expression not induced) in the presence of different precursor compounds, following treatment by alkaline phosphatase. GPP is dephosphorylated to geraniol and FPP to farnesol. 1, Linalool; 2, Geraniol; 3, Nerolidol; 4, Farnesol; *, contaminant from medium; IS, internal standard. Abundance is scaled to the highest peak of all panels. Quantification of peaks in S10 Table. Raw GC/MS data are available from OSF (https://osf.io/3z9tg/). FPP, farnesyl diphosphate; GC/MS, gas chromatography/mass spectrometry; GPP, geranyl diphosphate; IDS, isoprenyl diphosphate synthase. (PDF) [file pbio.3001022.s010.pdf]

**A**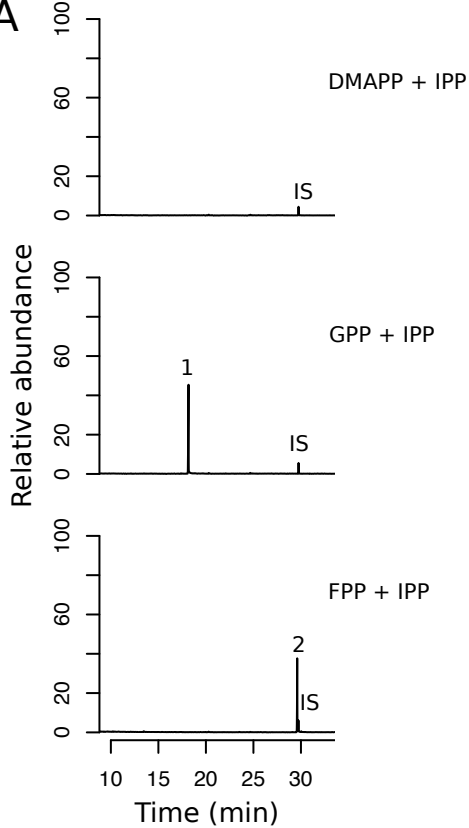**B**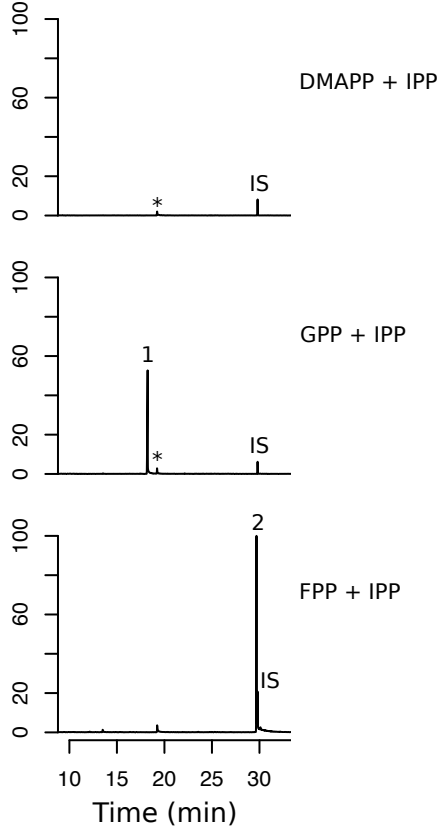

Supplement: S11 Fig — (A) Total ion chromatograms in the presence of different precursor compounds, following treatment by alkaline phosphatase. GPP is dephosphorylated to produce geraniol, and FPP to produce farnesol, demonstrating that the HcydOS is not an IDS. (B) Total ion chromatograms of control experiments (protein expression not induced) in the presence of different precursor compounds, following treatment by alkaline phosphatase. As expected, GPP is dephosphorylated to geraniol, and FPP to farnesol. 1, Geraniol; 2, Farnesol; *, contaminant from medium; IS, internal standard. Abundance is scaled to the highest peak of all panels. Quantification of peaks in S11 Table. Raw GC/MS data are available from OSF (https://osf.io/3z9tg/). FPP, farnesyl diphosphate; GC/MS, gas chromatography/mass spectrometry; GPP, geranyl diphosphate; IDS, isoprenyl diphosphate synthase. (PDF) [file pbio.3001022.s011.pdf]

**A**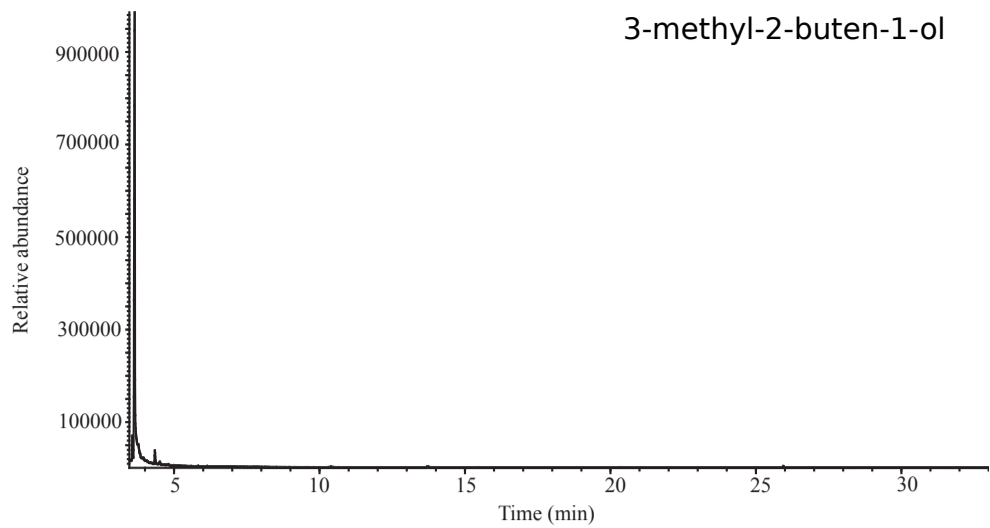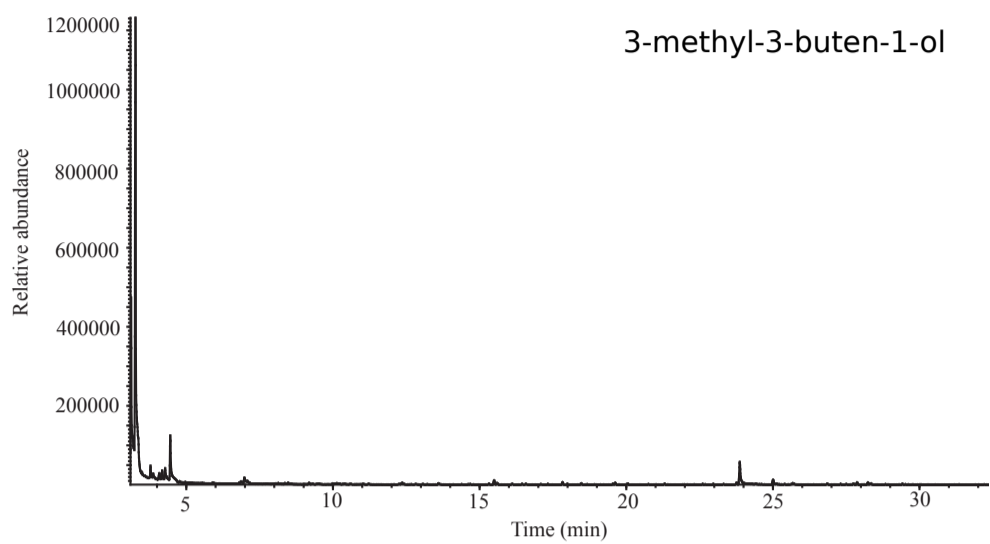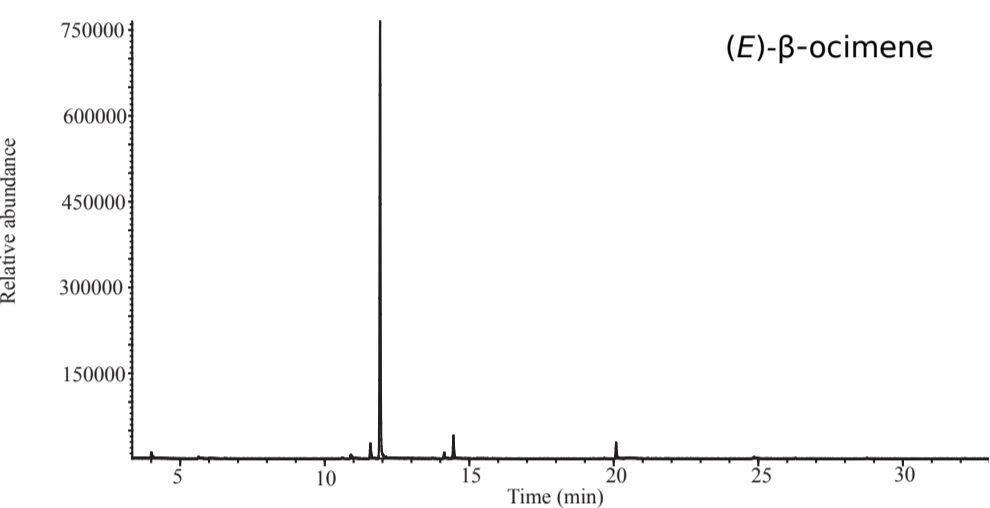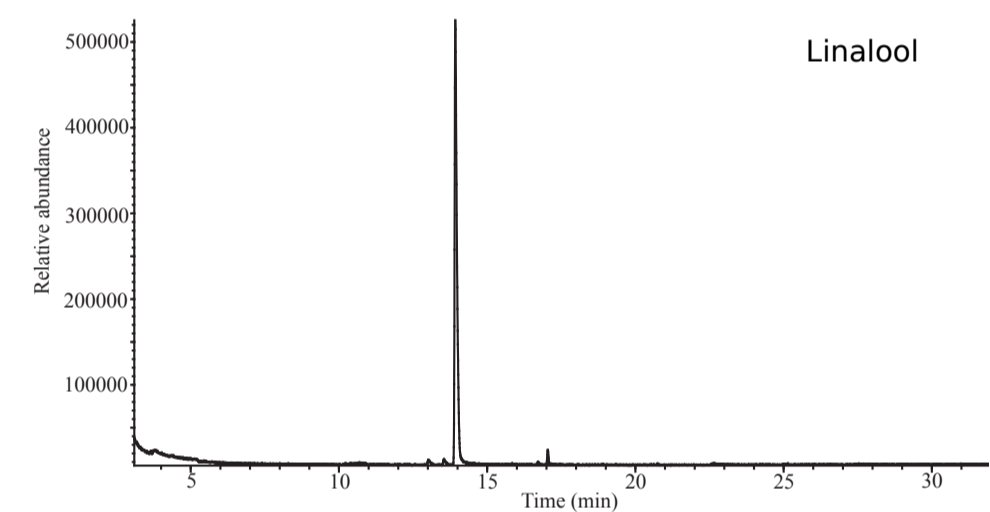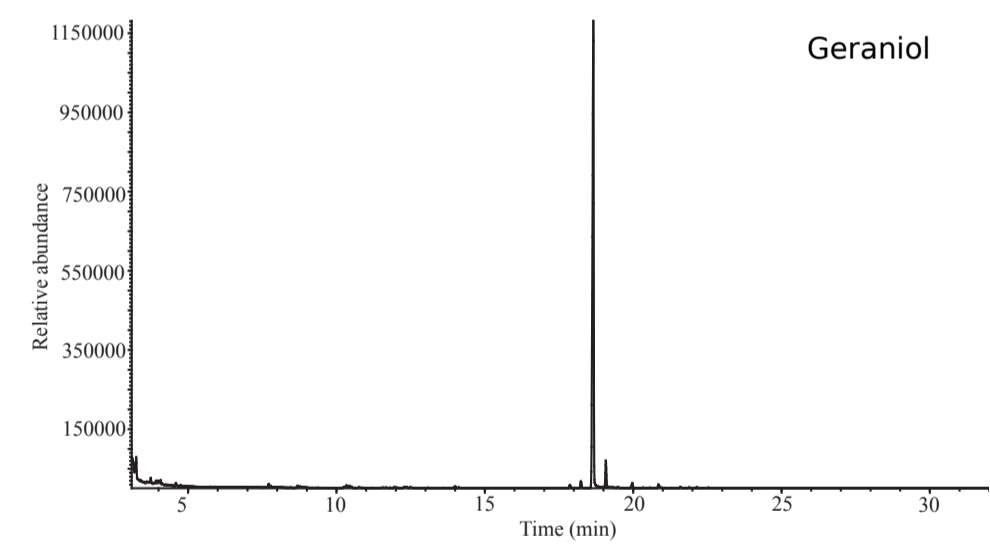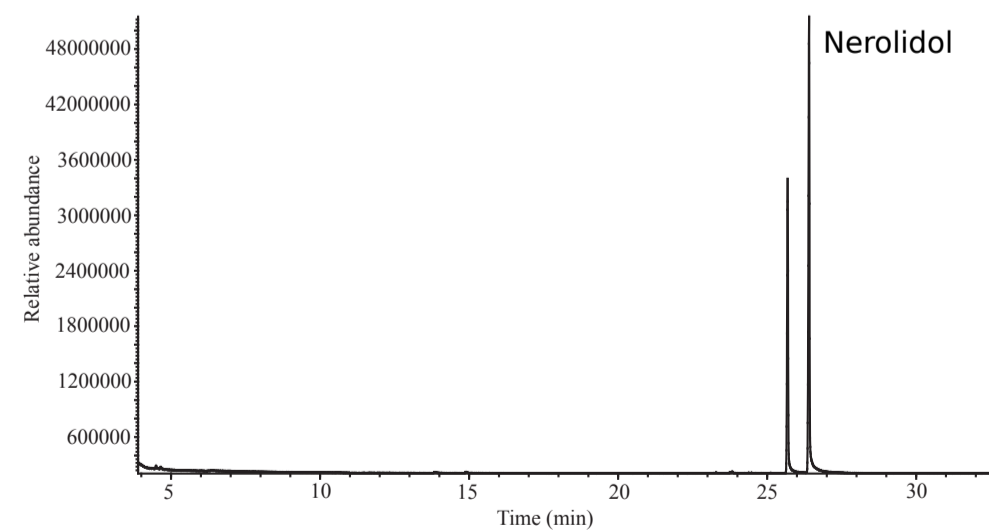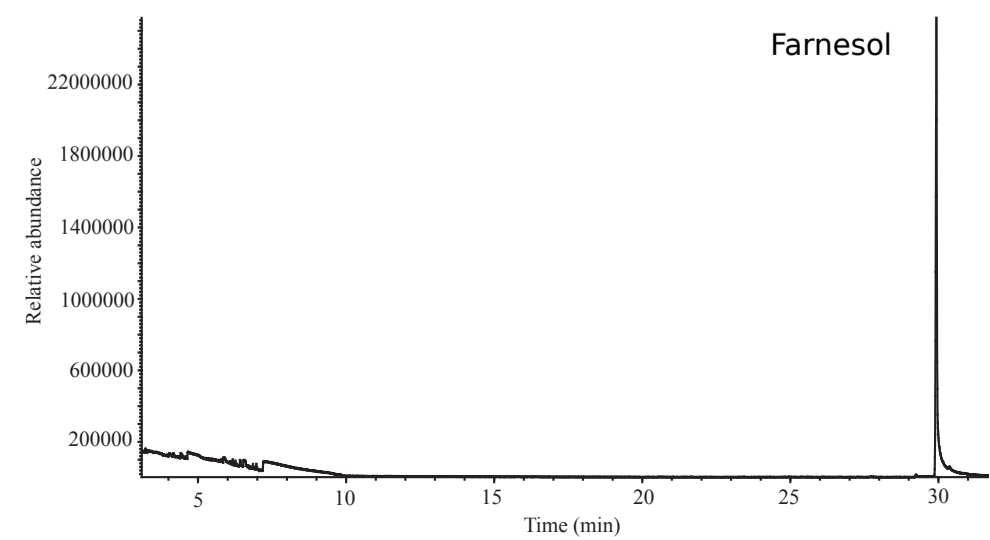**B**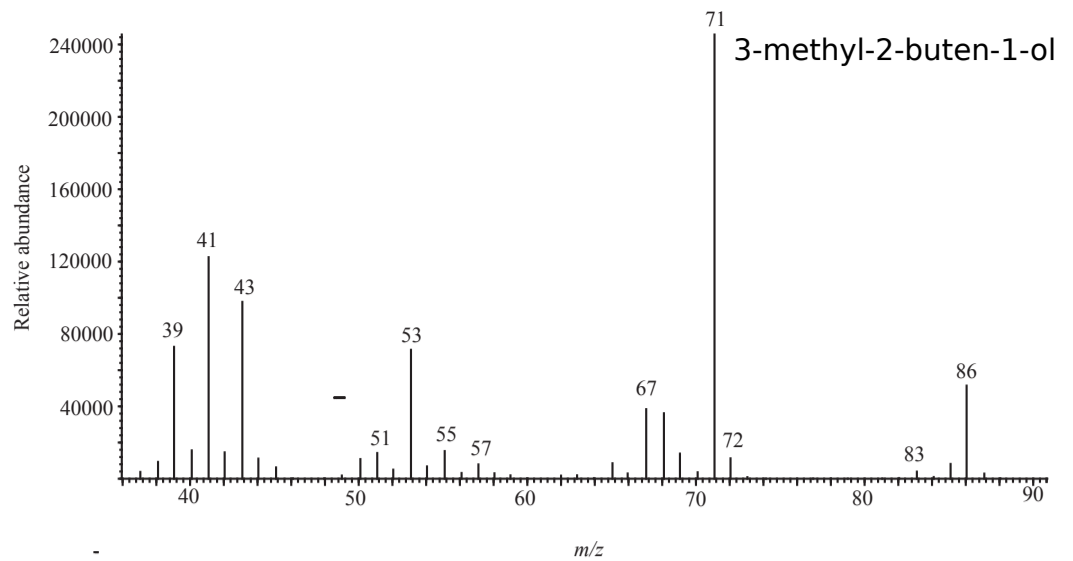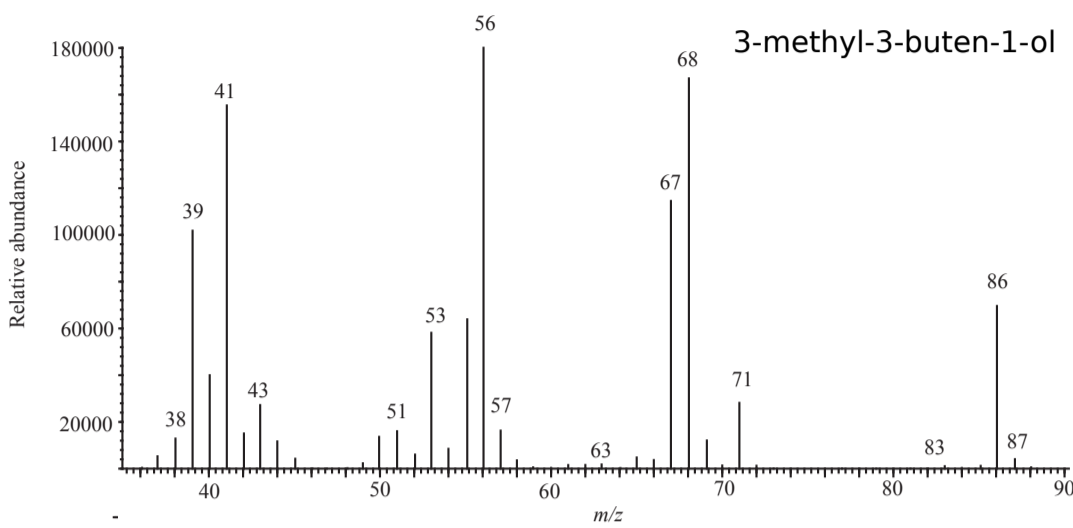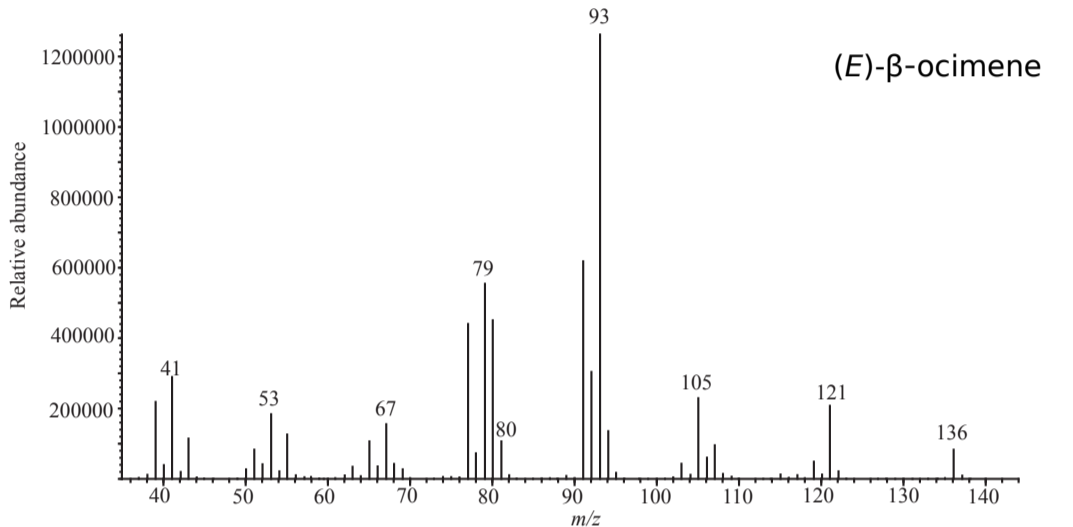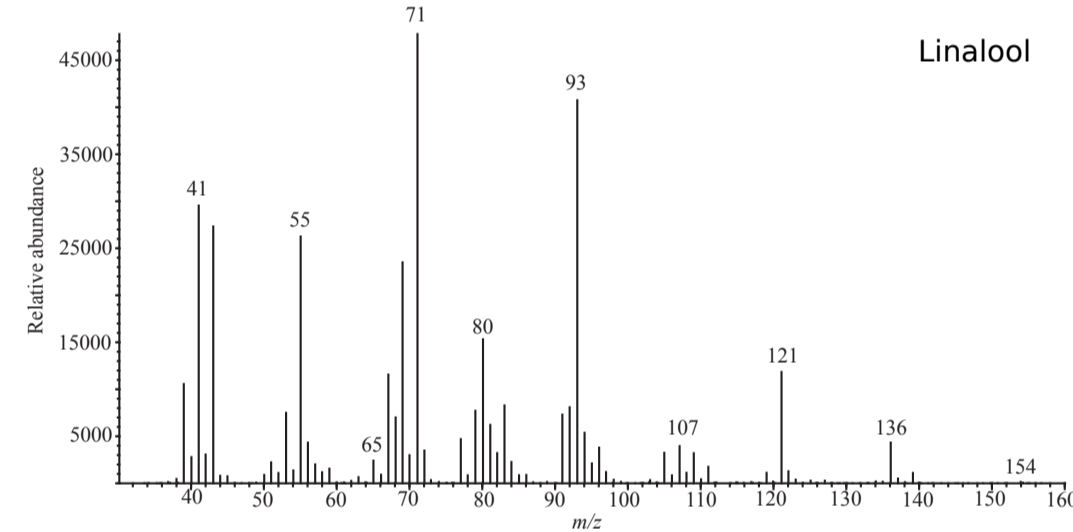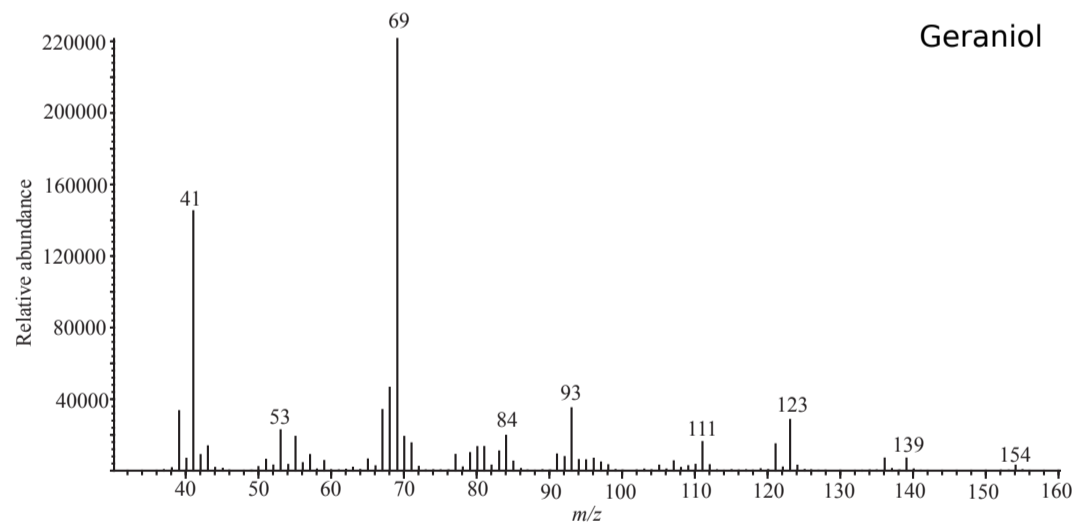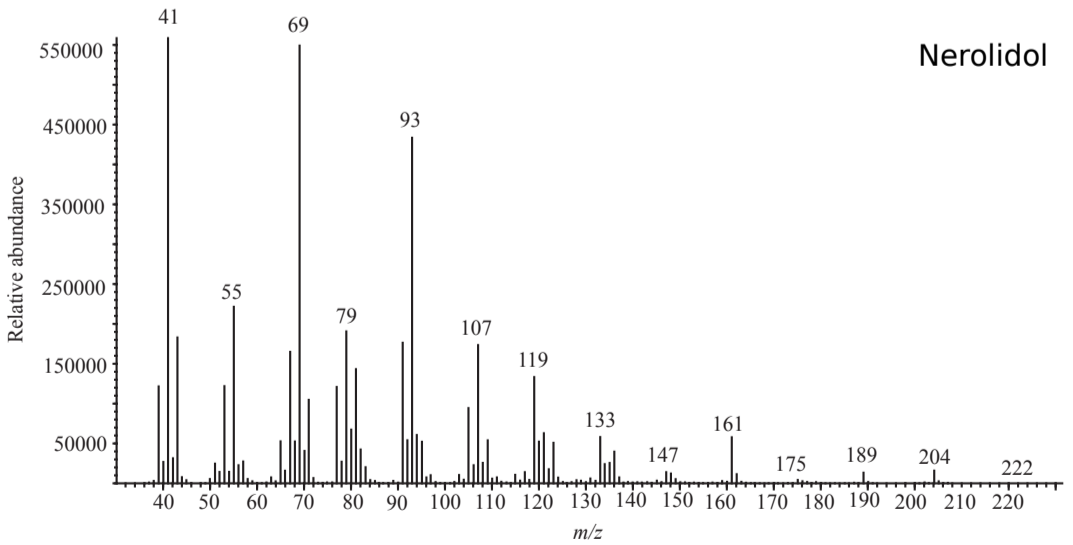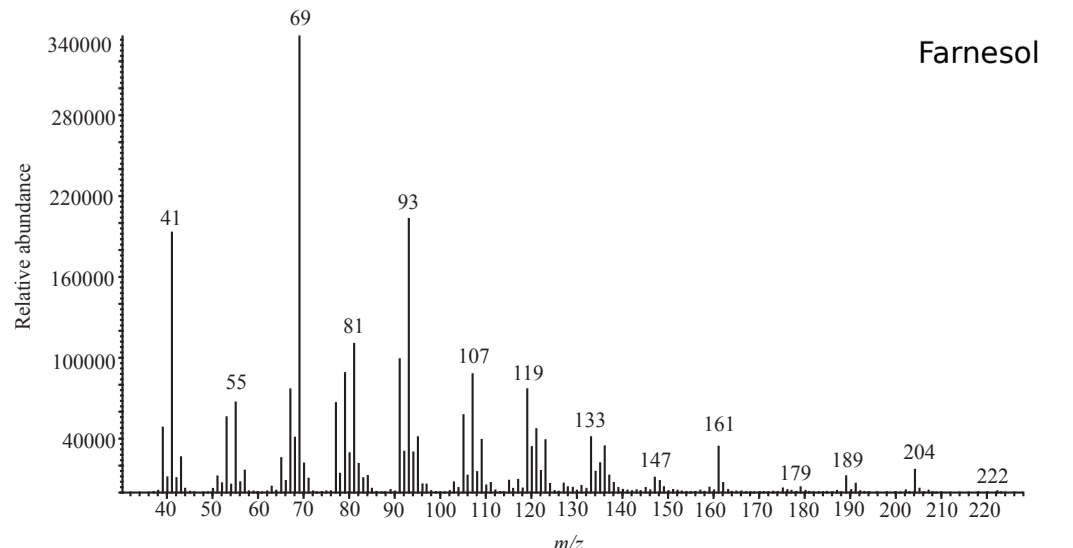

Supplement: S12 Fig — Chromatograms (A) and mass spectra (B) of standards. Nerolidol is a mix of stereoisomers. Raw GC/MS data are available from OSF (https://osf.io/3z9tg/). GC/MS, gas chromatography/mass spectrometry. (PDF) [file pbio.3001022.s012.pdf]

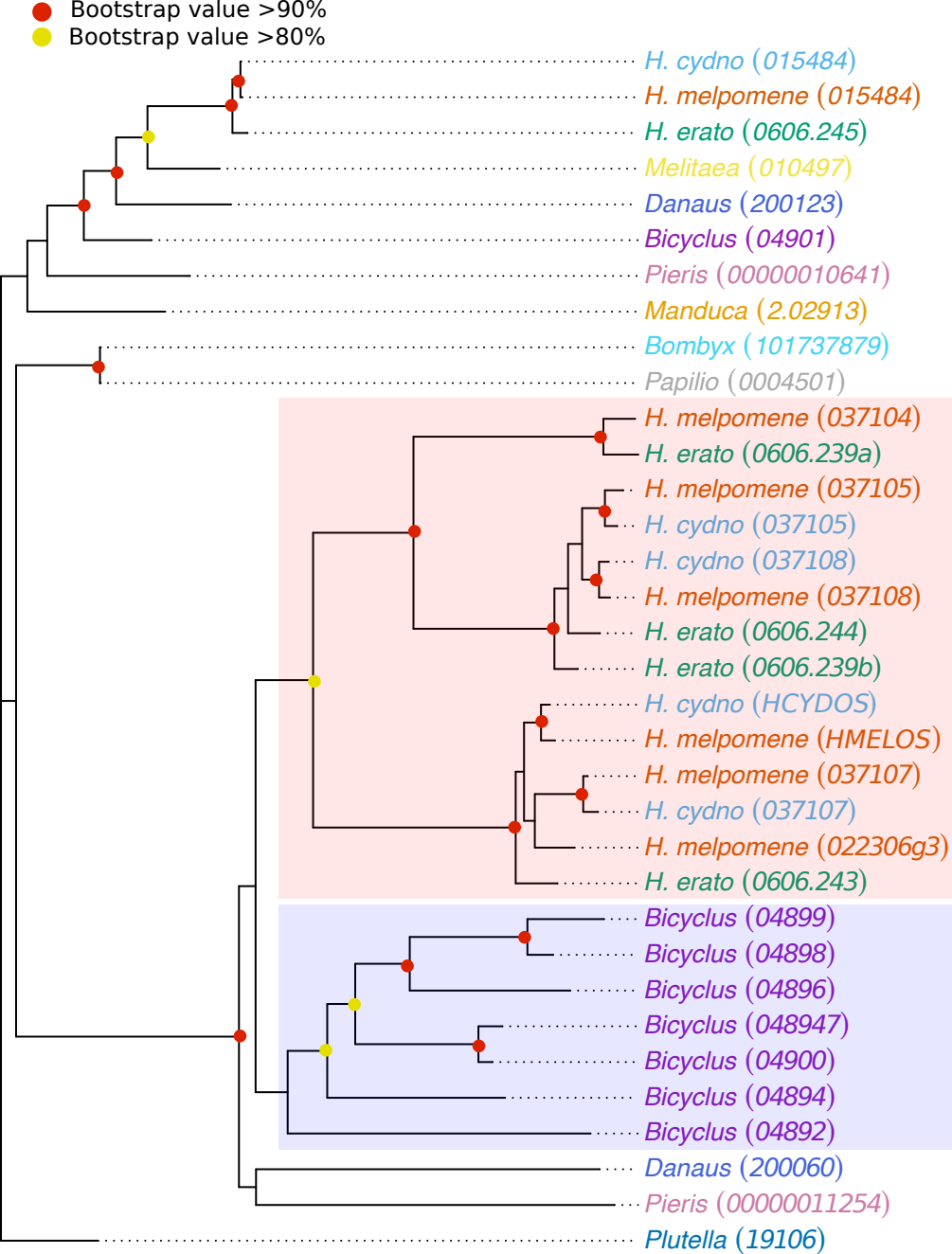

Supplement: S13 Fig — The clades which have undergone lineage-specific expansion of the GGPPS families, Heliconius and Bicyclus, are shaded red and blue, respectively. The phylogeny was constructed in PhyML using the model GTR + G + I. Bootstrap values (n = 1,000) are illustrated. Script is available from OSF (https://osf.io/3z9tg/). GGPPS, geranylgeranyl diphosphate synthase. (PDF) [file pbio.3001022.s013.pdf]

*H. erato* (Herato0606.245)

● Bootstrap value >90%

*H. cydno* (HMEL015484g1)

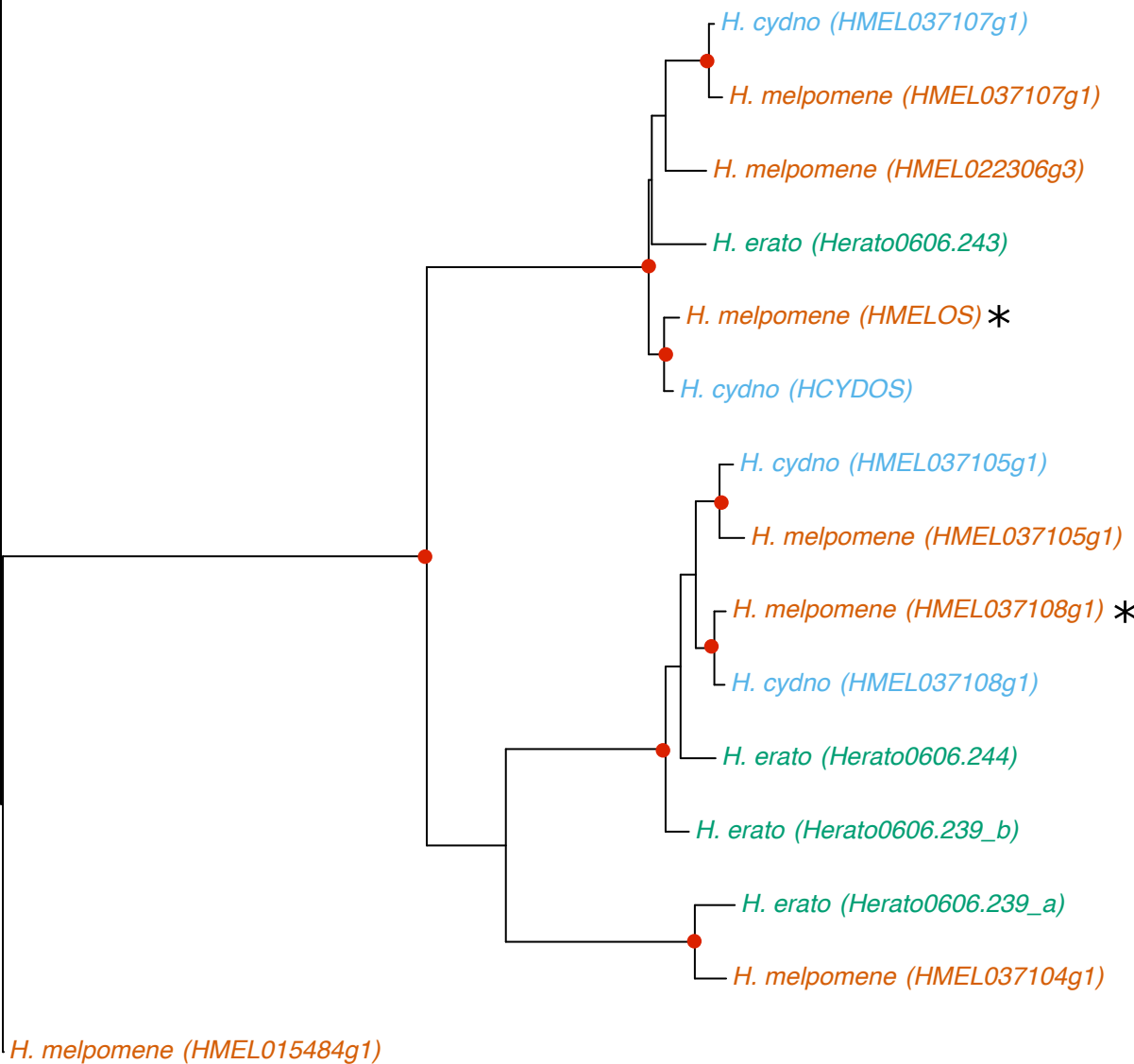

Supplement: S14 Fig — These include HMELOS and HMEL037108g1 (*) which encode TPSs. The phylogeny was constructed in PhyML using the model GTR + G + I. Bootstrap values (n = 1,000) are illustrated. The H. erato gene Herato0606.245 (GGPPS, shows high similarity to the GGPPS of the moth Choristoneura fumiferana) was used to root the tree. Script is available from OSF (https://osf.io/3z9tg/). GGPPS, geranylgeranyl diphosphate synthase; TPS, terpene synthase. (PDF) [file pbio.3001022.s014.pdf]

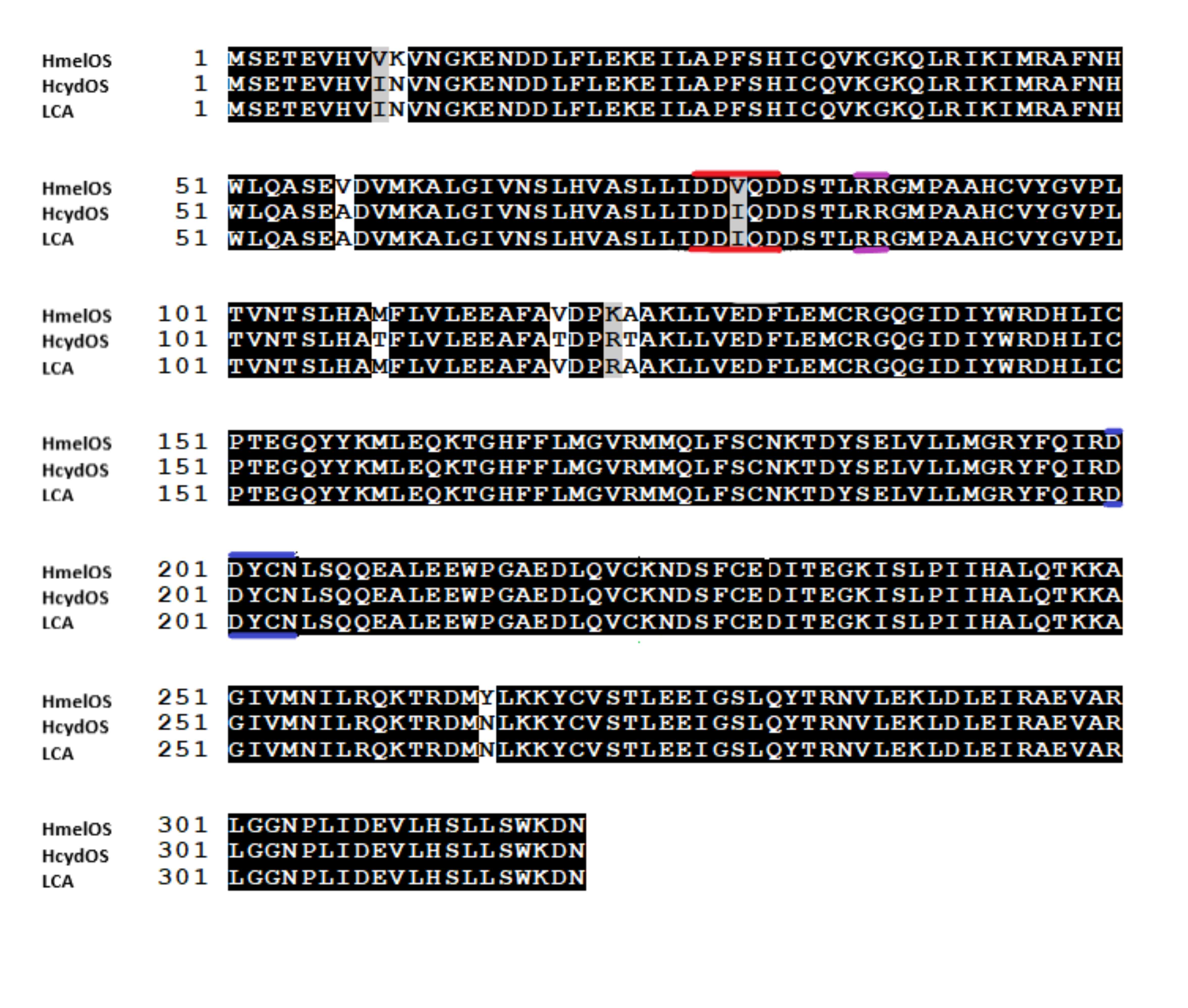

Supplement: S15 Fig — All amino acid substitutions occurred at ancestral protein sites constructed with high posterior probability (>0.9), except 1 substitution between the ancestral sequence and HcydOS (M109T) which occurred at a site with a posterior probability >0.8. Amino acid sites shared in all 3 sequences are shaded black. Polymorphic sites are shaded white or grey, with grey shading indicating a substitution to an amino acid with similar chemical properties as calculated in the BOXSHADE software (https://embnet.vital-it.ch/software/BOX_form.html). LCA, last common ancestor; TPS, terpene synthase. (PNG) [file pbio.3001022.s015.png]
