# Supplementary material for: A novel terpene synthase controls differences in anti-aphrodisiac pheromone production between closely related Heliconius butterflies
Source: PLoS Biol. 2021 Jan 19;19(1):e3001022. doi: 10.1371/journal.pbio.3001022 (PMC7815096; doi:10.1371/journal.pbio.3001022)
Supplement: S1 Table — Sequences were downloaded from FlyBase and searched (blastp) against all annotated proteins in the genome of Heliconius melpomene (v2.5) on LepBase to identify homologs of enzymes involved in the mevalonate and putative terpene synthesis pathways. The candidate orthologs identified in H. melpomene were then searched (blastp) against annotated proteins in the D. melanogaster genome on FlyBase. Reciprocal best blasts are highlighted in bold. We included other hits with an e-value smaller than 1e-80. (DOCX) [file pbio.3001022.s017.docx]

| Gene (symbol) | *D. melanogaster* | *H. melpomene* |
| --- | --- | --- |
| Acetoacetyl-CoA thiolase (ACAT2) | CG9149 | HMEL032609g1  HMEL014614g2  HMEL017484g1 |
| Hydroxymethylglutaryl-CoA synthase (HMGCS) | CG4311 | **HMEL005451g1** |
| Hydroxymethylglutaryl-CoA reductase (HMGCR) | CG10367 | **HMEL016133g1** |
| Mevalonate kinase (MVK) | CG33671 | **HMEL013262g2** |
| Phosphomevalonate kinase (PMVK) | CG10268 | **HMEL007429g4** |
| Diphosphomevalonate decarboxylase (MVD) | CG8239 | **HMEL004012g1** |
| Isopentenyl-diphosphate isomerase (IDI) | CG5919 | **HMEL005103g1** |
| Farnesyl pyrophosphate synthase (FPPS) | CG12389 | **HMEL017961g1**  HMEL017961g2 |
| Geranylgeranyl pyrophosphate synthase (GGPPS) | CG8593 | **HMEL015484g1**  HMEL037104g1  HMEL037105g1  HmelOS (HMEL037106g1)  HMEL022306g3  HMEL037107g1  HMEL037108g1 |
| Decaprenyl pyrophosphate synthase subunit 1 (PDSS1) | CG31005 | **HMEL016759g1** |
| Decaprenyl pyrophosphate synthase subunit 2 (PDSS2) | CG10585 | **HMEL031784g1**  HMEL011234g1  HMEL008172g1  HMEL008173g1 |
