# Supplementary material for: A novel terpene synthase controls differences in anti-aphrodisiac pheromone production between closely related Heliconius butterflies
Source: PLoS Biol. 2021 Jan 19;19(1):e3001022. doi: 10.1371/journal.pbio.3001022 (PMC7815096; doi:10.1371/journal.pbio.3001022)
Supplement: S2 Table — The model includes 2 fixed terms, tissue and sex, their interaction, and a random term, individual (expression ~ sex + tissue + sex*tissue + (1|individual). The Log FC column gives the log2 Fold Change between the groups being compared, while the Ave. Expr. column gives the mean log2-exprtession across all samples. Column t is the moderated t-statistic, and B is the B-statistic; the log odds that the gene is differentially expressed. The Adj. p-value column gives p-values (bold are significant) corrected for multiple testing using the Benjamini and Hochberg’s method to control the false discovery rate across all tested genes (17,902). RNA-seq data of H. cydno and H. melpomene heads and abdomens was obtained from GenBank BioProject PRJNA283415. Processed data and scripts are available from OSF (https://osf.io/3z9tg/). RNA-seq, RNA sequencing. (DOCX) [file pbio.3001022.s018.docx]

| Genes | Term | LogFC | Ave. Expr. | t | p-value | Adj. p-value | B |
| --- | --- | --- | --- | --- | --- | --- | --- |
| *HMEL015484g1* | tissue*sex | 0.4632 | 4.0453 | 1.1730 | 0.2541 | 0.5058 | -6.3516 |
|  | sex | -0.2464 | 4.0453 | -0.6806 | 0.5037 | 0.6876 | -6.8947 |
|  | tissue | 1.3737 | 4.0453 | 5.0813 | 0.0001 | **0.0003** | 1.3419 |
| *HMEL016759g1* | tissue*sex | 0.6666 | 4.3428 | 1.3651 | 0.1869 | 0.4149 | -6.1281 |
|  | sex | -0.7169 | 4.3428 | -1.5598 | 0.1339 | 0.3151 | -5.9214 |
|  | tissue | 2.0107 | 4.3428 | 6.1591 | 4.37E-06 | **3.11E-05** | 3.8018 |
| *HMEL022306g3* | tissue*sex | -1.7771 | -2.6450 | -2.0499 | 0.0532 | 0.1788 | -4.3831 |
|  | sex | 1.0504 | -2.6450 | 1.4828 | 0.1532 | 0.3464 | -5.4472 |
|  | tissue | -0.8092 | -2.6450 | -1.3167 | 0.2023 | 0.2989 | -5.7745 |
| *HMEL037104g1* | tissue*sex | 2.1661 | 0.2926 | 2.7261 | 0.0127 | 0.0621 | -3.2905 |
|  | sex | -2.8916 | 0.2926 | -4.7047 | 0.0001 | **0.0010** | 0.9565 |
|  | tissue | -2.3371 | 0.2926 | -4.7057 | 0.0001 | **0.0006** | 0.8174 |
| *HMEL037105g1* | tissue*sex | 3.0898 | -0.6587 | 2.6632 | 0.0146 | 0.0689 | -3.3185 |
|  | sex | -6.1709 | -0.6587 | -7.4086 | 2.99E-07 | **3.85E-06** | 6.9326 |
|  | tissue | -5.3865 | -0.6587 | -7.7309 | 1.55E-07 | **1.75E-06** | 7.5347 |
| *HMELOS* | tissue*sex | -4.8365 | 3.0873 | -4.3253 | 0.0003 | **0.0029** | 0.1997 |
|  | sex | 3.9194 | 3.0873 | 7.6829 | 1.70E-07 | **2.27E-06** | 7.1289 |
|  | tissue | -5.0459 | 3.0873 | -6.6164 | 1.60E-06 | **1.32E-05** | 5.1659 |
| *HMEL037107g1* | tissue*sex | -1.5552 | -1.2880 | -1.4148 | 0.1720 | 0.3932 | -5.4320 |
|  | sex | 0.2361 | -1.2880 | 0.2584 | 0.7986 | 0.8901 | -6.5545 |
|  | tissue | 0.0636 | -1.2880 | 0.0843 | 0.9337 | 0.9540 | -6.7475 |
| *HMEL037108g1* | tissue*sex | -1.9572 | 1.1234 | -1.7327 | 0.0980 | 0.2715 | -5.2129 |
|  | sex | 2.0833 | 1.1234 | 2.3946 | 0.0262 | 0.0952 | -4.2929 |
|  | tissue | -0.2800 | 1.1234 | -0.3378 | 0.7389 | 0.8095 | -6.8804 |
