# Supplementary material for: A novel terpene synthase controls differences in anti-aphrodisiac pheromone production between closely related Heliconius butterflies
Source: PLoS Biol. 2021 Jan 19;19(1):e3001022. doi: 10.1371/journal.pbio.3001022 (PMC7815096; doi:10.1371/journal.pbio.3001022)
Supplement: S3 Table — The model includes 2 fixed terms, species and sex, and their interaction (expression ~ sex + species + species*tissue). The Log FC column gives the log2 Fold Change between the groups being compared, while the Ave. Expr. column gives the mean log2-exprtession across all samples. Column t is the moderated t-statistic, and B is the B-statistic; the log odds that the gene is differentially expressed. The Adj. p-value column gives p-values (bold are significant) corrected for multiple testing using the Benjamini and Hochberg’s method to control the false discovery rate across all tested genes (11,571). RNA-seq data of H. cydno and H. melpomene heads and abdomens was obtained from GenBank BioProject PRJNA283415. Processed data and scripts are available from OSF (https://osf.io/3z9tg/). RNA-seq, RNA sequencing. (DOCX) [file pbio.3001022.s019.docx]

| Genes | Term | LogFC | Ave. Expr. | t | | p-value | Adj. p-value | B | |  |
| --- | --- | --- | --- | --- | --- | --- | --- | --- | --- | --- |
| *HMEL015484g1* | species*sex | -0.2867 | 4.0551 | | -3.8411 | 0.0010 | **0.0121** | | -1.9011 | |
|  | species | 0.2750 | 4.0551 | | 3.6847 | 0.0014 | **0.0033** | | -2.7865 | |
|  | sex | -0.1316 | 4.0551 | | -1.7634 | 0.0929 | 0.1845 | | -6.5891 | |
| *HMEL016759g1* | species*sex | -0.3223 | 5.4115 | | -1.4758 | 0.1554 | 0.4355 | | -6.6856 | |
|  | species | 1.7247 | 5.4115 | | 7.8963 | 1.28E-07 | **4.45E-07** | | 6.6325 | |
|  | sex | 0.0672 | 5.4115 | | 0.3075 | 0.7616 | 0.8457 | | -8.1216 | |
| *HMEL022306g3* | species*sex | -0.6189 | 0.9789 | | -1.3185 | 0.2020 | 0.4949 | | -6.3958 | |
|  | species | 2.2812 | 0.9789 | | 4.8597 | 0.0001 | **0.0003** | | 0.4004 | |
|  | sex | -1.1124 | 0.9789 | | -2.3697 | 0.0278 | 0.0693 | | -5.0730 | |
| *HMEL037104g1* | species*sex | -0.6172 | 0.5317 | | -2.9442 | 0.0079 | 0.0642 | | -3.4631 | |
|  | species | -1.0353 | 0.5317 | | -4.9387 | 0.0001 | **0.0002** | | 0.6241 | |
|  | sex | 0.8629 | 0.5317 | | 4.1160 | 0.0005 | **0.0022** | | -1.1857 | |
| *HMEL037105g1* | species*sex | -0.1486 | 2.5652 | | -0.7572 | 0.4576 | 0.7265 | | -7.0871 | |
|  | species | 0.6682 | 2.5652 | | 3.4050 | 0.0028 | **0.0061** | | -3.1103 | |
|  | sex | 2.9915 | 2.5652 | | 15.2445 | 1.39E-12 | **3.25E-11** | | 18.8669 | |
| *HMELOS*  */HCYDOS* | species*sex | 0.8481 | 6.7281 | | 3.1533 | 0.0049 | **0.0445** | | -3.6918 | |
|  | species | -0.7368 | 6.7281 | | -2.7396 | 0.0125 | **0.0237** | | -5.1145 | |
|  | sex | -1.0795 | 6.7281 | | -4.0137 | 0.0007 | **0.0027** | | -2.1159 | |
| *HMEL037107g1* | species*sex | -0.2142 | 1.4255 | | -0.7733 | 0.4483 | 0.7200 | | -7.0073 | |
|  | species | 1.9002 | 1.4255 | | 6.8605 | 1.06E-06 | **3.44E-06** | | 4.8921 | |
|  | sex | -0.3277 | 1.4255 | | -1.1833 | 0.2504 | 0.3913 | | -7.0076 | |
| *HMEL037108g1* | species*sex | 1.1937 | 1.5269 | | 3.4431 | 0.0025 | **0.0259** | | -2.4506 | |
|  | species | -0.8122 | 1.5269 | | -2.3428 | 0.0294 | 0.0508 | | -5.3095 | |
|  | sex | 0.1371 | 1.5269 | | 0.3954 | 0.6967 | 0.7980 | | -7.6424 | |
