# Supplementary material for: A novel terpene synthase controls differences in anti-aphrodisiac pheromone production between closely related Heliconius butterflies
Source: PLoS Biol. 2021 Jan 19;19(1):e3001022. doi: 10.1371/journal.pbio.3001022 (PMC7815096; doi:10.1371/journal.pbio.3001022)
Supplement: S4 Table — HmelOS is a monoterpene synthase, catalysing the formation of (E)-β-ocimene from GPP. Residual IDS activity is shown by the production of (E)-β-ocimene, linalool, and nerolidol from DMAPP and IPP. Mean amounts (ng) ± standard deviation for each compound across 3 replicates are shown. (control) indicates experiments where protein expression was not induced. N = 3 for each treatment. Raw GC/MS data and quantification of each sample are available from OSF (https://osf.io/3z9tg/). DMAPP, dimethylallyl diphosphate; GC/MS, gas chromatography/mass spectrometry; GPP, geranyl diphosphate; IDS, isoprenyl diphosphate synthase; IPP, isopentenyl diphosphate; TPS, terpene synthase. (DOCX) [file pbio.3001022.s020.docx]

|  | (*E*)-β-Ocimene | (*Z*)-β-Ocimene | Linalool | Geraniol | Nerolidol |
| --- | --- | --- | --- | --- | --- |
| DMAPP + IPP | 7.8±1.4 | 0±0 | 3.4±0.4 | 0±0 | 4.4±1.8 |
| DMAPP + IPP (control) | 0±0 | 0±0 | 0±0 | 0±0 | 0±0 |
| GPP + IPP | 334.7±32.7 | 10.7±1.5 | 84.2±7.7 | 36±5.2 | 0±0 |
| GPP + IPP (control) | 0±0 | 0±0 | 17.2±2.6 | 17.4±4.6 | 0±0 |
| GPP | 356.5±115.3 | 12.0±4.4 | 108.4±37.3 | 66.6±23 | 0±0 |
| GPP (control) | 0±0 | 0±0 | 10.4±9 | 18.5±6 | 0±0 |
| FPP + IPP | 0±0 | 0±0 | 0±0 | 0±0 | 12.3±0.8 |
| FPP + IPP (control) | 0±0 | 0±0 | 0±0 | 0±0 | 2.1±0.3 |
