# Supplementary material for: A novel terpene synthase controls differences in anti-aphrodisiac pheromone production between closely related Heliconius butterflies
Source: PLoS Biol. 2021 Jan 19;19(1):e3001022. doi: 10.1371/journal.pbio.3001022 (PMC7815096; doi:10.1371/journal.pbio.3001022)
Supplement: S5 Table — HMEL037108g1 acts as a mono- and sesquiterpene synthase, producing linalool from GPP and nerolidol from FPP. Small amounts of linalool and nerolidol detected in DMAPP and IPP treatment, and of nerolidol in the GPP treatment, demonstrate residual IDS activity. Mean amounts (ng) ± standard deviation for each compound across 3 replicates are shown. N = 3 for each treatment. Raw GC/MS data and quantification of each sample are available from OSF (https://osf.io/3z9tg/). DMAPP, dimethylallyl diphosphate; FPP, farnesyl diphosphate; GC/MS, gas chromatography/mass spectrometry; GPP, geranyl diphosphate; IPP, isopentenyl diphosphate; TPS, terpene synthase. (DOCX) [file pbio.3001022.s021.docx]

|  | (*E*)-β-Ocimene | Linalool | Geraniol | Nerolidol | Farnesol |
| --- | --- | --- | --- | --- | --- |
| DMAPP + IPP | 0±0 | 8.5±0.5 | 0±0 | 10.2±0.5 | 0±0 |
| DMAPP + IPP (control) | 0±0 | 0±0 | 0±0 | 0±0 | 0±0 |
| GPP + IPP | 11.0±1.4 | 2908.3±361.4 | 109.3±21.6 | 0±0 | 0±0 |
| GPP + IPP (control) | 0±0 | 44.5±3.0 | 63.6±1.7 | 0±0 | 0±0 |
| GPP | 16.8±1.5 | 4040.0±404.1 | 122.2±15.8 | 11.1±1.0 | 0±0 |
| GPP (control) | 0±0 | 40.8±5.1 | 57.8±1.2 | 0±0 | 0±0 |
| FPP + IPP | 0±0 | 0±0 | 0±0 | 1734.9±165.7 | 21.5±3.3 |
| FPP + IPP (control) | 0±0 | 0±0 | 0±0 | 4.0±0.2 | 0±0 |
