# Supplementary material for: A novel terpene synthase controls differences in anti-aphrodisiac pheromone production between closely related Heliconius butterflies
Source: PLoS Biol. 2021 Jan 19;19(1):e3001022. doi: 10.1371/journal.pbio.3001022 (PMC7815096; doi:10.1371/journal.pbio.3001022)
Supplement: S6 Table — We find no evidence for TPS activity. Mean amounts (ng) ± standard deviation for each compound across 3 replicates are shown. N = 3 for each treatment. Raw GC/MS data and quantification of each sample are available from OSF (https://osf.io/3z9tg/). GC/MS, gas chromatography/mass spectrometry; TPS, terpene synthase. (DOCX) [file pbio.3001022.s022.docx]

|  | (*E*)-β-Ocimene | Linalool | Geraniol | Nerolidol | Farnesol |
| --- | --- | --- | --- | --- | --- |
| DMAPP + IPP | 0±0 | 0±0 | 0±0 | 0±0 | 0±0 |
| DMAPP + IPP (control) | 0±0 | 0±0 | 0±0 | 0±0 | 0±0 |
| GPP + IPP | 0±0 | 2.4±0.6 | 4.7±1.5 | 0±0 | 0±0 |
| GPP + IPP (control) | 0±0 | 5.4±1.4 | 51.4±11.5 | 0±0 | 0±0 |
| GPP | 0±0 | 3.0±0.7 | 6.7±1.2 | 0±0 | 0±0 |
| GPP (control) | 0±0 | 7.0±1.4 | 55.6±4.4 | 0±0 | 0±0 |
| FPP + IPP | 0±0 | 6.2±4.7 | 0±0 | 0±0 | 44.5±23.4 |
| FPP + IPP (control) | 0±0 | 11.0±3.5 | 0±0 | 0.4±0.3 | 113.4±13.6 |
