# Supplementary material for: A novel terpene synthase controls differences in anti-aphrodisiac pheromone production between closely related Heliconius butterflies
Source: PLoS Biol. 2021 Jan 19;19(1):e3001022. doi: 10.1371/journal.pbio.3001022 (PMC7815096; doi:10.1371/journal.pbio.3001022)
Supplement: S7 Table — HmelOS does not show enzymatic activity with linalool, demonstrating it is not an intermediate in the synthesis of (E)-β-ocimene. Mean amounts (ng) ± standard deviation for each compound across 3 replicates are shown. N = 3 for each treatment. Raw GC/MS data and quantification of each sample available from OSF (https://osf.io/3z9tg/). GC/MS, gas chromatography/mass spectrometry. (DOCX) [file pbio.3001022.s023.docx]

|  | (*E*)-β-Ocimene | (*Z*)-β-Ocimene | Linalool |
| --- | --- | --- | --- |
| (*S*)-Linalool | 0±0 | 0±0 | 3182.4±445.5 |
| (*S*)-Linalool (control) | 0±0 | 0±0 | 2698.9±1020.8 |
| (*R*)-Linalool | 0±0 | 0±0 | 3226.6±713 |
| (*R*)-Linalool (control) | 0±0 | 0±0 | 3275.8±350.3 |
