# Supplementary material for: A novel terpene synthase controls differences in anti-aphrodisiac pheromone production between closely related Heliconius butterflies
Source: PLoS Biol. 2021 Jan 19;19(1):e3001022. doi: 10.1371/journal.pbio.3001022 (PMC7815096; doi:10.1371/journal.pbio.3001022)
Supplement: S8 Table — Assays use precursors from different steps in the pathway (Fig 1) with HmelOS and HMEL037108g1 as well as HcydOS (Fig 3A and S4–S6 Tables). “None” stated if no compounds were detected in experimental treatments that were not also found in control treatments. (DOCX) [file pbio.3001022.s024.docx]

| Precursors | Enzyme | Products | Activity type |
| --- | --- | --- | --- |
| DMAPP + IPP | HmelOS | Trace (*E*)-β-Ocimene  Trace linalool  Trace nerolidol | Residual GPS  Monoterpene synthase  Sesquiterpene synthase |
| DMAPP + IPP | HcydOS | None | None |
| DMAPP + IPP | HMEL037108g1 | Trace linalool  Trace nerolidol | Residual GPS  Monoterpene synthase  Sesquiterpene synthase |
| GPP + IPP | HmelOS | (*E*)-β-Ocimene  Trace (Z)-β-Ocimene  Linalool | Monoterpene synthase |
| GPP + IPP | HcydOS | None | None |
| GPP + IPP | HMEL037108g1 | Trace (*E*)-β-Ocimene  Linalool | Monoterpene synthase |
| GPP | HmelOS | (*E*)-β-Ocimene  Trace (Z)-β-Ocimene  Linalool | Monoterpene synthase |
| GPP | HcydOS | None | None |
| GPP | HMEL037108g1 | Trace (*E*)-β-Ocimene  Linalool | Monoterpene synthase |
| FPP + IPP | HmelOS | None | None |
| FPP + IPP | HcydOS | None | None |
| FPP + IPP | HMEL037108g1 | Nerolidol | Sesquiterpene synthase |
