# Supplementary material for: A novel terpene synthase controls differences in anti-aphrodisiac pheromone production between closely related Heliconius butterflies
Source: PLoS Biol. 2021 Jan 19;19(1):e3001022. doi: 10.1371/journal.pbio.3001022 (PMC7815096; doi:10.1371/journal.pbio.3001022)
Supplement: S9 Table — Only residual IDS activity is detected, with small amounts of (E)-β-ocimene, linalool, and nerolidol produced from DMAPP and IPP. No other IDS activity is detected. High amounts of geraniol and farnesol in both experimental and control treatments is due to dephosphorylation of GPP and FPP, respectively. The main function of HmelOS is the production of (E)-β-ocimene from GPP. Mean amounts (ng) ± standard deviation for each compound across 3 replicates are shown. N = 3 for each treatment. Raw GC/MS data and quantification of each sample are available from OSF (https://osf.io/3z9tg/). DMAPP, dimethylallyl diphosphate; FPP, farnesyl diphosphate; GC/MS, gas chromatography/mass spectrometry; GPP, geranyl diphosphate; IDS, isoprenyl diphosphate synthase; IPP, isopentenyl diphosphate. (DOCX) [file pbio.3001022.s025.docx]

|  | (*E*)-β-Ocimene | (*Z*)-β-Ocimene | Linalool | Geraniol | Nerolidol | Farnesol |
| --- | --- | --- | --- | --- | --- | --- |
| DMAPP + IPP | 1.7±0.5 | 0±0 | 1±0.1 | 0±0 | 1.6±0.1 | 0±0 |
| DMAPP + IPP (control) | 0±0 | 0±0 | 0±0 | 0±0 | 0±0 | 0±0 |
| GPP + IPP | 325.3±17.4 | 11.3±0.7 | 109.2±10.5 | 1590.1±133.1 | 0±0 | 0±0 |
| GPP + IPP (control) | 2.9±0.2 | 0±0 | 17.6±0.8 | 2300.4±156 | 0±0 | 0±0 |
| FPP + IPP | 0±0 | 0±0 | 0±0 | 0±0 | 15.7±5.1 | 1320.3±114.5 |
| FPP + IPP (control) | 0±0 | 0±0 | 0±0 | 0±0 | 4±0.2 | 1582.0±65.6 |
