# Supplementary material for: A novel terpene synthase controls differences in anti-aphrodisiac pheromone production between closely related Heliconius butterflies
Source: PLoS Biol. 2021 Jan 19;19(1):e3001022. doi: 10.1371/journal.pbio.3001022 (PMC7815096; doi:10.1371/journal.pbio.3001022)
Supplement: S10 Table — TPS activity is again demonstrated by the production of linalool from FPP, and nerolidol from FPP. Only residual IDS activity is detected, by the presence of linalool and nerolidol in treatments with DMAPP and IPP, and nerolidol in the GPP treatment. Geraniol and farnesol are present due to dephosphorylation of remaining GPP and FPP in treatments. Mean amounts (ng) ± standard deviation for each compound across 3 replicates are shown. N = 3 for each treatment. Raw GC/MS data and quantification of each sample are available from OSF (https://osf.io/3z9tg/). DMAPP, dimethylallyl diphosphate; FPP, farnesyl diphosphate; GC/MS, gas chromatography/mass spectrometry; IDS, isoprenyl diphosphate synthase; IPP, isopentenyl diphosphate; TPS, terpene synthase. (DOCX) [file pbio.3001022.s026.docx]

|  | (*E*)-β-Ocimene | Linalool | Geraniol | Nerolidol | Farnesol |
| --- | --- | --- | --- | --- | --- |
| DMAPP + IPP | 0±0 | 3.9±1.6 | 0±0 | 3.5±0.9 | 0±0 |
| DMAPP + IPP (control) | 0±0 | 0±0 | 0±0 | 0±0 | 0±0 |
| GPP + IPP | 12.0±0.8 | 3208.4±261.5 | 290.0±24.5 | 3.6±0.7 | 0±0 |
| GPP + IPP (control) | 0±0 | 30.6±0.7 | 2117.0±184.9 | 0±0 | 0±0 |
| FPP + IPP | 0±0 | 0±0 | 0±0 | 1536.3±61.3 | 983.8±57.5 |
| FPP + IPP (control) | 0±0 | 0±0 | 0±0 | 4.9±5.0 | 1220.8±1105.8 |
