# Supplementary material for: A novel terpene synthase controls differences in anti-aphrodisiac pheromone production between closely related Heliconius butterflies
Source: PLoS Biol. 2021 Jan 19;19(1):e3001022. doi: 10.1371/journal.pbio.3001022 (PMC7815096; doi:10.1371/journal.pbio.3001022)
Supplement: S11 Table — We find no evidence for IDS activity. Geraniol and farnesol are present due to dephosphorylation of remaining GPP and FPP in treatments. Mean amounts (ng) ± standard deviation for each compound across 3 replicates are shown. N = 3 for each treatment, apart from the FPP + IPP (control) which has N = 2. Raw GC/MS data and quantification of each sample are available from OSF (https://osf.io/3z9tg/). FPP, farnesyl diphosphate; GC/MS, gas chromatography/mass spectrometry; GPP, geranyl diphosphate; IDS, isoprenyl diphosphate synthase; IPP, isopentenyl diphosphate. (DOCX) [file pbio.3001022.s027.docx]

|  | (*E*)-β-Ocimene | Linalool | Geraniol | Nerolidol | Farnesol |
| --- | --- | --- | --- | --- | --- |
| DMAPP + IPP | 0±0 | 0±0 | 0±0 | 0±0 | 0±0 |
| DMAPP + IPP (control) | 0±0 | 0±0 | 0±0 | 0±0 | 0±0 |
| GPP + IPP | 0±0 | 2.6±0.7 | 1261.2±135.7 | 0±0 | 0±0 |
| GPP + IPP (control) | 0±0 | 6.2±1.5 | 1190.8±112.4 | 0±0 | 0±0 |
| FPP + IPP | 0±0 | 7.1±1.7 | 0±0 | 1.0±0.4 | 531.6±24.7 |
| FPP + IPP (control) | 0±0 | 8.0±1.3 | 0±0 | 0.6±0.1 | 445.4±22.8 |
