# Supplementary material for: A novel terpene synthase controls differences in anti-aphrodisiac pheromone production between closely related Heliconius butterflies
Source: PLoS Biol. 2021 Jan 19;19(1):e3001022. doi: 10.1371/journal.pbio.3001022 (PMC7815096; doi:10.1371/journal.pbio.3001022)
Supplement: S12 Table — Whole genome sequencing samples of H. melpomene (MEL) and H. cydno (CYD) from which gene sequences were used for amino acid alignment (Resequenced_mel_cyd.fa). Sequences are available from OSF (https://osf.io/3z9tg/). (DOCX) [file pbio.3001022.s028.docx]

| SequenceID | EarthcapeID | Species | Longituge | Latitude | Accession |
| --- | --- | --- | --- | --- | --- |
| chi.CAM25091 | CAM025901 | *CYD* | 9.120000 | -79.702000 | SAMEA104585050 |
| chi.CAM25137 | CAM025137 | *CYD* | 9.120000 | -79.702000 | SAMEA104585051 |
| chi.CAM580 | CAM000580 | *CYD* | 9.120000 | -79.702000 | SAMEA104585044 |
| chi.CAM582 | CAM000582 | *CYD* | 9.120000 | -79.702000 | SAMEA104585045 |
| chi.CAM585 | CAM000585 | *CYD* | 9.120000 | -79.702000 | SAMEA104585047 |
| chi.CAM586 | CAM000586 | *CYD* | 9.120000 | -79.702000 | SAMEA104585048 |
| chi.CJ553 | CAM000553 | *CYD* | 9.171400 | -79.757300 | SAMEA1919256 |
| chi.CJ560 | CAM000560 | *CYD* | 9.171400 | -79.757300 | SAMEA1919265 |
| chi.CJ564 | CAM000564 | *CYD* | 9.171400 | -79.757300 | SAMEA1919278 |
| chi.CJ565 | CAM000565 | *CYD* | 9.171400 | -79.757300 | SAMEA1919262 |
| ros.CAM1841 | CAM011841 | *MEL* | 9.076000 | -79.659000 | SAMEA104585083 |
| ros.CAM1880 | CAM001880 | *MEL* | 9.076000 | -79.659000 | SAMEA104585084 |
| ros.CAM2045 | CAM002045 | *MEL* | 9.110300 | -79.690700 | SAMEA104585085 |
| ros.CAM2059 | CAM002059 | *MEL* | 9.110300 | -79.690700 | SAMEA104585086 |
| ros.CAM2519 | CAM002519 | *MEL* | 9.010900 | --79.547700 | SAMEA104585087 |
| ros.CAM2552 | CAM002552 | *MEL* | 9.010900 | --79.547700 | SAMEA104585088 |
| ros.CJ2071 | CAM002071 | *MEL* | 9.120600 | --79.696900 | SAMEA1919257 |
| ros.CJ531 | CAM000531 | *MEL* | 9.120600 | --79.696900 | SAMEA1919271 |
| ros.CJ533 | CAM000533 | *MEL* | 9.120600 | --79.696900 | SAMEA1919260 |
| ros.CJ546 | CAM000546 | *MEL* | 9.120600 | --79.696900 | SAMEA1919279 |
