# Supplementary material for: A novel terpene synthase controls differences in anti-aphrodisiac pheromone production between closely related Heliconius butterflies
Source: PLoS Biol. 2021 Jan 19;19(1):e3001022. doi: 10.1371/journal.pbio.3001022 (PMC7815096; doi:10.1371/journal.pbio.3001022)
Supplement: S13 Table — The predicted LCA sequence, as well as the HmelOS and HcydOS sequences used for ASR are shown for comparison. The amino acids at these sites are shown for a further 20 sequences from 10 individuals of each species (S12 Table, Resequenced_mel_cyd.fa). All totals do not add up to 20 due to incomplete sequencing reads. Sequences are available from OSF (https://osf.io/3z9tg/). ASR, ancestral state reconstruction; LCA, last common ancestor. (DOCX) [file pbio.3001022.s029.docx]

| Amino acid position | 9 | 10 | 57 | 79 | 109 | 119 | 122 | 123 | 265 |
| --- | --- | --- | --- | --- | --- | --- | --- | --- | --- |
| LCA | I | N | A | I | M | V | R | A | N |
| H. cydno | I | N | A | I | T | T | R | T | N |
| H. melpomene | V | K | V | V | M | V | K | A | Y |
| Other H. cydno | 20I | 14N, 6K | 20A | 20I | 17T, 3M | 20T | 20R | 20T | 18N) |
| Other H. melpomene | 9V, 7I | 16K | 14A, 6 | 14V, 6I | 20M | 20V | 19K, 1R | 20A | 20N |
