# Supplementary material for: A novel terpene synthase controls differences in anti-aphrodisiac pheromone production between closely related Heliconius butterflies
Source: PLoS Biol. 2021 Jan 19;19(1):e3001022. doi: 10.1371/journal.pbio.3001022 (PMC7815096; doi:10.1371/journal.pbio.3001022)
Supplement: S14 Table — (DOCX) [file pbio.3001022.s030.docx]

| Abbreviation | Full name |
| --- | --- |
| *A. gossypii* | *Aphis gossypii* |
| *A. grandis* | *Anthonomus grandis* |
| *A. thaliana* | *Arabidopsis thaliana* |
| *B. mori* | *Bombyx mori* |
| *B. terrestris* | *Bombus terrestris* |
| *C. fumiferana* | *Choristoneura fumiferana* |
| *C. reinhardtii* | *Chlamydomonas reinhardtii* |
| *C. unshiu* | *Citrus reinhardtii* |
| *D. melanogaster* | *Drosophila melanogaster* |
| *D. ponderosae* | *Dendroctonus ponderosae* |
| *F. fujikuroi* | *Fusarium fujikuroi* |
| *G. arboreum* | *Gossypium arboreum* |
| *G. biloba* | *Ginkgo biloba* |
| *H. lupulus* | *Humulus lupulus* |
| *H. melpomene* | *Heliconius melpomene* |
| *H. sapiens* | *Homo sapiens* |
| *I. pini* | *Ips pini* |
| *M. chamomilla* | *Matricaria chamomilla* |
| *M. domestica* | *Malus domestica* |
| *M. histrionica* | *Murgantia histrionica* |
| *M. lewisii* | *Mimulus lewisii* |
| *M. persicae* | *Myzus persicae* |
| *M. piperita* | *Mentha piperita* |
| *N. tabacum* | *Nicotiana tabacum* |
| *N. viridula* | *Nezara viridula* |
| *P. cochleariae* | *Phaedon cochleariae* |
| *P. striolata* | *Phyllotreta striolata* |
| *R. speratus* | *Reticulitermes speratus* |
| *S. cerevisiae* | *Saccharomyces cerevisiae* |
| *T. castaneum* | *Tribolium castaneum* |
