# Supplementary material for: A novel terpene synthase controls differences in anti-aphrodisiac pheromone production between closely related Heliconius butterflies
Source: PLoS Biol. 2021 Jan 19;19(1):e3001022. doi: 10.1371/journal.pbio.3001022 (PMC7815096; doi:10.1371/journal.pbio.3001022)
Supplement: S15 Table — The DNA sequence “CACC” was added to the 5′ end of the forward primer so that it was compatible with the plasmid vector. (DOCX) [file pbio.3001022.s031.docx]

| Gene | Primer sequence | Use |
| --- | --- | --- |
| *HMELOS* | Forward:  CACCATGTCAGAAACAGAAGTCC  Reverse:  TTAATTATCCTTCCAACTTAAAAGCGA | Amplification of transcript from cDNA library |
| *HMEL037108g1* | Forward:  CACCATGGACGTTCAGAAAATAAGC  Reverse:  TTAATTCGTTTTCCAAGAAAGAAGTTC | Amplification of transcript from cDNA library |
| *HCYDOS* | Forward:  CACCATGTCAGAAACAGAAGTGCATGTAATA  Reverse:  GCGAATGTAGCACTTCATCTATAAGAGGATT | Amplification of synthesised DNA (23bp missing from end of gene) |
| *HMEL037106g1* | Forward:  GTTAATTCGTTACACGTAGC  Reverse:  TAATCTGGAAATAGCGACC | Sequencing PCR products |
| *HMEL037108g1* | Forward:  CTAACTCTTAACGCCTCG  Reverse:  TTATATCCTCGCAGAAATCG | Sequencing PCR products |
| Both | Forward:  AATACGACTCACTATAGGGG  Reverse:  GGTTAGGGATAGGCTTACC | Sequencing insert in plasmid |
