# Supplementary material for: A novel terpene synthase controls differences in anti-aphrodisiac pheromone production between closely related Heliconius butterflies
Source: PLoS Biol. 2021 Jan 19;19(1):e3001022. doi: 10.1371/journal.pbio.3001022 (PMC7815096; doi:10.1371/journal.pbio.3001022)
Supplement: S16 Fig — The 2 aspartate-rich motifs are labelled FARM and SARM. Stars show residues identified as conserved between insect TPSs [28]. Hmel, H. melpomene; Mhistr, Murgantia histrionica; Ip, Ips pini; Pstri, Phyllotreta striolata; Hmel_TPS_1, HmelOS; Hmel_TPS_2, HMEL037108g1. Raw data and results files are available from OSF (https://osf.io/3z9tg/). GGPPS, geranylgeranyl diphosphate synthase; TPS, terpene synthase. (PDF) [file pbio.3001022.s016.pdf]

```
Hmel_GGPPS    1 -------------------------------------------------------MSLV-
Hmel_TPS_2    1 -------------------------------------------------------MDVQK
Hmel_TPS_1    1 ---------------------------MSE---------------------TEVHVVKVN
Mhistr_TPS    1 ------------MVSIAAKSLPKLSGAVFG-------------------------QFSRR
Ip_IDS_TPS    1 MFKLAQR---------LPKSVSSLGSQLSKNAPNQLAATTSQLINTPGIRHKSRSSAVP
Pstri_TPS3    1 --MLYVLKNYNLNYSIVSKVPLHFRSLC----------------------------SLLQ
Pstri_TPS4    1 MFAICKVVNYYSSCRIIPKVSGNFGTLLQRSF---------------------NRAFSCE
Pstri_TPS1    1 MFLLPRLKNFTRSNSPARKLFSPK----SN-------------------------SFS--
Pstri_TPS2    1 ------------------------------------------------------------

Hmel_GGPPS    5 KSKDGDKNQD------------------EKLLMP--FTYIQQVPGKQIRSKLTLAFNYWL
Hmel_TPS_2    6 ISENSDLYME------------------NELLLP--YNHVLQVSGKQMRMKIVKALNHWL
Hmel_TPS_1   13 GKENDDLFLE------------------KEILAP--FSHICQVKGKQLRIKIMRAFNHWL
Mhistr_TPS   24 KQLIQRHWL--------DTRTDQYDVLRRIVVPECKNIASDVPEYPERIEKLLYYTNPA
Ip_IDS_TPS   52 SSLSKSMYDHNEEMKAAMKYMDEIYPEVMGQIEK--VPQYEEIKPILVRLREAIDYTVPY
Pstri_TPS3   31 KK-NNRPLVD--I-SVEEGPLRSVYPAIREEIIEEHLVLK-GNSELRDRCEKLLDYNANV
Pstri_TPS4   40 IE-ANEPFVD--L-FSEEEHLKSMLPAVKEEIIEEHLVLHKDNKEIRNRCEKLIDYNINT
Pstri_TPS1   30 ----STPHDD-GFFKHEMDELKTYYPLMVQDLTDA-ISQYKQFPGLLERFPVLMDYTVTH
Pstri_TPS2    1 ------MYTQ-DFFLDEYNEIMAYYPKVIEQVRN--WTFIKEYQGLPDRYTNYMNYNVDV

                                                                    FARM
Hmel_GGPPS   45 -----------------RV------SDD---KLRAIGEIVQMLHNSSLLLDDIQDNSILR
Hmel_TPS_2   46 -----------------KV------PEG---DMENIVNLIHMIHAASLLLDDIQDDSKLR
Hmel_TPS_1   53 -----------------QA------SEV---DVMKALGIVNSLHVASLLIDDVQDDSTLR
Mhistr_TPS   76 F---SDAWNFTTELIYRTVADESHQTEENITKMYLIRATMDLLFTMSAVLDDISDRSEFR
Ip_IDS_TPS  110 GKR-F--KGVHIVSHFKLLADPKFITPENVKLSGVLGWCAEIIQAYFCMLDDIMDDSDTR
Pstri_TPS3   86 ETP-FLSASLIFLHTYKLLEKPSLLNHENLKKAYILAWCHKLIHSSININDDIIDRSNIR
Pstri_TPS4   96 ESK-FLTFPLIFLRTYKLLEKPALLNDENLKKACILAWCHRLIHASVIISDDIVDDSEMR
Pstri_TPS1   84 DDPYFLSSAVLPLYFYKAVEESDKLTEENIKRACLMSWAYRTLETSQIIVDDILDKSEVR
Pstri_TPS2   52 KDTKRMATALFTLYSYKHLEQPEKQTDENLRKAIAMAWAFRMAEASQLTLDDVLDNSLTR

Hmel_GGPPS   79 RGIPVAHSIYGIAST--INAANY---VIIIALEKTLKLGHPLA---TTVYTEQLLELHRG
Hmel_TPS_2   80 RGLPAAHNVYGVPLT--LNASCH---AIFLVLIKSYDIN-PKV---SKIMVESFLWGLRG
Hmel_TPS_1   87 RGMPAAHCVYGVPLT--VNTSLH---AMFLVLEEAFAVD-PKA---AKLLVEDFLEMCRG
Mhistr_TPS  133 KGKKGWHMICQGGESTALYDGTQMGLFPLYLLKQY-FKNDPGYSRILETVVMTYIKLTIG
Ip_IDS_TPS  167 RGKPTWYKLPGIGLN-AVTDVCLMEMFTFELLKRY-FPKHPSYADIHEILRNLLFLTHMG
Pstri_TPS3  145 YNKTTWYQLPDVGKDAAIVDAAFLLNGAIFLLQNH-LRCLPHQYIMQKHFLRAHAIMNLS
Pstri_TPS4  155 YNKTTWCKLPDVGKEDAITDAAFLLTGAIFLLQNH-LRHHPHNFILQKHFLRGYAFINVS
Pstri_TPS1  144 YNKPAWYKKDGVSMELTILDSHYLATGAYMVLTKR-LAGHPCCLDILDLYAEEMFVMIIA
Pstri_TPS2  112 YMKPAWHKLEGTNN--AVLDAFFVENAAYLILQEE-MRDHPQFLNIVKLLKEYYLMLVVG

Hmel_GGPPS  131 QGLEIYWR------DNFQCPTEDEYKEMTMKKT--GGLFM-----LAIRLMQLFSDNKS-
Hmel_TPS_2  131 QGMDLHWR------ENFVCPTVEQYMKMVELKT--GYMFS-----GAYEVMQLYSDNKT-
Hmel_TPS_1  138 QGIDIYWR------DHLICPTEGQYYKMLEQKT--GHFFL-----MGVRMMQLFSCNKT-
Mhistr_TPS  192 QTIDV------LGQFKKSPSM-AEYKRINYYKA--GQFVA-AGSELAVIHAGITSQDLID
Ip_IDS_TPS  225 QGYDFTFIDPVTRKINFNDFTEENYTKLCRYKIIFS--TFHNTLELTSAMANVYDPKKIK
Pstri_TPS3  204 NIPE-------LKKIKIN---E-----GDKHQLDIKFYSYN-IISTAMFLANVTDGYLHE
Pstri_TPS4  214 YMMD-------NRKHHIN---E-----LEKYQVHTKLYFSN-LFSTAMYLANVENDYWQK
Pstri_TPS1  203 QYMD-------IKKLDLKDFQK-----LVRHRFDKALYVFNGSARSGLYLANVRDRETHD
Pstri_TPS2  169 QYLD-------MRSIPFEKSFE-----LLKYRNIKGYYITNMPIRGSMYLANIDNPDYHA

                                 SARM
Hmel_GGPPS  177 DFTKLSSLLGLYFQIRDDYCNLCLREY------------SENKSYCEDLTEGKFSFPIIH
Hmel_TPS_2  177 DYSKIISIMGRLFQIRDDYCNLKHREALEEWPGEDDIEVIRDHDFCEDITEGKFSFPVIH
Hmel_TPS_1  184 DYSELVLLMGRYFQIRDDYCNLSQQEALEEWPGAEDLQVCKNDSFCEDITEGKISLPIIH
Mhistr_TPS  242 KTVEIFTIAGQIIQTWDDFNDYYSSSEQN-------GK------LSCDFMNAGTTWVSAK
Ip_IDS_TPS  283 QLDPVLMRIGMMHQSQNDFKDLYRDQGEV-------LKQAEKSVLGTDIKTGQLTWFAQK
Pstri_TPS3  248 VCEEICNDLSRFIRIEDDVVDLYDSEGNI-------RKT-----SCTDISLGRPSWLTME
Pstri_TPS4  258 VSDEICNDISQYLKVEDDVIDLYDSKGKI-------R-------KCTDISLGRPSWFLME
Pstri_TPS1  251 CMKKFSVPMSRFFQVQNDFSGVFEEESKF-------Q-N-----SCPDIVNGRNSWLVTT
Pstri_TPS2  217 KVEEILRISGEWIIIQNDYQEVFLPTSEN-------K-K-----DRRDIQQGTNTWCLAK

Hmel_GGPPS  225 AIQNQKGDN-------------QVLHILRQRTRDVEVKRYCISLLEKFGSFQYTRDCLQE
Hmel_TPS_2  237 ALSTPEGK--------------PILNILKQRTRDVQLKKYCVSLMEKIGSLQHTCDVLDK
Hmel_TPS_1  244 ALQTKKAG--------------IVMNILRQKTRDMYLKKYCVSTLEEIGSLQYTRNVLEK
Mhistr_TPS  289 AMEVFTPSQAVKFMECYGSDDQSKMKTVQELYDEIDMPKLYTEYVLEN---------Y--
Ip_IDS_TPS  336 ALSICNDRQRKIIMDNYGKEDNKNSEAVREVYEELDLKGKFMEFEEES---------F--
Pstri_TPS3  296 AYKKGSAAQKKILEENFGKNNEESTEKIYSIFEDLQLLDVYKKLSDEF---------Y--
Pstri_TPS4  304 AYKRANAGQRKILEENFHKNNEESVEKLYSIFQELELLEVYRKFTDNF---------Y--
Pstri_TPS1  298 ALKMANPAQRKVIEENYGNGDAESARKVMQVYEDLKLKDVHDRRTEEF---------L--
Pstri_TPS2  264 ALELASESQMKVLKENYGKNDDESAMKIEEIYRDLKLDEIYLKIEEEY---------F--

Hmel_GGPPS  272 LDNEARAEVQRLGGNPHLEDLLDELLSWREDKKSAVNEE-------------
Hmel_TPS_2  283 LDQEAREEVARLGGNPEMIAVLDELLSWKTN---------------------
Hmel_TPS_1  290 LDLEIRAEVARLGGNPLIDEVLHSLLSWKDN---------------------
Mhistr_TPS  338 --NRCETLIKEL-PHDRLREACSSYMEWLVVRETPDEDSEHKVALCLNISG
Ip_IDS_TPS  385 --EWLKKEIPKI-NNGIPHKVFQDYTYGVFKRRPE-----------------
Pstri_TPS3  345 --EQAI---YKI-QKKLPKSKMQDALLDLLTLIVNHKCM-------------
Pstri_TPS4  353 --AQEI---SKI-REKIPKSIMQDILINLVNLAVNHKLRY------------
Pstri_TPS1  347 --GEMREIVENF-PERIPKQPFHDIVRQLALNKLYS----------------
Pstri_TPS2  313 --EKVNRRLDIL-PNILPKSFFWNMMHIIKNEYMNG----------------
```
